# Cell death-related biomarker SLC2A1 has a significant role in prognosis prediction and immunotherapy efficacy evaluation in pan-cancer

**DOI:** 10.3389/fgene.2022.1068462

**Published:** 2023-01-11

**Authors:** Yuhang Wang, Kai Wang, Han Zhang, Xiaoteng Jia, Xin Li, Shuai Sun, Daqiang Sun

**Affiliations:** ^1^ Graduate School, Tianjin Medical University, Tianjin, China; ^2^ Clinical School of Thoracic, Tianjin Medical University, Tianjin, China; ^3^ Department of Thoracic Surgery, Tianjin Chest Hospital of Tianjin University, Tianjin, China

**Keywords:** SLC2A1, pan-cancer, prognostic biomarker, immunotherapy, tumor immune microenvironment

## Abstract

**Introduction:** SLC2A1, a member of the SLC transporter family, is involved in a variety of cell death modalities and has been found to be associated with the prognosis and immune microenvironment of a variety of tumors. However, there is a lack of systematic and comprehensive studies on the role of SLC2A1 in pan-cancer.

**Methods:** The mRNA, promoter methylation, and protein expression levels of SLC2A1 in pan-cancer were comprehensively evaluated using GEPIA2.0, TIMER2.0, and UALCAN databases. UCSCXenaShiny based on the cancer genomic atlas pan-cancer data and GEPIA2.0 database were used to assess the prognostic significance of SLC2A1 in pan-cancer. Genetic alterations in *SLC2A1* were also evaluated using cBioPortal. The relevance of SLC2A1 to immune infiltrating cells in pan-cancer was evaluated using the XCELL algorithm in combination with the TIMER2.0 database. The correlation of SLC2A1 with the efficacy of immune checkpoint blocker (ICB) therapy was evaluated using the tumor immune dysfunction and exclusion (TIDE) score. The correlation of SLC2A1 with numerous immune-related markers was also evaluated using the TISIDB database. The correlation of SLC2A1 with tumor biological function was evaluated at the single-cell level using the CancerSEA database. Finally, the biological function of SLC2A1 was comprehensively evaluated using gene set enrichment analysis (GSEA) and protein interaction networks.

**Results:** SLC2A1 expression is aberrant in a variety of tumors and is strongly associated with the prognosis of several cancers. SLC2A1 is significantly associated with a variety of immune infiltrating cells including CD8^+^ T cells, myeloid-derived suppressor cells and macrophages in a variety of tumors. Meanwhile, the expression of SLC2A1 significantly correlated with multiple immune-related markers. In addition, SLC2A1 can also predict the effect of immune checkpoint blocker therapy in some tumors. In a functional analysis, SLC2A1 was significantly associated with hypoxia, epithelial-mesenchymal transition, mTORC1 signaling, and multiple metabolic pathways in pan-cancer.

**Conclusion:** Our study systematically and comprehensively summarizes the prognostic significance and immune-related role of SLC2A1 in pan-cancer and reveals the potential mechanism of SLC2A1 in regulating the tumor microenvironment and tumor behavior, providing a new effective pan-applicable biomarker for prognostic prediction and the evaluation of immunotherapeutic strategies for tumors.

## 1 Introduction

In recent years, the incidence and mortality rates of cancer have been gradually increasing, having a strong negative impact on human health and social development (Siegel et al., 2022). With the rise of targeted therapy and immunotherapy, an increasing number of cancers can be cured (Abbott and Ustoyev, 2019). However, drug resistance remains a problem that cannot be ignored in immunotherapy (O'Donnell et al., 2019; Jackson et al., 2019). With the in-depth research on tumor immunity, there is an urgent need to discover more biomarkers to assist in the diagnosis, evaluation, and treatment of cancer.

The SLC transporter family, which contains over 300 members, plays a significant role in the absorption of various nutrients and drugs by cells (Lin et al., 2015; Liu, 2019). SLC2A1 is a member of the SLC transporter family, which is mainly involved in encoding a glucose transporter protein present in cell membranes and cell surfaces. Meanwhile, SLC2A1 (GLUT1) is also a variety of programmed cell death-related genes that play important roles in ferroptosis (Zhou and Bao, 2020), anoikis (Chen et al., 2021a), necroptosis (Chen et al., 2022), and autophagy (Pei et al., 2022). In previous studies, we explored its role as a prognostic and immunotherapeutic marker in lung adenocarcinoma, finding it to be aberrantly expressed in numerous cancers (Wang et al., 2022). Additionally, SLC2A1 also has prognostic significance or an immune marker role in many other cancers. Xiao et al. explored the biological function of SLC2A1 in prostate cancer and found that it affects prostate cancer development by regulating cellular glycolysis and proliferation (Xiao et al., 2018). Wu et al. found that SLC2A1 inhibition blocks the growth of RB1-positive triple-negative breast cancer (Wu et al., 2020). Min et al. found that SLC2A1 improved the survival of gastric cancer patients by suppressing CD8^+^ T cells and B cells (Min et al., 2021). However, research on SLC2A1 is still confined to a few cancer species. There is no comprehensive and systematic analysis of its role in pan-cancer, and its role in the tumor immune microenvironment and potential mechanisms have not been fully explored.

In the present study, we performed a comprehensive pan-cancer analysis of the role played by SLC2A1 in the development and progression of 33 cancers and its possible mechanisms. We analyzed the expression of SLC2A1 in different cancers and the association between its expression and cancer prognosis, immune cell infiltration, immune-related marker expression, and tumor functional status. Furthermore, we performed functional enrichment analysis of SLC2A1-related genes and constructed protein interaction networks. Collectively, our study reveals the role of SLC2A1 as a powerful prognostic marker predicting immunotherapeutic efficacy for pan-cancer and explores its potential mechanisms.

## 2 Methods and materials

### 2.1 Data collection

Except for the special annotation of data source, the RNA sequencing (RNA-seq), DNA methylation beta value, copy number segment data and related sample annotation data of 33 cancer types used in this study were downloaded from the cancer genomic atlas (TCGA) (Tomczak et al., 2015).

### 2.2 Expression evaluations of SLC2A1 based on public databases

Tumor Immune Estimation Resource, version 2.0 (TIMER2.0) (Li et al., 2020) and Gene Expression Profiling Interactive Analysis, version 2.0 (GEPIA2.0) (Tang et al., 2017) were applied to compare SLC2A1 expression in tumor tissue and corresponding normal tissues. In GEPIA2, the matched TCGA normal and genotype-tissue expression dataset (GTEx) data were analyzed, and the screening criteria were set as *p*-value < 0.05 and cutoff of |Log_2_|FoldChange (FC) | > 1. Furthermore, we utilized the GEPIA2 database to analyze the association between SLC2A1 expression and pathological stages across TCGA cancers. The DNA methylation levels of the *SLC2A1* promoter were compared using TCGA data in The University of Alabama at Birmingham CANcer data analysis Portal (UALCAN) (Chandrashekar et al., 2017). Correlation analysis was performed using RNA sequencing data with DNA methylation data obtained from TCGA. The region of the DNA promoter was defined as TSS200-1,500. The protein expression levels and phosphorylation of SLC2A1 in normal and primary tumor tissues were compared in UALCAN using Clinical Proteomic Tumor Analysis Consortium (CPTAC) data and protein alterations of SLC2A1 were investigated using the PhosphpSitePlus database (Hornbeck et al., 2015).

### 2.3 Genetic alteration analysis of SLC2A1 based on public databases

The cBioPortal platform (Cerami et al., 2012) was applied to evaluate the mutation and copy number alteration (CNA) frequency of SLC2A1 across TCGA cancers. Then, we analyzed the relationship between different SLC2A1 mutation and CNA statuses and its expression. Additionally, we compared the frequency of other genetic alterations in the SLC2A1-altered group with that of the non-altered group, along with the survival of these two groups. Correlation analysis was subsequently performed using CNV data downloaded from TCGA with RNA sequencing data. In addition to this, we conducted a further analysis of the relationship between the CNA species of SLC2A1 and tumor prognosis in pan-cancer using UCSCXenaShiny (https://hiplot-academic.com/advance/ucsc-xena-shiny) (Wang et al., 2021).

### 2.4 Prognostic analysis of SLC2A1

The prognostic values of SLC2A1, including overall survival (OS) and disease-free survival (DFS), were evaluated across TCGA cancers in GEPIA2. *p* < 0.05 was considered to denote statistical significance. Additionally, the cyclic univariate cox regression analysis based on TCGA Pan-Cancer data to further confirm the prognostic values of SLC2A1 was conducted by UCSCXenaShiny. And the forest diagrams were used to display the *p*-value, Hazard Ratio (HR), and 95% confidence interval (CI) of each cancer using Xiantao Academic Tool (https://www.xiantao.love/).

### 2.5 Relationship between SLC2A1 and tumor immunity

The R package “Immunedeconv” (Sturm et al., 2020) was applied to calculate the immune infiltration scores of all TCGA cancers by XCELL algorithms (Aran et al., 2017). The Spearman’s correlation test was then used to assess the correlation between SLC2A1 expression and immune infiltration scores. Results were visualized using the “ggplot2” R package. Additionally, the correlation between SLC2A1 expression and immune checkpoint-related gene expression was also analyzed by the above method. The potential immune checkpoint blocker (ICB) response was predicted with the tumor immune dysfunction and exclusion (TIDE) algorithm (http://tide.dfci.harvard.edu/) (Jiang et al., 2018) and visualized using R package “ggplot2” and “ggpubr” (Kassambara, 2020).

Then, purity-adjusted Spearman correlations between SLC2A1 expression and the infiltration level of tumor immune cells were conducted *via* TIMER2 to further validated the R analysis. Results with correlation coefficients greater than .5 were displayed.

Furthermore, we evaluated the correlation of SLC2A1 expression with the expression of immune-related biomarkers (such as immunoinhibitors, immunostimulators, and major histocompatibility complex (MHC) molecules) in the TISIDB database (http://cis.hku.hk/TISIDB/index.php) (Ru et al., 2019).

### 2.6 Functional correlation analysis of SLC2A1 at the single-cell level

We investigated the CancerSEA database (Yuan et al., 2019) to explore the expression of SLC2A1 at the single-cell level in different cancers and its relationship with the tumor functional status at the single-cell level. Correlation data between SLC2A1 expression and biological function in different cancers were downloaded on CancerSEA and visualized using a correlation bubble heatmap using the “ggplot2” R package. Results with significant correlations in each single-cell sequencing dataset and their t-SNE diagrams are obtained from CancerSEA and shown separately.

### 2.7 Gene set enrichment analysis and protein-protein interaction network of SLC2A1

To further explore the biological function of SLC2A1 in cancer, differential expression analysis of RNAseq data in TCGA pan-cancer was performed based on SLC2A1 expression grouping. Gene set enrichment analysis (GSEA) (Subramanian et al., 2005) was performed according to the results of differential expression analysis. Then, the correlation between SLC2A1 and the enrichment scores of 50 HALLMARK pathways of cancer (Liberzon et al., 2015) in different tumors were analyzed and visualized by bubble heatmap. GSEA was performed using “GSVA” (Hänzelmann et al., 2013), “ggpubr”, “data.table” (Dowle and Srinivasan, 2021), “ggplot2”, “limma” (Ritchie et al., 2015), and “clusterProfiler” (Wu et al., 2021) R packages. Additionally, to further verify the relationship between SLC2A1 and programmed cell death such as ferroptosis, loss of anoikis, autophagy, and necroptosis, we also performed correlation analysis of SLC2A1 with key genes in these cell death modalities.

A SLC2A1-related protein-protein interaction network (PPI) was constructed using the STRING database (Szklarczyk et al., 2021), and the max number of the first shell interactor was set as no more than 50, while that of the second shell was set as none. Cytoscape was used for image adjustment and beautification (Shannon et al., 2003). Gene Ontology (GO) (Gene Ontology Consortium Blake et al., 2013) and Kyoto Encyclopedia of Genes and Genomes (KEGG) (Kanehisa and Goto, 2000) functional enrichment analyses were conducted to explore the biological role of SLC2A1-related genes in PPI. Additionally, a disease-specific enrichment analysis was conducted using Metascape (Zhou et al., 2019).

### 2.8 Statistical analysis

All the analysis methods and R packages were implemented using R version 4.2.1, except for the online website tools mentioned above. For all analyses, the low and high SLC2A1 expression groups were established according to the median SLC2A1 mRNA expression value in the selected dataset. Public databases were used under default settings, and all other correlation analysis methods were Spearman correlation analysis. *p* values less than .05 were considered statistically significant (*p < 0.05, **p < 0.01, ***p < 0.001).

## 3 Results

### 3.1 SLC2A1 was aberrated in most cancers at the transcriptome level

**FIGURE 1:**
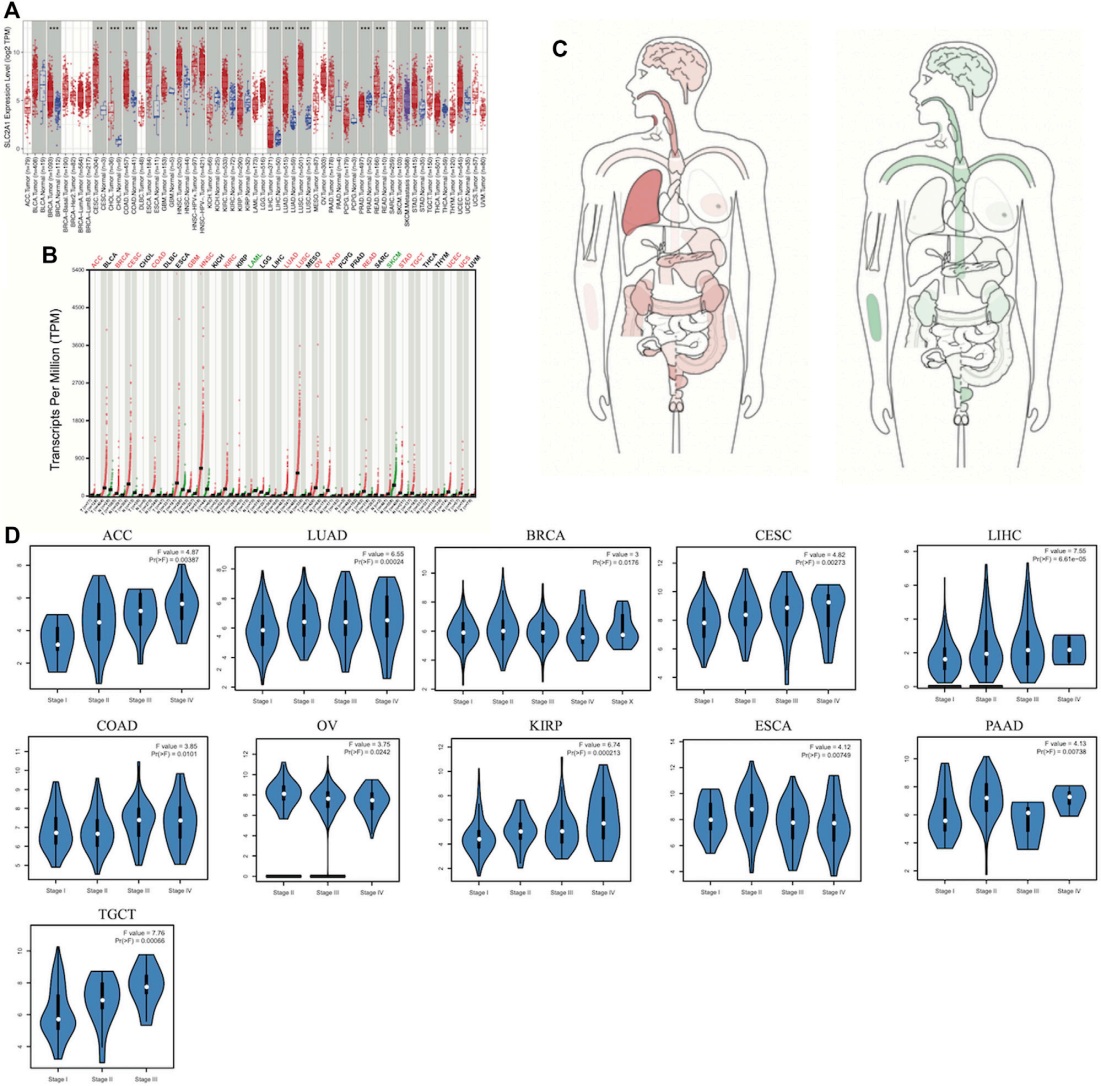
Aberrant transcriptome profiles and DNA methylation level of SLC2A1 in pan-cancer **(A)** mRNA level of PDHA1 performed by the TIMER2 database **(B)** GEPIA2.0 depicted the SLC2A1 expression in matched tumors group and normal group **(C)** The median expression of SLC2A1 of tumor and normal samples in bodymap from GEPIA2.0 **(D)** SLC2A1 overexpression significantly correlated with pathological stages in ACC, BRCA, CESC, COAD, ESCA, LUAD, LIHC, OV, KIRP, PAAD, TGCT *p < 0.05; **p < 0.01; ***p < 0.001.

In our study, TIMER2.0, GEPIA2.0, and UALCAN platforms were used to research differential SLC2A1 expression in tumors and corresponding normal tissues. Among the results of unpaired expression analysis provided by TIMER2.0, SLC2A1 was significantly upregulated in 14 cancer types and downregulated in two from TCGA (Figure 1A). SLC2A1 expression was increased in breast cancer (BRCA), cervical squamous cell carcinoma and endocervical adenocarcinoma (CESC), cholangiocarcinoma (CHOL), colon adenocarcinoma (COAD), esophageal carcinoma (ESCA), head and neck squamous carcinoma (HNSC), kidney renal clear cell carcinoma (KIRC), kidney renal papillary cell carcinoma (KIRP), liver hepatocellular carcinoma (LIHC), lung adenocarcinoma (LUAD), lung squamous cell carcinoma (LUSC), rectal adenocarcinoma (READ), stomach adenocarcinoma (STAD), thyroid carcinoma (THCA), and uterine corpus endometrial carcinoma (UCEC). In contrast, its expression was decreased in kidney chromophobe (KICH) and prostate adenocarcinoma (PRAD). In the expression analysis results of matched tumors and normal tissues provided by GEPIA2.0, SLC2A1 was significantly upregulated in adrenocortical carcinoma (ACC), BRCA, CESC, COAD, glioblastoma multiforme (GBM), HNSC, KIRC, LUAD, LUSC, ovarian serous cystadenocarcinoma (OV), pancreatic adenocarcinoma (PAAD), READ, STAD, testicular germ cell tumors (TGCT), UCEC, and uterine carcinosarcoma (UCS) (Figure 1B). On the contrary, the expression level of SLC2A1 was downregulated in acute myeloid leukemia (LAML) and skin cutaneous melanoma (SKCM). The median expression of SLC2A1 in tumor and normal samples in a body map from GEPIA2.0 was also displayed (Figure 1C).

Thereafter, we investigated SLC2A1 expression at different pathological stages *via* the GEPIA2.0 database. As shown in the violin plots, SLC2A1 overexpression significantly correlated with pathological stages in ACC, BRCA, CESC, COAD, ESCA, LUAD, LIHC, OV, KIRP, PAAD, and TGCT (Figure 1D). Similarly, as shown in Supplementary Figure S1, SLC2A1 expression was not significantly associated with pathological stages in other cancer types.

### 3.2 *SLC2A1* promoter methylation was aberrantly expressed in multiple cancers and correlated with SLC2A1 transcriptome expression levels

**FIGURE 2:**
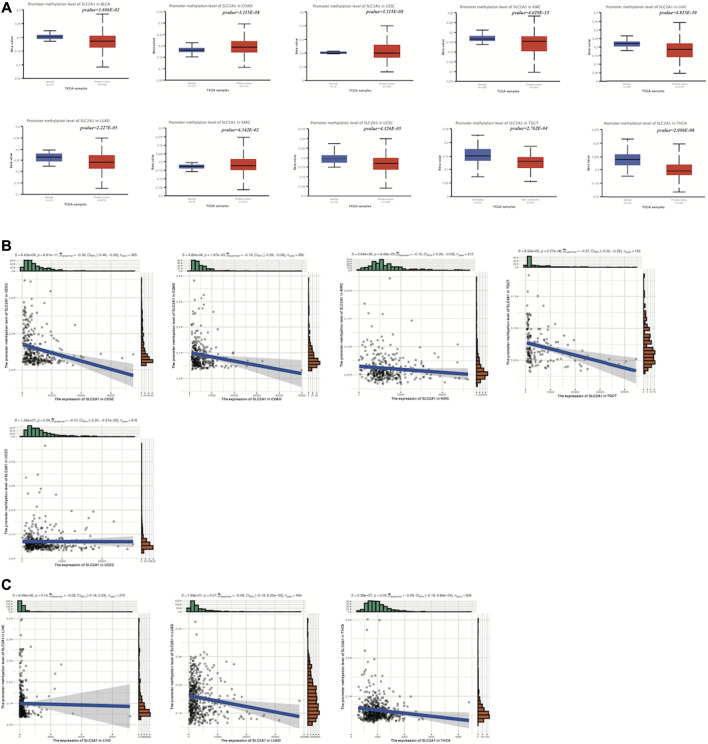
SLC2A1 promoter methylation was aberrantly expressed in multiple cancers and correlated with SLC2A1 transcriptome expression levels **(A)** SLC2A1 promoter methylation levels were aberrantly expressed in BLCA, COAD, CESC, KIRC, LIHC, LUAD, SARC, UCEC, TGCT and THCA **(B)** The transcriptome expression levels of SLC2A1 were significantly correlated with the promoter methylation levels of SLC2A1 in CESC, COAD, LUAD, TGCT and THCA **(C)** The transcriptome expression levels of SLC2A1 were not correlated with the promoter methylation levels of SLC2A1 in UCEC, LIHC and KIRC.

Since DNA methylation can control gene expression without causing any alteration in the genomic sequence, we investigated the methylation level of the *SLC2A1* promoter at the pan-cancer level with TCGA data in UALCAN to investigate whether the abnormal expression level of SLC2A1 was related to DNA methylation. The results showed that both SLC2A1 promoter methylation and SLC2A1 transcriptome levels were aberrantly expressed in COAD, CESC, KIRC, LIHC, LUAD, UCEC, TGCT and THCA (Figure 2A). Further analysis showed that the transcriptome expression levels of SLC2A1 were significantly correlated with the promoter methylation levels of SLC2A1 in CESC, COAD, LUAD, TGCT and THCA, suggesting that the aberrant transcriptome expression of SLC2A1 was indeed associated with DNA promoter methylation in these tumors (Figure 2B). In contrast, in UCEC, LIHC and KIRC, although there was both aberrant expression of SLC2A1 transcriptome level and aberrant expression of SLC2A1 promoter methylation, correlation analysis showed no association between the two (Figure 2C). In other tumors, *SLC2A1* promoter methylation levels were not significantly abnormal (Supplementary Figure S2). This suggested that the aberrant expression of SLC2A1 at the transcriptome level was not exclusively due to DNA promoter methylation, and we therefore explored the genetic alterations of SLC2A1 in pan-cancer.

### 3.3 Genetic alterations in SLC2A1 affected its expression at the transcriptome level and correlated with tumor prognosis

**FIGURE 3:**
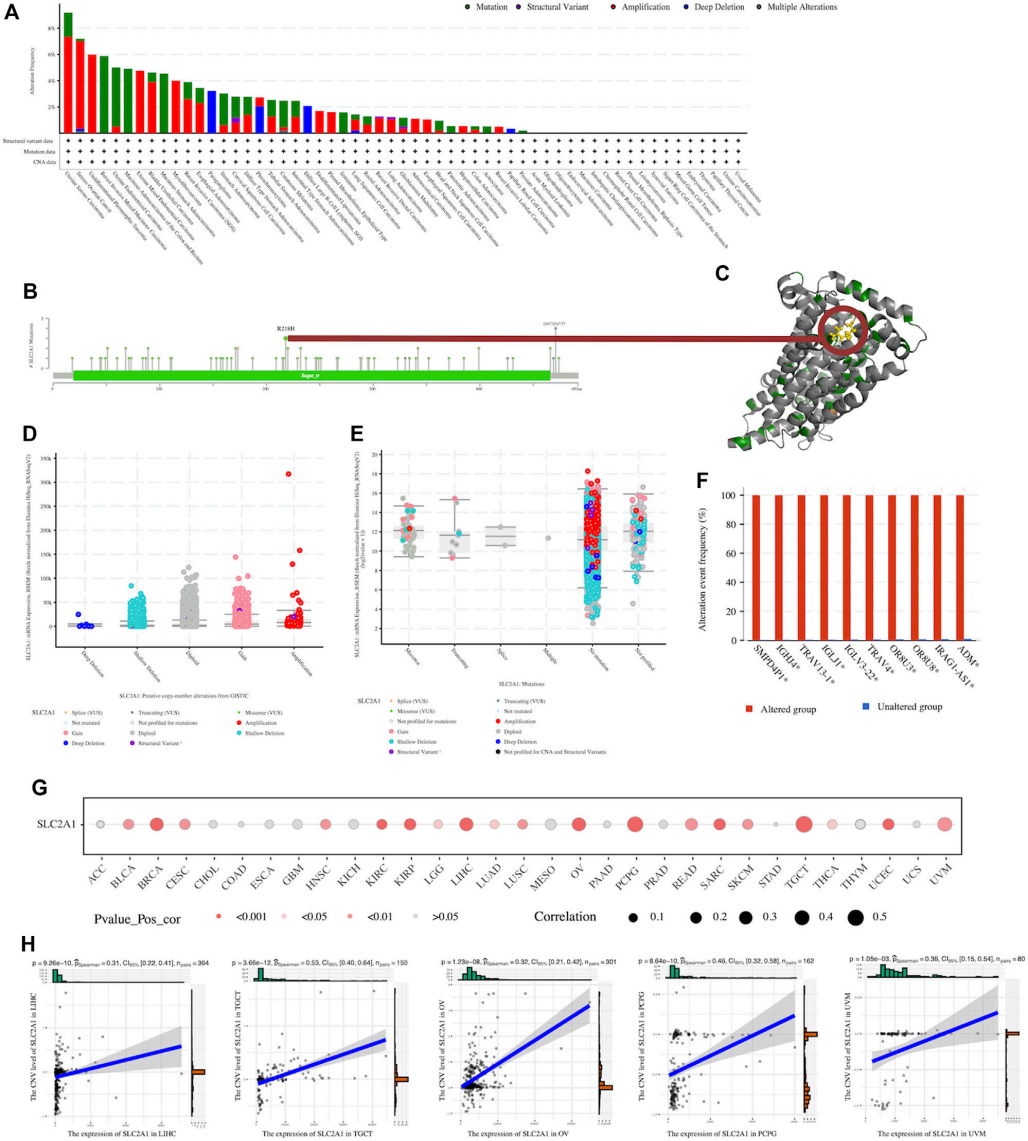
Genetic alteration of SLC2A1 in pan-cancer **(A)** Genetic alteration status of SLC2A1 in pan-cancers was performed by cBioPortal **(B)** Main genetic alterations of SLC2A1 **(C)** R218H mutation site was visualized in the 3D structure of SLC2A1 protein **(D)** The association between the copy number alteration types of SLC2A1 and the expression of SLC2A1 from GISTIC **(E)** The association between the mutation types of SLC2A1 and the expression of SLC2A1 from GISTIC **(F)**The top 10 genes with co-alteration frequency. GISTIC, genomic identification of significant targets in cancer **(G)** The expression level of SLC2A1 and the CNV level of *SLC2A1* were significantly correlated in 19 cancers **(H)** In LIHC, TGCT, OV, PCPG and UVM, the correlation coefficients between these two exceeded 0.3.

We explored the *SLC2A1* genetic alterations in human tumor samples *via* the cBioPortal tool, using the TCGA Pan-Cancer dataset to complete this analysis. The alteration frequency of SLC2A1 (9.17% of 109 cases) was the highest in uterine serous carcinoma (Figure 3A). Furthermore, we explored the mutation types and mutation sites within the *SLC2A1* sequence. There were 80 mutations in the full sequence of *SLC2A1*. There were 63 missense mutations, nine truncating mutations, three splice mutations, and 5 S V/fusion mutations (Figure 3B). The mutation frequency of R218H, which was mainly located within the Sugar (and other) transporter (19–466) domain, was the highest among all mutated loci, occurring in three tumor patients (Figure 3B, C). The expression level of SLC2A1 did not differ significantly among the different *SLC2A1* genetic alteration types (CNAs and mutations) (Figure 3D, E). Although the frequency of *SLC2A1* alterations was low, occurring in only 2% of the 10,953 patients, the difference in the incidence of genetic alterations between *SLC2A1*-altered and -unaltered groups was surprisingly striking. In the *SLC2A1*-altered group, the frequency of genomic alteration co-occurrence was significantly higher, and the top five genes with a high co-alteration frequency were *SMPD4P1*, *IGHJ4*, *TRAV13-1*, *IGLJ1*, and *TRAV4*. (Figure 3F). And from the results of the correlation analysis between the expression level of SLC2A1 and the CNV level of *SLC2A1*, they were significantly correlated in 19 cancers (Figure 3G), including LIHC, KIRC and UCEC, where previous DNA promoter methylation failed to explain the abnormal expression of SLC2A1. In LIHC, TGCT, OV, PCPG and UVM, the correlation coefficients between these two exceeded .3 (Figure 3H). Combined with the results of previous analysis of DNA promoter methylation, we concluded that the genetic and epigenetic alterations of SLC2A1 lead to its aberrant expression in tumors.

**FIGURE 4:**
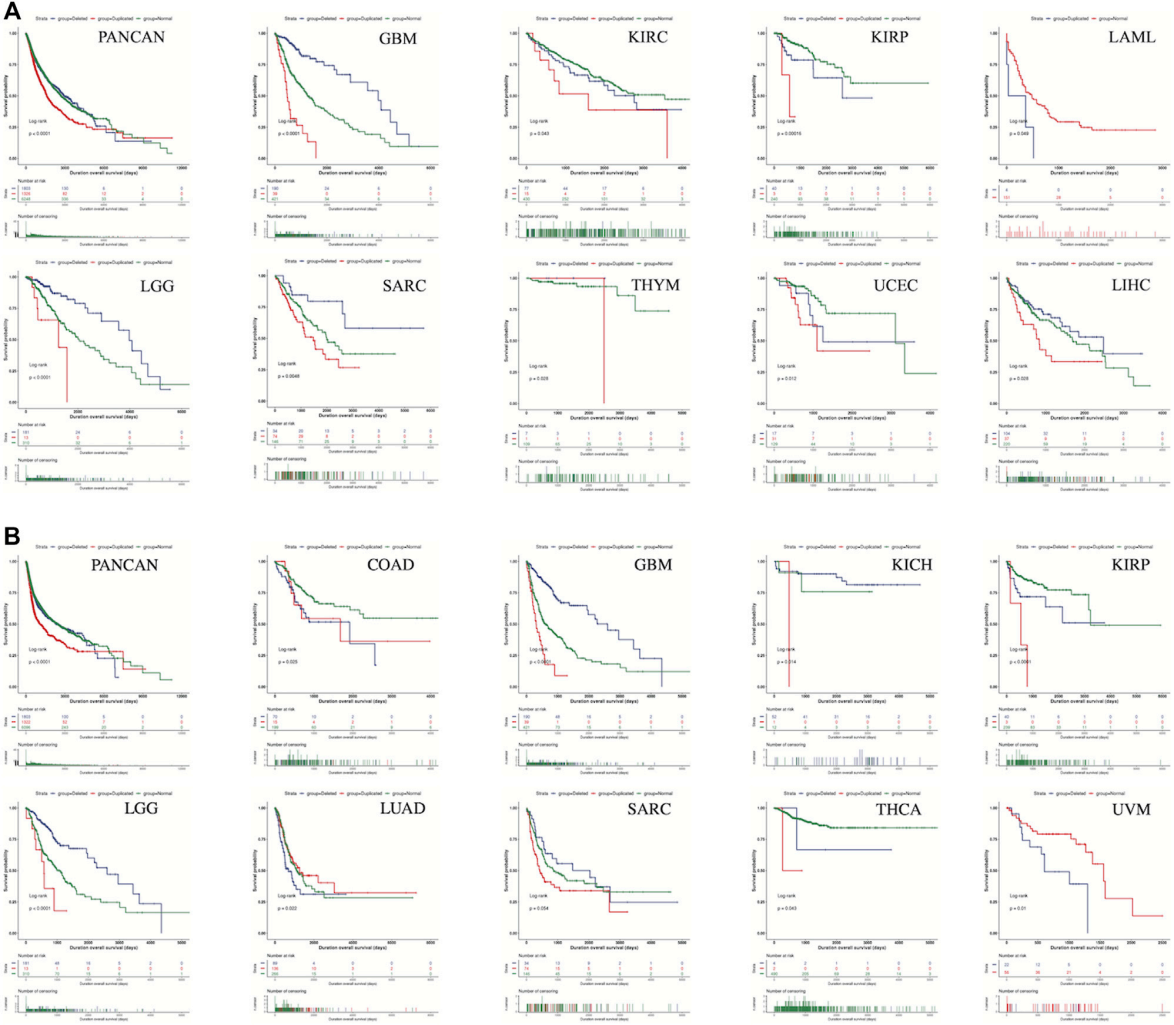
The CNA species of *SLC2A1* was strongly associated with tumor prognosis in a wide range of cancers **(A)** The CNA species of *SLC2A1* was strongly associated with OS in 10 cancers **(B)** The CNA species of *SLC2A1* was strongly associated with PFS in 10 cancers.

According to the Kaplan-Meier curves of the *SLC2A1*-altered group and -unaltered groups, there were no significant differences in survival (Supplementary Figure S3). The results of the analysis using UCSCXenaShiny, however, showed that the CNA species of *SLC2A1* was strongly associated with tumor prognosis in a wide range of cancers. The results showed that in PANCAN dataset, both OS (Figure 4A) and PFS (Figure 4B) in the *SLC2A1* normal group were intermediate between the *SLC2A1* deleted and duplicated groups. In contrast, *SLC2A1* duplicated alteration represented a poor prognosis in almost all statistically significant subgroups but was a surprise in LUAD. In LUAD, PFS was significantly higher in the *SLC2A1* duplicated group than in the *SLC2A1* normal and deleted groups (Figure 4B).

### 3.4 SLC2A1 was aberrated in most cancers at the protein expression level

**FIGURE 5:**
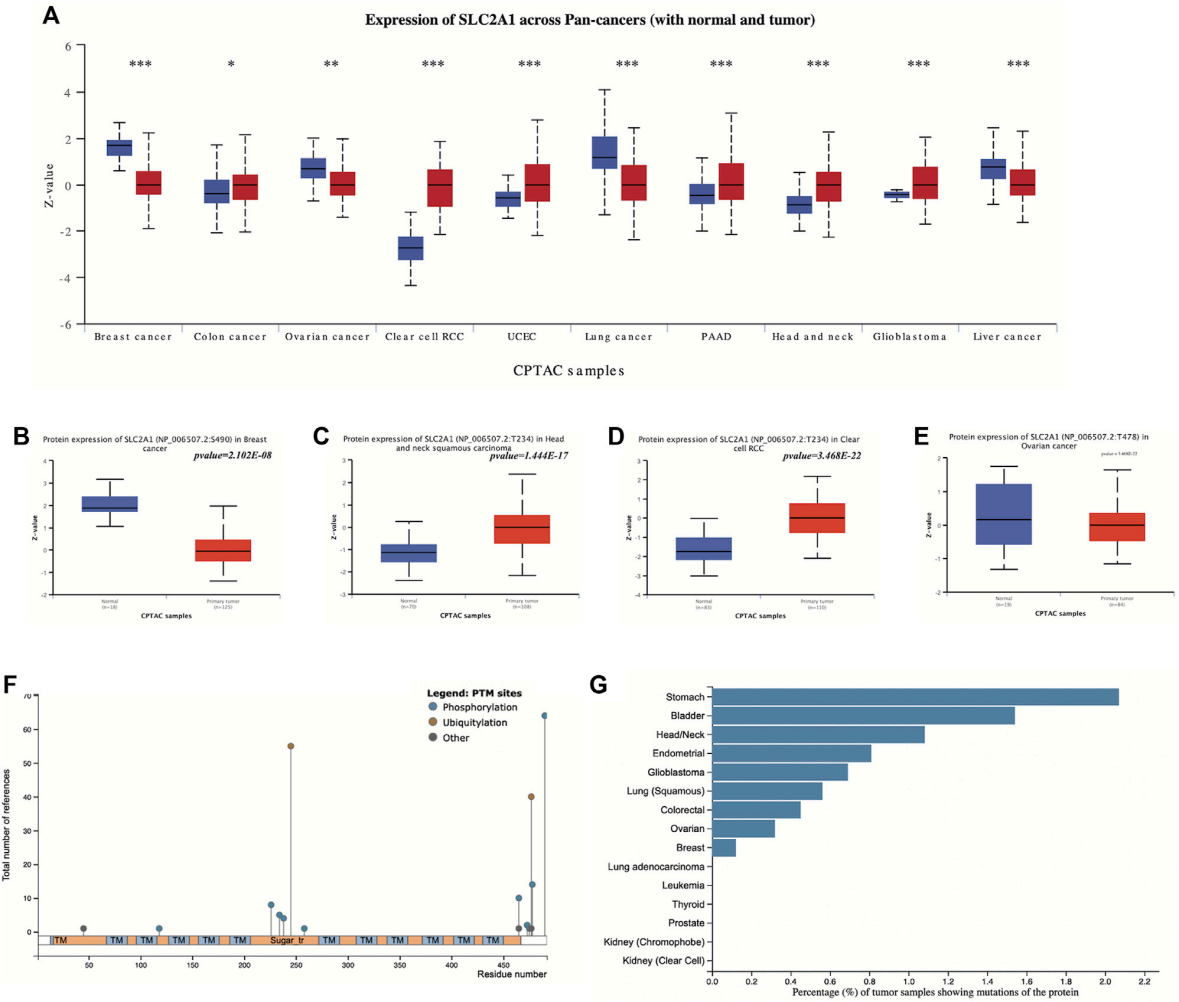
Proteomic evaluation of SLC2A1 in pan-cancer **(A)** The protein expression levels of SLC2A1 in normal and primary tumor tissues were compared in UALCAN using CPTAC data **(B–E)** Phosphorylation of SLC2A1 in tumor and normal tissues according to UALCAN **(F)** The type of SLC2A1 PTM sites and their number of references in research according to the PhosphpSitePlus database **(G)** Frequency of SLC2A1 protein alterations in different tumors according to the PhosphpSitePlus database. PTM, post-translational modification; *p < 0.05; **p < 0.01; ***p < 0.001.

The protein expression levels and phosphorylation of SLC2A1 in normal and primary tumor tissues were compared in UALCAN using CPTAC data. The protein expression of SLC2A1 was significantly decreased in BRCA, OV, LIHC, and lung cancer, while being increased in COAD, KIRC, UCEC, PAAD, HNSC, and GBM (Figure 5A).

We explored the differences in SLC2A1 phosphorylation in tumor and normal tissues. Among the data from four tumor types with SLC2A1 phosphorylation information included in CPTAC, we found a decreased S490 phosphorylation in BRCA, increased T234 phosphorylation in HNSC, and increased T234 phosphorylation in KIRC (Figures 5B–D). There were no significant changes in SLC2A1 phosphorylation in OV (Figure 5E).

To further explore the relationship between tumors and SLC2A1 alterations at the proteomic level, we analyzed SLC2A1 post-translational modification (PTM) sites and their frequency in different tumors in the PhosphpSitePlus database. The most frequent protein modifications of SLC2A1 are phosphorylation and ubiquitination (Figure 5F). Among all tumors, the frequency of SLC2A1 mutations was highest in gastric, bladder, and head and neck tumors (Figure 5G), which is strongly consistent with the results obtained with UALCAN data.

### 3.5 High expression of SLC2A1 predicted poor prognosis in a variety of cancers

**FIGURE 6:**
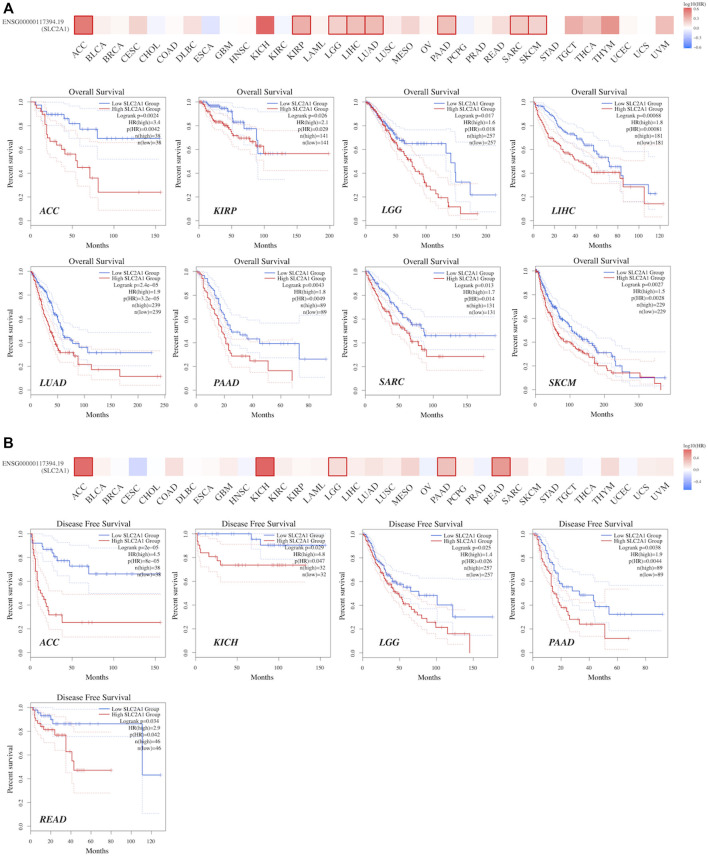
The prognostic values of SLC2A1, including OS and DFS, were evaluated across TCGA cancers in GEPIA2 **(A)**The expression of SLC2A1 was associated with OS in patients with ACC, KIRP, LGG, LIHC, LUAD, PAAD, SARC and SKCM **(B)** The expression of SLC2A1 was associated with DFS in patients with ACC, KICH, LGG, PAAD and READ. The cutoff value was set as follows: *p*-value < 0.05 and |logFC| > 1.

GEPIA2.0 was used to identify the prognostic significance of SLC2A1 expression in pan-cancer. As shown in Figure 6A, high SLC2A1 expression was associated with poor OS in patients with ACC (*p* = 0.0042), KIRP (*p* = 0.026), brain lower grade glioma (LGG) (*p* = 0.017), LIHC (*p* < 0.001), LUAD (*p* < 0.001), PAAD (*p* = 0.0049), SARC (*p* = 0.013), and SKCM (*p* = 0.0028). Furthermore, according to Figure 6B, high SLC2A1 expression was associated with poor DFS in patients with ACC (*p* < 0.001), KICH (*p* = 0.047), LGG (*p* = 0.026), PAAD (*p* = 0.0044), and READ (*p* = 0.042).

**FIGURE 7:**
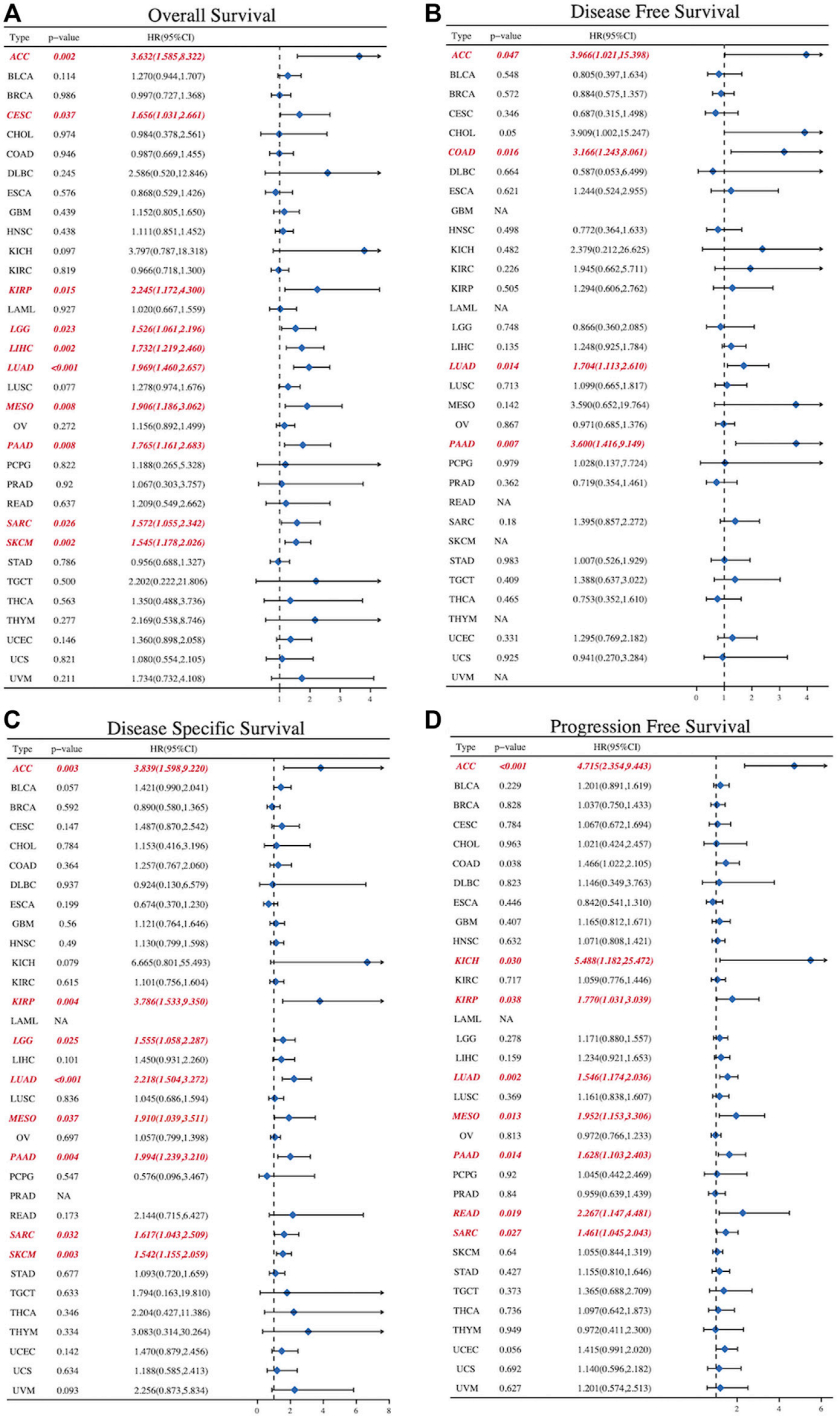
The forest diagrams of univariate Cox regression analyses in **(A)**OS **(B)** DFS **(C)** DSS, and **(D)** PFS. The red mark demonstrates that SLC2A1 expression was significantly associated with prognosis. That HR > 1 indicated that it served as a risk factor for survival. HR < 1 indicated that it had the protective effect on survival. OS, overall survival; DFS, disease free survival; DSS, disease specific survival; PFS, progression free survival; HR, hazard ratio; CI, confidence interval.

To further explore the role of SLC2A1 as a prognostic biomarker in cancer, we analyzed TCGA Pan-Cancer survival data, including OS, DFS, disease-specific survival (DSS), and progression-free survival (PFS), and presented the results in the form of a forest diagram. The univariate Cox regression analysis showed that SLC2A1 expression levels were strongly related to OS in 10 cancers, DFS in four cancers, DSS in eight cancers, and PFS in eight cancers (Figure 6A–6D). SLC2A1 expression levels were strongly related to OS in ACC (HR = 3.632, *p* = 0.004), CESC (HR = 1.656, *p* = 0.037), KIRP (HR = 2.245, *p* = .015), LGG (HR = 1.526, *p* = 0.023), LIHC (HR = 1.732, *p* = 0.002), LUAD (HR = 1.969, *p* < 0.001), MESO (HR = 1.906, *p* = 0.008), PAAD (HR = 1.765, *p* = 0.008), SARC (HR = 1.572, *p* = 0.026) and SKCM (HR = 1.545, *p* = 0.002) (Figure 7A). At the same time, SLC2A1 was a risk factor in these cancers. DFS analysis results showed that SLC2A1 was a risk factor in ACC (HR = 3.966, *p* = 0.047), COAD (HR = 3.166, *p* = 0.016), LUAD (HR = 1.704, *p* = 0.014) and PAAD (HR = 3.600, *p* = 0.007) (Figure 7B). PFS analysis results revealed that SLC2A1 was a risk factor in ACC (HR = 3.839, *p* = 0.003), KIRP (HR = 3.786, *p* = 0.004), LGG (HR = 1.555, *p* = 0.025), LUAD (HR = 2.218, *p* < 0.001) MESO (HR = 1.910, *p* = 0.037), PAAD (HR = 1.994, *p* = 0.004), SARC (HR = 1.617, *p* = 0.032) and SKCM (HR = 1.542, *p* = 0.003) (Figure 7C). DSS results indicated that SLC2A1 was a risk factor in ACC (HR = 4.715, *p* < 0.001), KICH (HR = 5.488, *p* = 0.030), KIRP (HR = 1.770, *p* = 0.038), LUAD (HR = 1.546, *p* = 0.002), MESO (HR = 1.952, *p* = 0.013), PAAD (HR = 1.628, *p* = 0.014), READ (HR = 2.267, *p* = 0.019) and SARC (HR = 1.461, *p* = 0.027) (Figure 7D). These results indicate that SLC2A1 expression is significantly associated with the prognosis of many cancers and will negatively affect cancer prognosis.

### 3.6 The expression of SLC2A1 was closely related to tumor immune microenvironment and tumor immunotherapy

**FIGURE 8:**
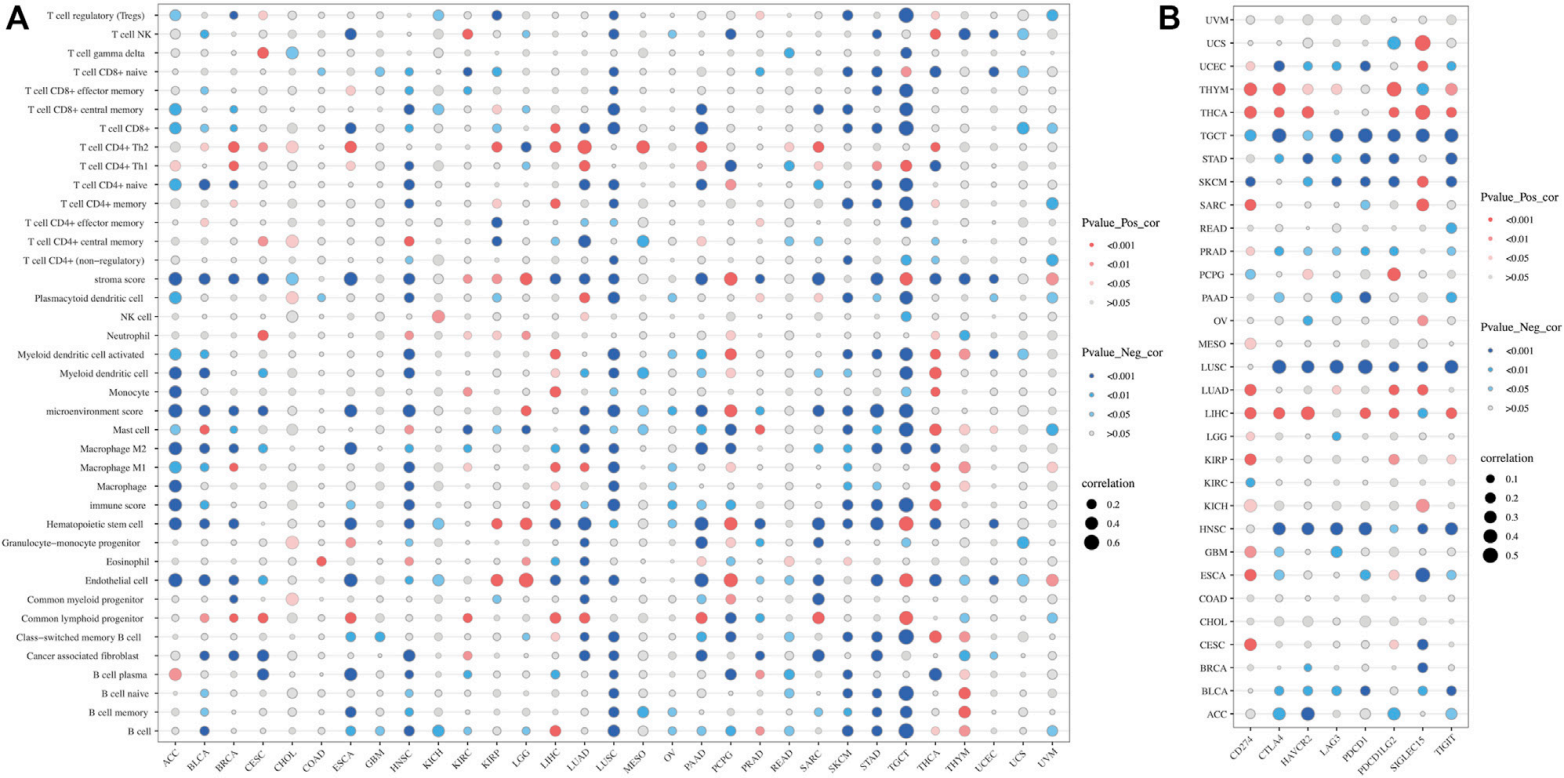
Correlation of SLC2A1 expression with **(A)** immune infiltrating cells in pan-cancer by CIBERSORT algorithm **(B)** Immune checkpoint genes, including CD274, CTLA4, HAVCR2, LAG3, PDCD1, PDCD1LG2, SIGLEC15, and TIGIT. Red indicated a positive correlation and blue indicated a negative correlation, and the size of the bubble indicates the magnitude of the correlation.

In recent years, immune cell infiltration in the tumor microenvironment (TME) has been found to have a close influence on tumorigenesis, progression, and other behaviors. Therefore, we explored the potential correlation between SLC2A1 expression and tumor-infiltrating immune cells by performing comprehensive research. Firstly, we used the XCELL algorithm to score the immune infiltration of all TCGA Pan-Cancer samples and calculated the correlation between SLC2A1 expression and 36 different immune infiltrating cells and tumor microenvironment scores in each cancer (Figure 8A). There was a strong correlation between SLC2A1 expression and stroma score, microenvironment score and immune score in most cancers. Additionally, SLC2A1 was strongly correlated with most immune cell infiltration in ACC, HNSC, LUSC, PAAD, STAD and TGCT. Detailed correlation analysis results are provided in Supplementary Table S1.

**FIGURE 9:**
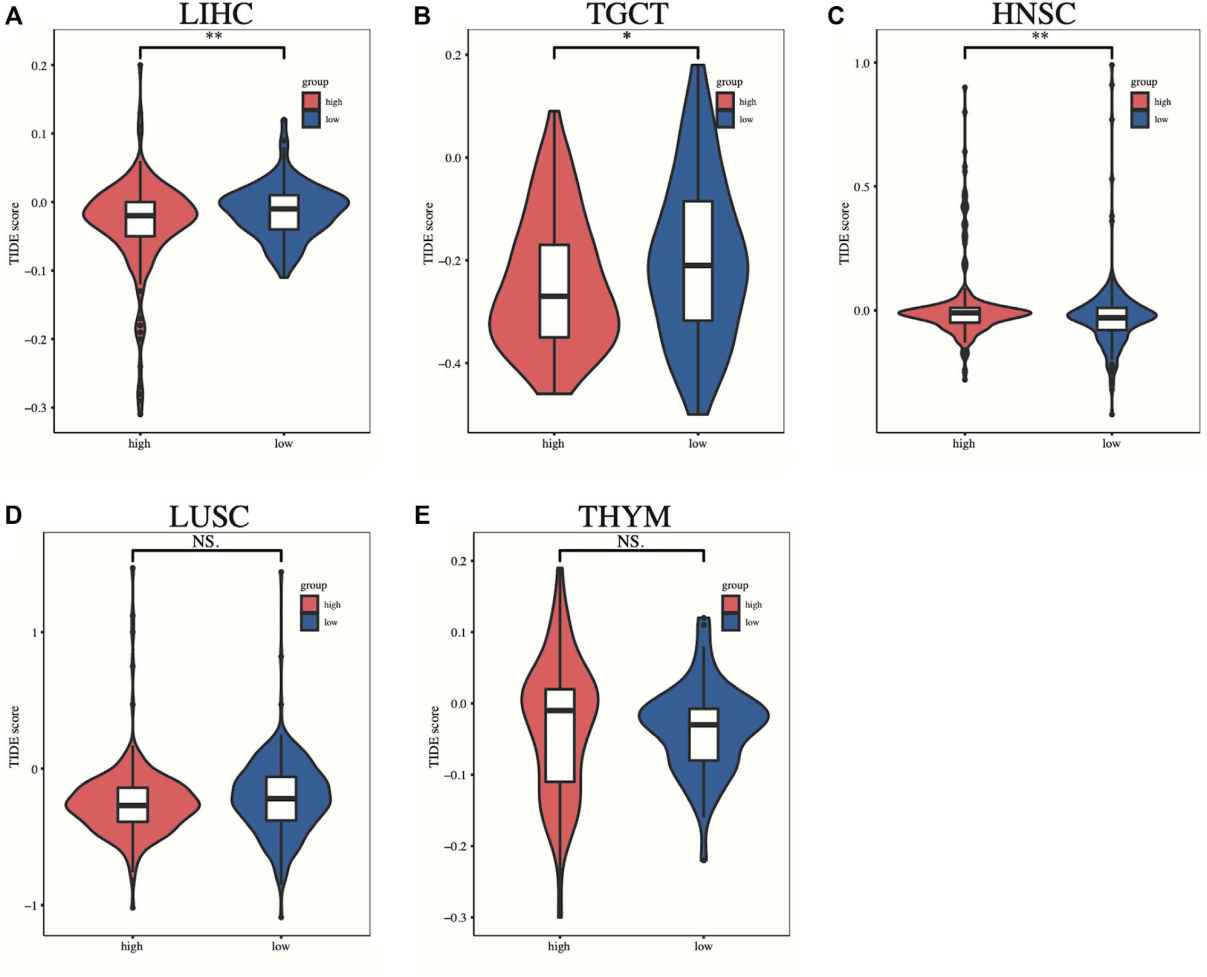
The difference in TIDE scores between the high and low SLC2A1 expression groups in **(A)** LIHC **(B)** TGCT **(C)** HNSC **(D)** LUSC **(E)** UVM. TIDE, tumor immune dysfunction and exclusion. *p < 0.05; **p < 0.01; ***p < 0.001.

To investigate the relationship between SLC2A1 expression and immunotherapy, we extracted eight immune checkpoint-related genes (Ravi et al., 2018; Wang et al., 2019; Zeng et al., 2019; Yi et al., 2020) and evaluated their association with SLC2A1. In some cancers, such as LIHC and THYM, we found that SLC2A1 expression was positively correlated with immune checkpoint genes, while being negatively correlated in HNSC, LUSC, and TGCT (Figure 8B). Detailed correlation analysis results are provided in Supplementary Table S1. According to this, we calculated the difference in TIDE scores between the high and low SLC2A1 expression groups to explore the potential immune checkpoint blocker response of SLC2A1 on immunotherapy in these cancers. Higher TIDE scores presented worse ICB treatment results. In LIHC and TGCT, the high SLC2A1 expression group had lower TIDE scores, i.e., patients with high SLC2A1 expression may have better ICB treatment outcomes (Figures 9A, B). In contrast, in HNSC, the high SLC2A1 expression group had higher TIDE scores, indicating a worse ICB treatment outcome (Figure 9C). In LUSC and THYM, TIDE scores were not significantly different in the high and low SLC2A1 expression groups (Figure 9D, E). To further validate our conclusions, we performed a similar analysis in a public database.

**FIGURE 10:**
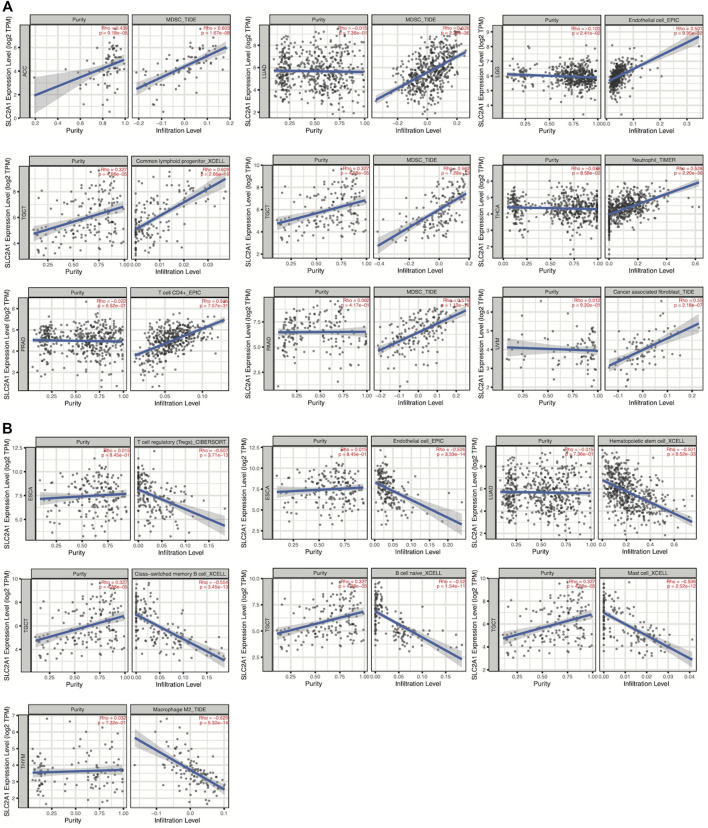
Spearman’s correlation analysis showed the **(A)** positive and **(B)** negative association between SLC2A1 expression level and immune infiltration cells across different TCGA cancer types according to TIMER2.0. TPM, Transcript per million.

We first performed an immune infiltration cell-related assessment of SLC2A1 in TIMER2.0 and identified results with correlation coefficients of absolute values greater than 0.5. As shown in the results, we found a significant positive association between SLC2A1 expression and the immune infiltration value of myeloid-derived suppressor cells (MDSCs) in ACC (r = 0.603), LUAD (r = 0.526), TGCT (r = 0.562) and PAAD (r = 0.564); endothelial cells in LGG (r = 0.501), common lymphoid progenitors in TGCT (r = 0.609); neutrophils in THCA (r = 0.528); CD4^+^ T cells in PRAD (r = 0.525) and cancer-associated fibroblast in UVM (r = 0.550) (Figure 10A). We identified a negative correlation between SLC2A1 expression and the immune infiltration value of regulatory T cells (Tregs) (r = −0.507) and endothelial cells (r = −0.526) in ESCA; hematopoietic stem cells in LUAD (r = −0.501); class-switched memory B cells (r = −0.554), naïve B cells (r = −0.52), and mast cells (r = −0.536) in TGCT; and M2 macrophages in THYM (r = −0.629) (Figure 10B).

**FIGURE 11:**
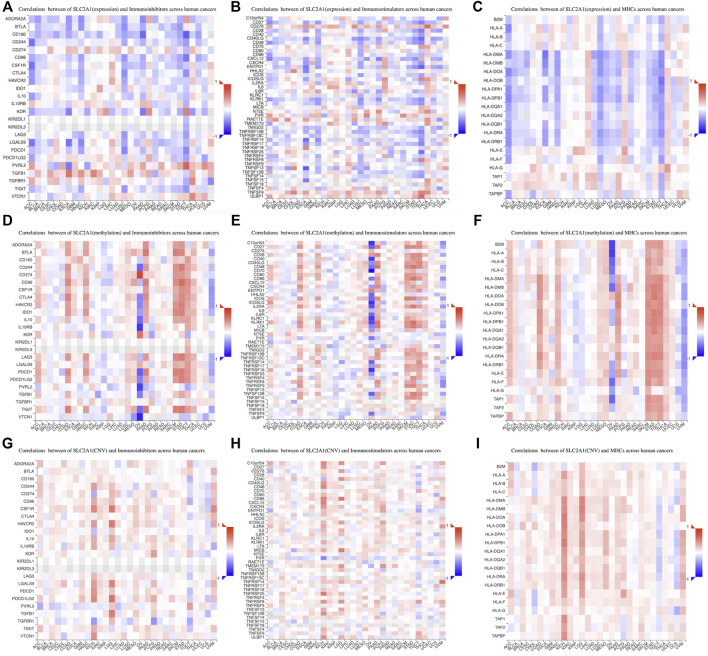
The association between SLC2A1 expression and **(A)** immunoinhibitors genes **(B)** immunostimulators genes, and **(C)** MHC genes in pan-cancer according to TISIDB database. The association between SLC2A1 methylation level and **(D)** immunoinhibitors genes **(E)** immunostimulators genes, and **(F)** MHC genes in pan-cancer. The association between copy number variant level of SLC2A1 and **(G)** immunoinhibitors genes **(H)** immunostimulators genes, and **(I)** MHC genes in pan-cancer. Red indicated a positive correlation and blue indicated a negative correlation.

We then investigated the relations between three kinds of immunomodulators and *SLC2A1* expression. *SLC2A1* expression correlated with multiple immunoinhibitory genes in most tumors, such as *CD244*, *CSF1R*, and *TGFB1* (Figure 11A); immunostimulatory genes, such as *TNFSF13* and *TNFSF15* (Figure 11B); and MHC genes, such as *CD40LG* and *TNFSF9* (Figure 11C). Additionally, we also analyzed the relationship between the methylation and CNV levels of *SLC2A1* and these genes. The methylation level of *SLC2A1* was closely associated with these genes in OV, PAAD, SKCM, and STAD (Figure 11D–F). The CNV level of *SLC2A1* was closely associated with these genes in KICH and LGG (Figure 11G–I). The specific results of correlation coefficients with absolute values greater than 0.5 are shown in Supplementary Figures S4–S6.

### 3.7 *SLC2A1* expression was closely related to biological functional status in cancer at the single-cell level

**FIGURE 12:**
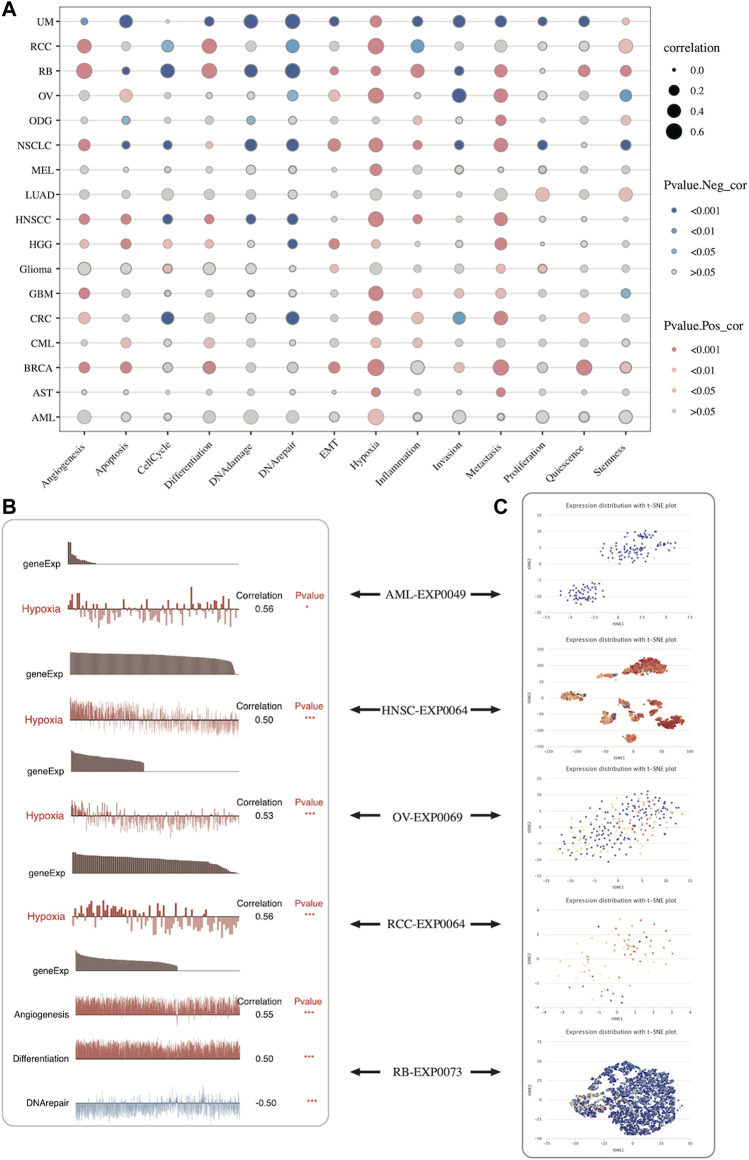
Expression pattern of SLC2A1 at the single-cell level and its relationship with the tumor-related biological functional status according to CancerSEA database **(A)** The correlation between SLC2A1 expression and different tumor functional status across pan-cancer **(B)** The correlation between SLC2A1 expression and different tumor functional status in individual datasets **(C)** SLC2A1 expression distribution of AML-EXP0049, HNSC-EXP0064, OV-EXP0069, KIRC-EXP0064 and RB-EXP0073 were shown at single-cell levels by t-SNE diagram.

The CancerSEA database was applied to explore the relationship between *SLC2A1* expression and biological functions commonly involved in tumor occurrence and development in pan-cancer at the single-cell level. *SLC2A1* expression was associated with common tumor biological function in UVM, KIRC, and retinoblastoma (RB) to varying degrees. In most cancers, SLC2A1 was highly related to hypoxia (Figure 12A; Supplementary Table S3).

Figure 12A shows the results of a comprehensive analysis of all single-cell datasets included in CancerSEA. Additionally, we explored the correlation of SLC2A1 in individual datasets with various functions. The results showed that SLC2A1 showed a strong correlation with hypoxia in AML-EXP0049, HNSC-EXP0064, OV-EXP0069, and KIRC-EXP0064 single-cell datasets, with angiogenesis, differentiation, and DNA repair in the RB-EXP0073 single-cell dataset (Figure 12B). Additionally, *SLC2A1* expression distribution within these datasets was shown at the single-cell level by the t-SNE diagram (Figure 12C). From what has been discussed above, we found that *SLC2A1* expression is closely related to biological functional status in cancer.

### 3.8 SLC2A1 was closely associated with programmed cell death, hypoxia and metabolism

**FIGURE 13:**
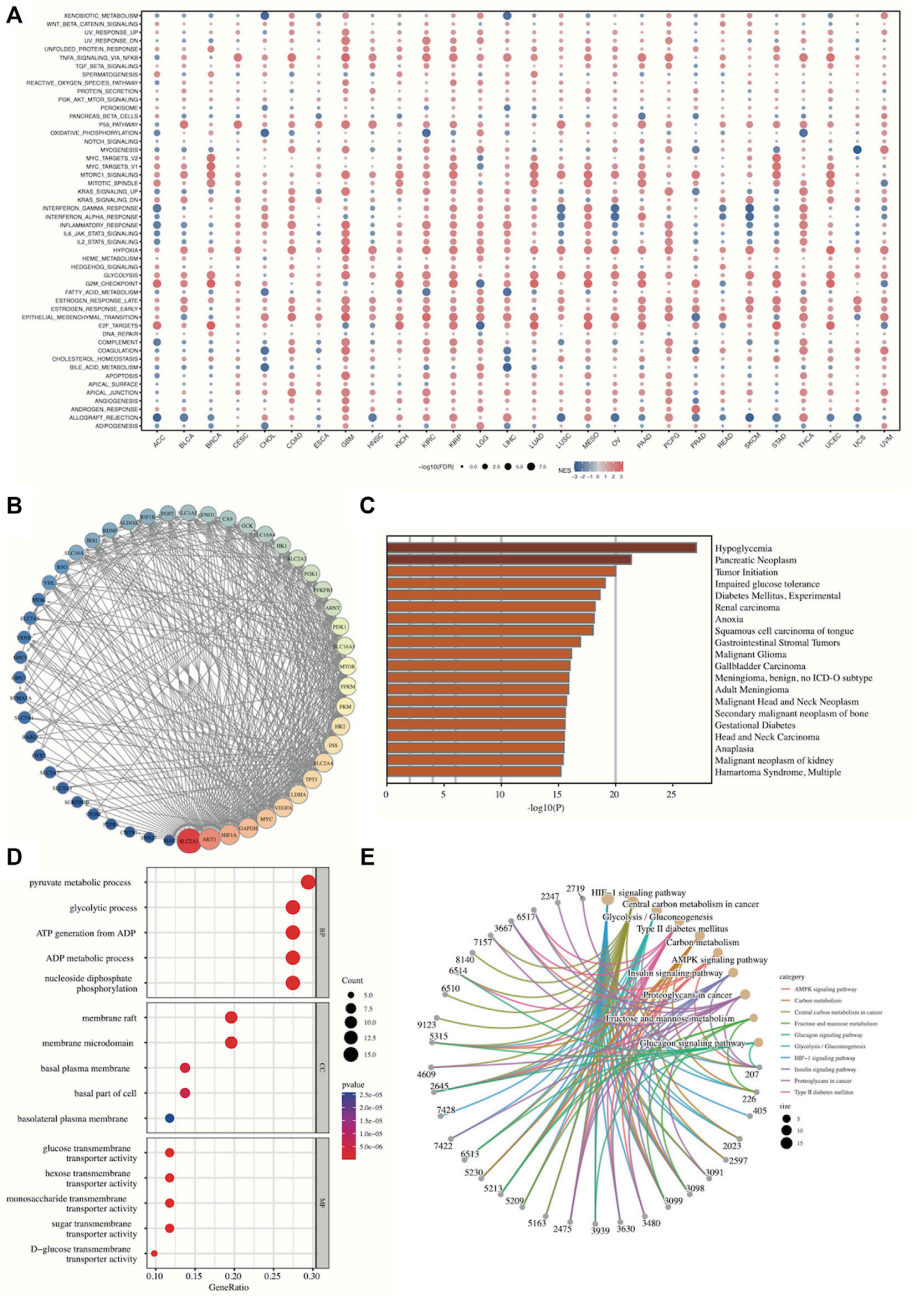
Gene Set Enrichment Analysis and protein-protein interaction network of SLC2A1 **(A)** The correlation analysis between SLC2A1 and the enrichment scores of 50 HALLMARK pathways across TCGA cancers **(B)** Construction of PPI network involved in 51 SLC2A1-interacting proteins based on STRING and adjusted by Cytoscape **(C)** The disease specific enrichment analysis for SLC2A1-related genes by Metascape **(D)** The GO enrichment analysis for SLC2A1-related genes contained in PPI network **(E)** The KEGG enrichment analysis for SLC2A1-related genes contained in PPI network. FDR, false discovery ratio; NES, normalized enrichment score.

Finally, we further explored the biological function of SLC2A1 by functional enrichment of *SLC2A1*-related genes and functional interaction network construction. The correlation analysis between SLC2A1 and the enrichment scores of 50 HALLMARK pathways across TCGA cancers (Figure 13A) showed that SLC2A1 was significantly positively correlated with MTORC1_SIGNALING, HYPOXIA, EPITHELIAL_MESENCHYMAL_TRANSITION, and ALLOGRAFT_REJECTION in almost all cancers. Additionally, SLC2A1 expression was associated with almost all pathways in GBM, KIRC, and KIRP. Detailed results of the correlation analysis are provided in Supplementary Table S4.

A 51-protein network centered on SLC2A1 was constructed by STRING and adjusted by Cytoscape (Figure 13B). We then conducted disease-specific enrichment analysis to explore the diseases correlating to these genes in Metascape. *SLC2A1*-related genes were mainly related to hypoglycemia, pancreatic neoplasm, tumor initiation, and many other tumors, among others (Figure 13C). GO and KEGG enrichment analysis for the 51 genes contained in the PPI network were conducted (Figure 11D, E; Supplementary Tables S5, S6). These *SLC2A1*-related genes were significantly associated with many metabolism-related biological functions, such as pyruvate metabolic processes, ADP metabolic processes, and central carbon metabolism in cancer pathways. Furthermore, they were also associated with HIF-1 signaling, which is related to hypoxia.

**FIGURE 14:**
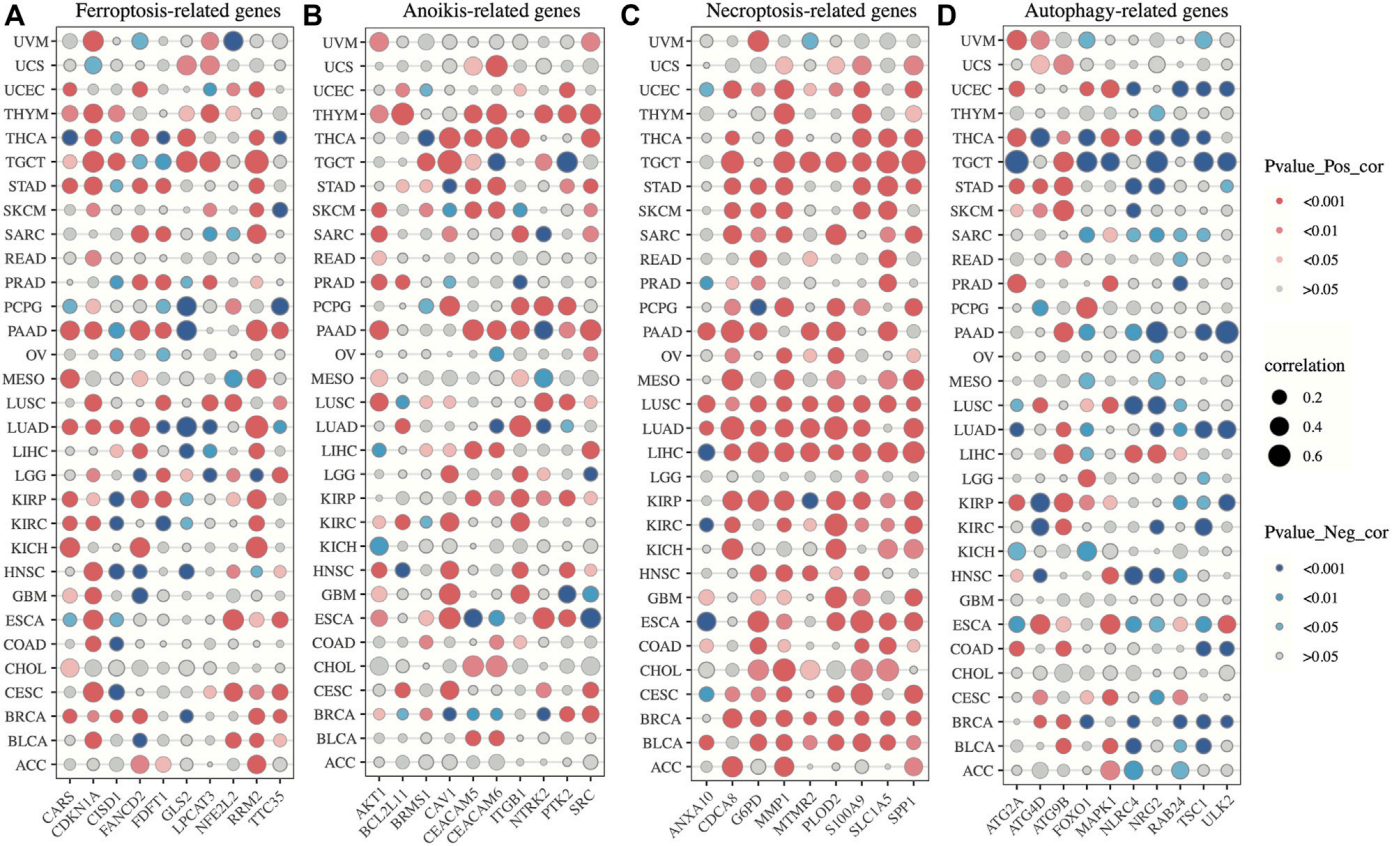
Correlation analysis between SLC2A1 and programmed cell death-related genes **(A)** SLC2A1 was correlated with ferroptosis-related genes in pan-cancer **(B)** SLC2A1 was correlated with anoikis-related genes in pan-cancer **(C)** SLC2A1 was correlated with necroptosis-related genes in pan-cancer **(D)** SLC2A1 was correlated with autophagy-related genes in pan-cancer.

Additionally, according to the results of correlation analysis between SLC2A1 and programmed cell death-related genes, SLC2A1 was closely associated with these genes in almost all tumors, especially with Necroptosis-related genes, and its correlation reached above .6 in many tumors (Figure 14A–D; Supplementary Table 6). The results of these analyses further emphasize the role of SLC2A1 as a multiple programmed cell death-associated gene.

## 4 Discussion

Several studies have pointed out the potential of the SLC transporter family in drug discovery (Lin et al., 2015; Rives et al., 2017; Scalise et al., 2019; Schumann et al., 2020), and *SLC2A1* has been included as a key gene for diagnostic or prognostic cancer signature prediction in several cancers (Mo et al., 2020; Chen et al., 2021a; Qin et al., 2021; Chen et al., 2022). However, no pan-cancer analysis of *SLC2A1* had been available thus far. Our study provides a comprehensive and systematic analysis of the role of *SLC2A1* in human cancers, investigates its differences and commonalities among different cancer species, and explores its potential as a pan-cancer biomarker.

First, the analysis of *SLC2A1* expression in cancer was performed. Combining the results of unpaired and paired expression difference analysis, *SLC2A1* was found to be differentially expressed in almost all tumors, especially in BRCA, CESC, COAD, HNSC, KIRC, LUAD, LUSC, READ, STAD, and UCEC. In contrast, *SLC2A1* showed high expression in most cancers and low expression only in LAML and SKCM. *SLC2A1* expression was correlated with clinical staging in 11 cancers, which indicated that *SLC2A1* was associated with tumor progression. It was also observed that *SLC2A1* expression in these tumors was positively correlated with the TNM stage of most tumors, i.e., as the stage increased, *SLC2A1* expression increased, which was consistent with the results of the analysis of expression differences in tumors and normal tissues. However, in ESCA and OV, SLC2A1 showed a decrease in expression with increasing tumor stage, which appeared contradictory to the results of our analysis and may require further study.

After that, we analyzed *SLC2A1* expression at the epigenetic level. *SLC2A1* promoter DNA methylation levels were abnormal in 10 cancers. Most of these tumors with abnormal *SLC2A1* promoter methylation levels also showed abnormal SLC2A1 mRNA expression levels, which may indicate that promoter methylation plays a role in aberrant *SLC2A1* expression. This was confirmed by the results of further correlation analysis. Recently, Zou et al. found that *SLC2A1* methylation and transcript levels are dramatically elevated when induced by environmental factors, inhibiting ferroptosis and autophagy and leading to reduced immune system function and, thus, poor prognosis in patients (Zou et al., 2022). Wang et al. also observed that methylation and aberrant expression of *SLC2A1* lead to poor prognosis in lung adenocarcinoma (Wang et al., 2020). Nevertheless, the abnormal expression of SLC2A1 in some cancers still could not be explained, such as LIHC, LUAD, THCA.

We then explored *SLC2A1* in terms of gene mutation and copy number variation. Genetic alterations in *SLC2A1* were present in most cancers, and there were 80 mutation sites in *SLC2A1*, most of which were missense mutations. Although the type of genetic alteration in *SLC2A1* was not significantly associated with SLC2A1 expression, correlation analysis of CNV levels of *SLC2A1* and SLC2A1 expression levels showed a significant association between the two in 19 cancers. This suggested that for the abnormal expression of SLC2A1 that cannot be explained by epigenetic modifications, the cause might be due to genetic alterations of the gene.

Although there was no association between the genetic alteration type of *SLC2A1* and SLC2A1 expression, the type of genetic alteration in *SLC2A1* was strongly associated with tumor prognosis. In survival analyses of the *SLC2A1* normal, duplicated, and deleted groups, the *SLC2A1* duplicated group showed poor prognostic significance in several cancer types. In particular, in GBM, KIRP, LGG, SARC, the CNA species of *SLC2A1* was strongly correlated with both OS and PFS. More importantly, this variability is still significant in the PANCAN dataset. This suggests that the type of CNA alteration in *SLC2A1* can be used as a generalized tumor prognostic predictor. In LUAD, however, PFS was higher in the *SLC2A1* duplicated group than in the normal and deleted groups, in contrast to the results of analyses in other cancer types. In fact, there have been studies using genetic CNV status to predict tumor prognosis. Wang et al. found that CNV of METTL4 could be a prognostic biomarker for soft tissue sarcomas (STS) by potentially influencing mast cell infiltration and DNA methylation (Mo et al., 2021). Hu et al. constructed a CNV-based prognostic signature for breast cancer that showed good prediction (Zhang et al., 2021). However, the mechanisms underlying the relationship between the CNV status of SLC2A1 and tumor prognosis are still unclear, and the paradoxical results exhibited in LUAD in particular deserve further investigation. This certainly provides new ideas and directions for subsequent studies.

We also analyzed the relationship between SLC2A1 and tumors at the proteomic level. The protein expression levels of SLC2A1 showed abnormalities in all 10 cancers in the protein expression data provided by CPTAC. In BRCA, NSCLC, and LIHC, *SLC2A1* protein expression was significantly reduced relative to that in normal tissues, which contrasted with its transcriptome expression levels. This suggests that the translational and post-translational modification processes of SLC2A1 may be abnormal in these cancers. Sure enough, we found that the phosphorylation level of SLC2A1 was significantly reduced in BRCA. In addition, phosphorylation and ubiquitination of SLC2A1 were altered in a variety of cancers, predominantly in gastric, bladder, and head and neck tumors. This is consistent with the results of many published studies (Fenske et al., 2009; Bonfitto et al., 2010; Shen et al., 2020; Chen et al., 2021b). Mo et al. found that upregulated SUMO2 promotes SLC2A1 degradation through sumo- and ubiquitination of SLC2A1, which leads to the proliferation and metastasis of nasopharyngeal carcinoma (Chen et al., 2021b). Zhang et al. found that DHHC9-mediated GLUT1 S-palmitoylation promotes glioblastoma glycolysis and tumorigenesis (Shen et al., 2020).

Our investigation of prognosis yielded surprising results. Studies have shown that high SLC2A1 expression is suggestive of poor prognosis in most cancers. SLC2A1 was significantly associated with OS and DSS in ACC, KIRP, LGG, LUAD, MESO, PAAD, SARC, and SKCM. This indicates that SLC2A1 is valuable in these cancers both as a long-term and short-term evaluation index. Especially in ACC, the HR of SLC2A1 in the univariate regression analysis with OS, PFS, DSS, and DFS as endpoints was more than 3.5. However, there is only one study from 2009 that confirms the prognostic predictive function of SLC2A1 in ACC (Krawczyk et al., 2021). In another study conducted by Bonfitto et al., it was stated that SLC2A1 did not have a prognostic predictive role, despite its high expression in ACC (Guo et al., 2020). Therefore, more relevant studies may be needed to investigate the relevant role and mechanism of SLC2A1 in ACC. There are also many studies (Schreiber et al., 2011; Tesi, 2019; Wang et al., 2022) confirming the prognostic predictive role of SLC2A1 in some tumors, but they are mostly bioinformatic analyses and more experiments are needed to validate the above analyses. Overall, however, these results suggest that SLC2A1 has the potential to be a pan-cancer prognostic biomarker.

The inextricable link between tumor immunity and tumor prognosis is now well-established (Charoentong et al., 2017). Many genes affect tumor progression by altering the TME and influencing the degree of tumor immune cell infiltration. Therefore, we further explored the link between SLC2A1 and tumor immunity. The results of the tumor immune cell infiltration analysis showed that SLC2A1 was significantly associated with multiple immune cell infiltrations in a variety of cancers. For example, SLC2A1 was significantly postively correlated with Th2 CD4^+^ T cells and significantly negtively correlated with CD 8 + T cell, M2 macrophages and B cell in a variety of cancers. High expression of SLC2A1 can affect the prognosis of gastric cancer by suppressing CD8^+^ T cells and B cells (Min et al., 2021). Additionally, we found that SLC2A1 was significantly associated with MDSC infiltration in ACC, LUAD, TGCT, and PAAD. MDSC protects tumors from immune system attack and renders them resistant to immunotherapy (Lei et al., 2021a). Therefore, high MDSC infiltration levels are highly likely to lead to poor prognosis. The above studies show that SLC2A1 has a regulatory role in the degree of infiltration of multiple immune cells including MDSC, B cells, and Tregs in a variety of tumors, thus affecting tumor progression and prognosis.

The efficacy of immune checkpoint inhibitors (ICB) in treating tumors is widely recognized, and immune checkpoint-related genes are currently of interest to investigators. We identified eight immune checkpoint-related genes from the literature and analyzed their correlation with SLC2A1. SLC2A1 was significantly negatively correlated with these eight genes in TGCT, LUSC, and HNSC, while in THYM and LIHC, SLC2A1 was significantly positively correlated with them. This suggests that aberrant SLC2A1 expression in these cancers most likely influences the expression profile of immune checkpoints and thus alters the therapeutic effect of ICB. TIDE uses a set of gene expression markers to assess two different mechanisms of tumor immune escape, including dysfunction of tumor-infiltrating cytotoxic T lymphocytes (CTL) and rejection of CTL by immunosuppressive factors. High TIDE scores were associated with poor ICB therapy efficacy and short survival (Jiang et al., 2018). The TIDE scores of the high SLC2A1 expression group were significantly higher than those of the low expression group in HNSC, while it showed opposite results in LIHC and TGCT. This suggests that the high SLC2A1 expression group in LIHC and TGCT may predict a better ICB outcome. However, there is still a gap in studies related to ICB in these three cancers, and more studies may be needed to validate our results.

To further explore the relationship between SLC2A1 and tumor immunity, we analyzed the correlation between SLC2A1 and immune-related markers on TISIDB. These immunomodulators, including immunostimulatory, immunoinhibitory, and MHC genes, were collected from Charoentong’s study (Jing et al., 2019). SLC2A1 was significantly associated with multiple immune-related markers in ESCA and TGCT. Reviewing the results of immune infiltration-related studies, we found that SLC2A1 was associated with multiple immune cell infiltrates such as epithelial cells, B cells, and Tregs in ESCA and TGCT. Although there are no immunotherapeutic agents for ESCA and TGCT, our findings clearly elucidate avenues of research and motivate their application in immunotherapy. In addition to mRNA expression, we also analyzed the correlation between SLC2A1 methylation levels, CNV levels, and these markers. The methylation level of SLC2A1 had a strong correlation in OV, PAAD, SKCM, and STAD. However, due to the small number of OV samples (9 samples) included in the study, a bias may be at play. In the other three cancers, although there are no relevant studies yet, this result is revealing and suggests that we may be able to explore the factors affecting tumor immune response from the perspective of *SLC2A1* promoter methylation. Also, CNV levels of SLC2A1 were significantly correlated with immune-related markers in KICH and LGG, which provides warrants further research.

Traditional bulk assays can only explain differences between samples from a holistic perspective, and their resolution is not sufficient to depict differences between individual cells. Single-cell RNA-seq can capture rare cell types while depicting the complex structure of TME most accurately and has become a powerful tool to study cellular heterogeneity (Mo et al., 2020). Therefore, we assessed the relevance of SLC2A1 to tumor biological function at the single-cell level using the CancerSEA database. In UM, SLC2A1 was negatively related to apoptosis, DNA damage, DNA repair, metastasis, and invasion, which showed that SLC2A1 might play an inhibitory role in tumorigenesis, progression, and metastasis. In RB, SLC2A1 was positively correlated with angiogenesis, differentiation, inflammation, and metastasis, while negatively correlated with the cell cycle and DNA damage and repair, which indicates that SLC2A1 might play a role in promoting the progression and metastasis of RB. Furthermore, we found a strong positive correlation between SLC2A1 and hypoxia in most cancers. It is well-known that hypoxia can lead to poor prognosis by modulating the TME. It can render tumors resistant to conventional therapies through multiple signaling pathways, such as apoptosis, autophagy, DNA damage, mitochondrial activity, p53, and drug efflux (Szwed et al., 2021). Mo et al. (2020) established a hypoxia-associated gene signature incorporating *SLC2A1* with a good prognostic prediction for lung adenocarcinoma (AUC = 0.715). This suggests that in some tumors, SLC2A1 can affect the TME by regulating hypoxia, thereby affecting prognosis.

To comprehensively explore SLC2A1 function in cancer, we subsequently performed GSEA and constructed protein interaction networks. Among the 50 HALLMARK pathways associated with cancer, SLC2A1 was significantly associated with MTORC1_SIGNALING, HYPOXIA, and EPITHELIAL_MESENCHYMAL_TRANSITION in most cancers. mTORC is an important target for cancer, aging, and metabolism-related diseases that consists of two complexes, mTORC1 and mTORC2 (Han and Wang, 2018). mTORC1 plays an important role in various biological functions such as autophagy, iron death, and lipid metabolism (Rabanal-Ruiz et al., 2017; Han and Wang, 2018; Mittal, 2018; Lei et al., 2021b). Epithelial-mesenchymal transition (EMT) can pathologically promote cancer development and metastasis and is closely related to cell development and stem cell properties [69]. The PPI network constructed based on STRING contained 51 genes, and GO, KEGG, and disease enrichment analyses were performed based on these genes. From the disease enrichment analysis, SLC2A1-related genes were closely associated with a variety of tumors. The GO and KEGG results suggest that these genes are associated with multiple glucose metabolism pathways in addition to some tumor-related pathways. Both GSEA and KEGG enrichment analyses suggested a strong correlation between SLC2A1 and hypoxia.

Nevertheless, our study has some limitations. Firstly, it was based on an online database and did not use our own collected and obtained data. However, we used a cross-validation method of multiple databases to make the results of our analysis as reliable as possible. Secondly, our results are based entirely on bioinformatics analysis, and many of them have not been experimentally validated yet. In terms of functional enrichment, we only showed the association of SLC2A1 with a given pathway and did not explore its specific regulatory mechanism within the pathway. However, there are few published studies in this area, and our study mainly provides new research directions. In the near future, we will validate our results through *in vivo* and *in vitro* experiments.

In conclusion, we have performed a systematic and comprehensive analysis of the role of SLC2A1 in pan-cancer from multiple perspectives, including expression, prognosis, immunity, and biological function. Our results revealed the potential of SLC2A1 to be developed as a pan-cancer prognostic marker and immunotherapy evaluation marker.

## Data Availability

The original contributions presented in the study are included in the article/Supplementary Material, further inquiries can be directed to the corresponding author.
