# Supplementary figures and images for: Cell death-related biomarker SLC2A1 has a significant role in prognosis prediction and immunotherapy efficacy evaluation in pan-cancer
Source: Front Genet. 2023 Jan 11;13:1068462. doi: 10.3389/fgene.2022.1068462 (PMC9873976; doi:10.3389/fgene.2022.1068462)

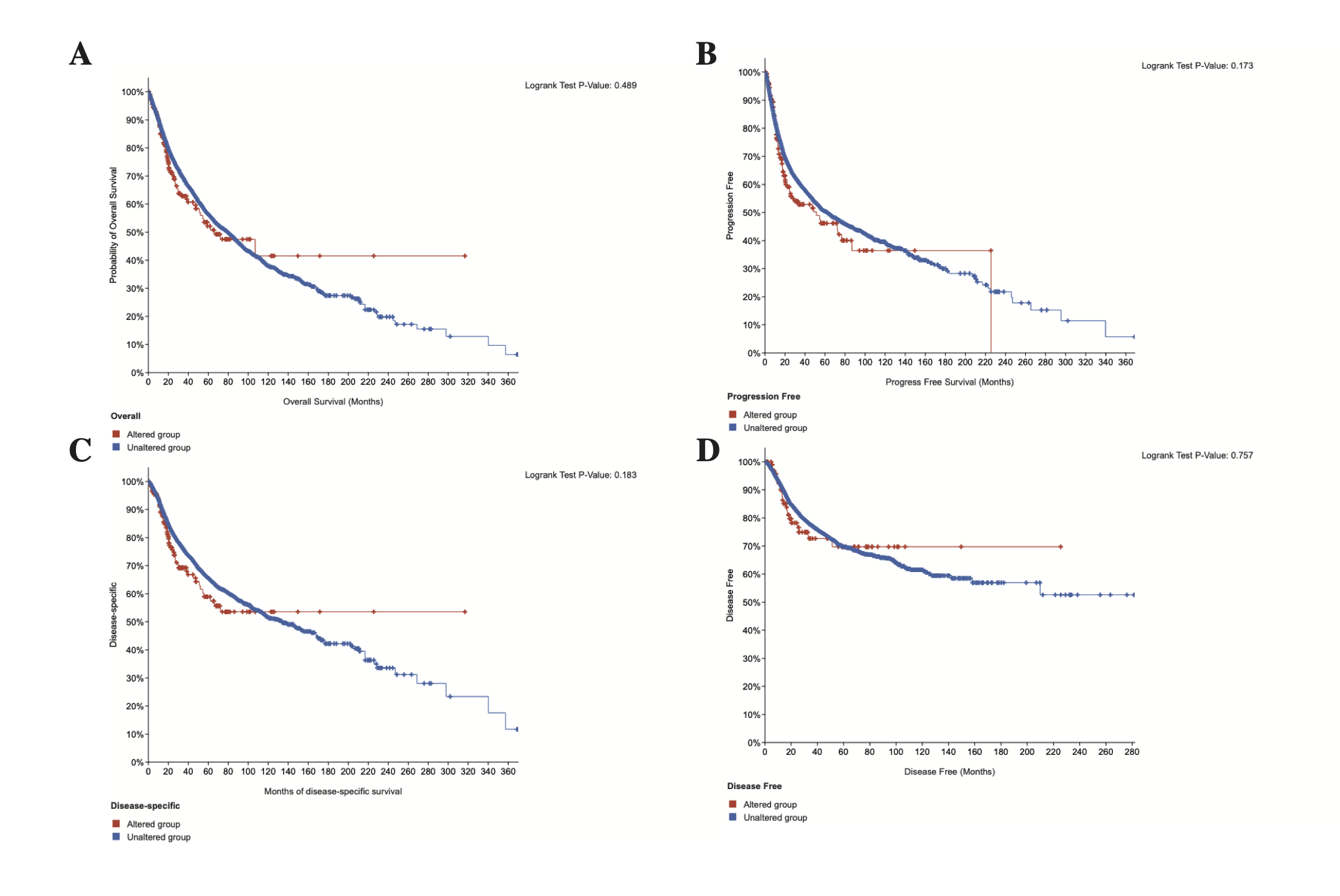

Supplement: Supplementary file 1 [file Image3.TIFF]

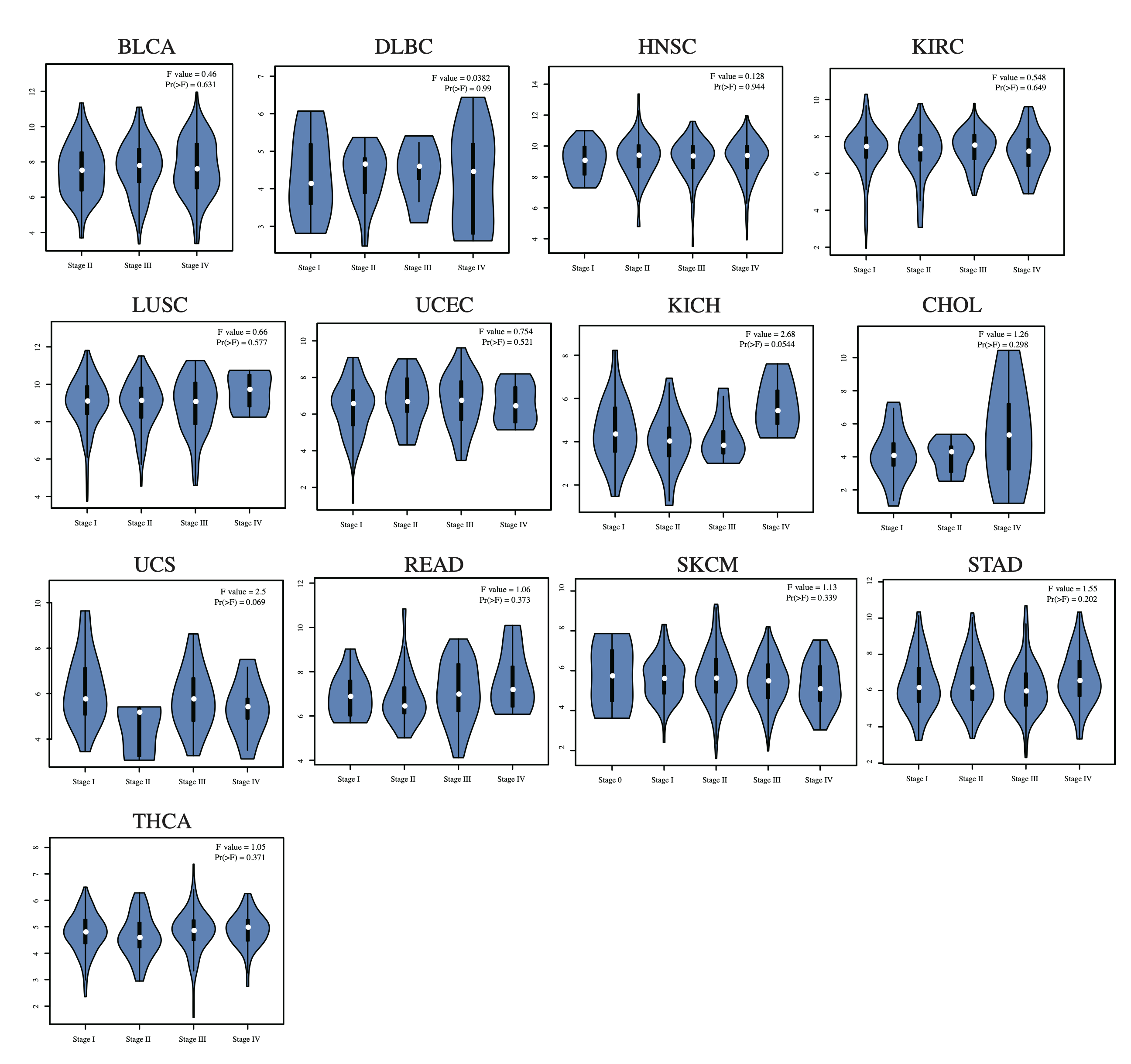

Supplement: Supplementary file 2 [file Image1.TIFF]

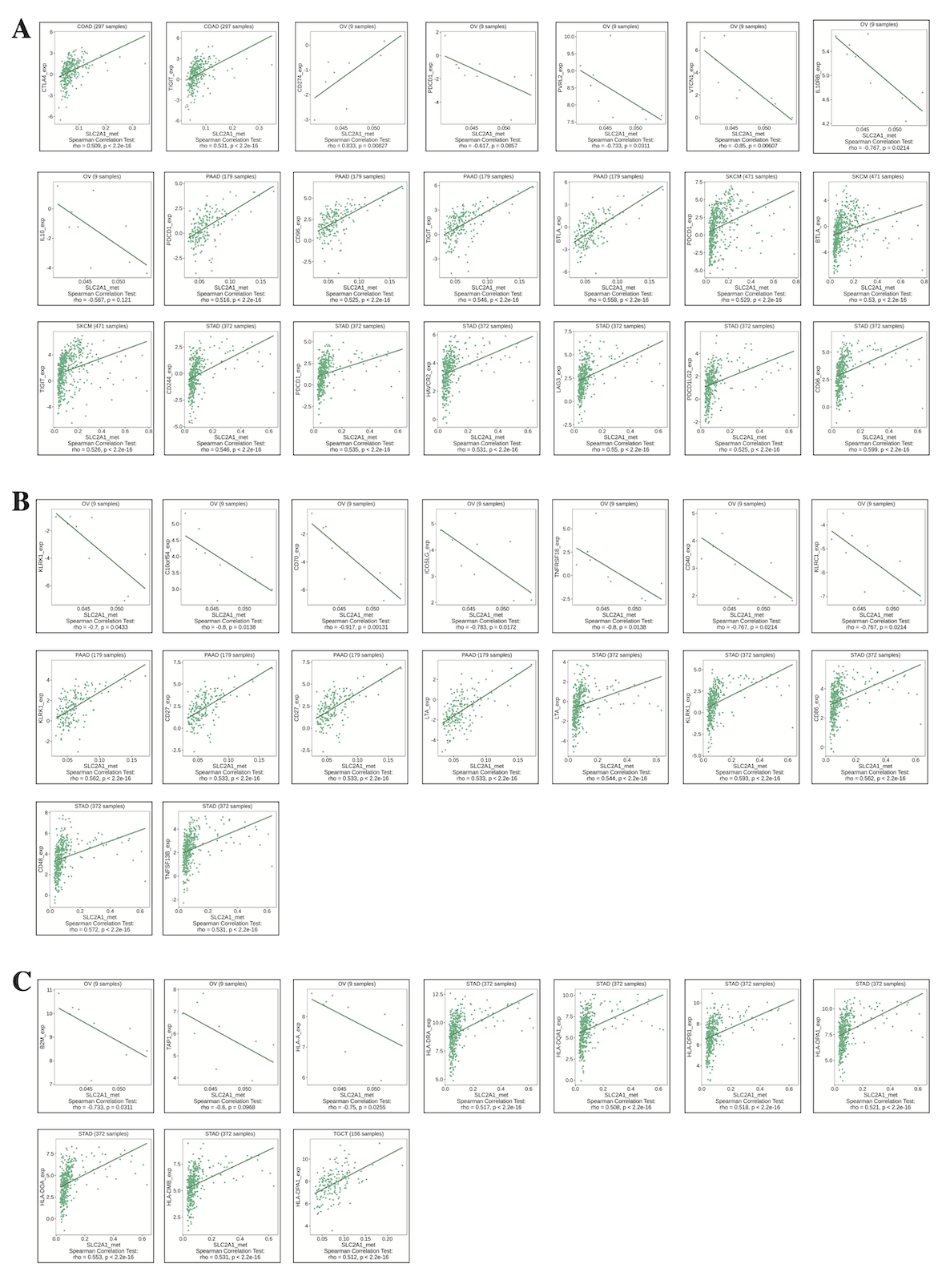

Supplement: Supplementary file 4 [file Image5.TIFF]

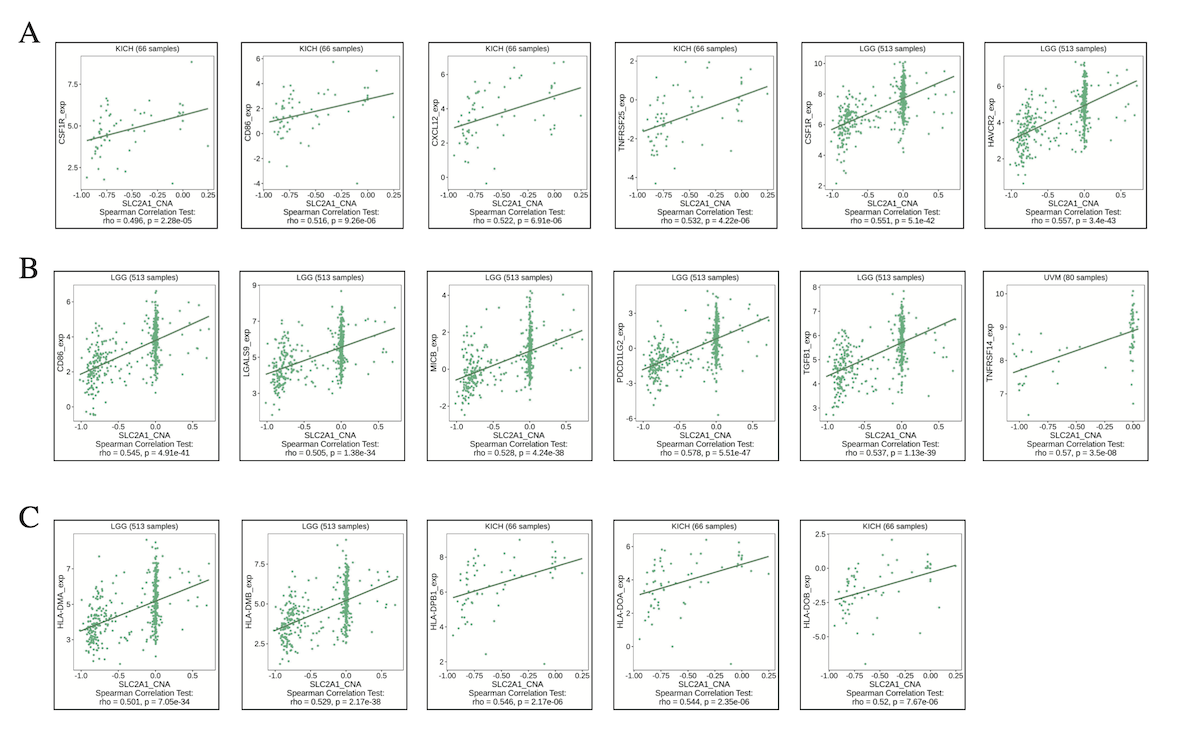

Supplement: Supplementary file 5 [file Image6.TIFF]

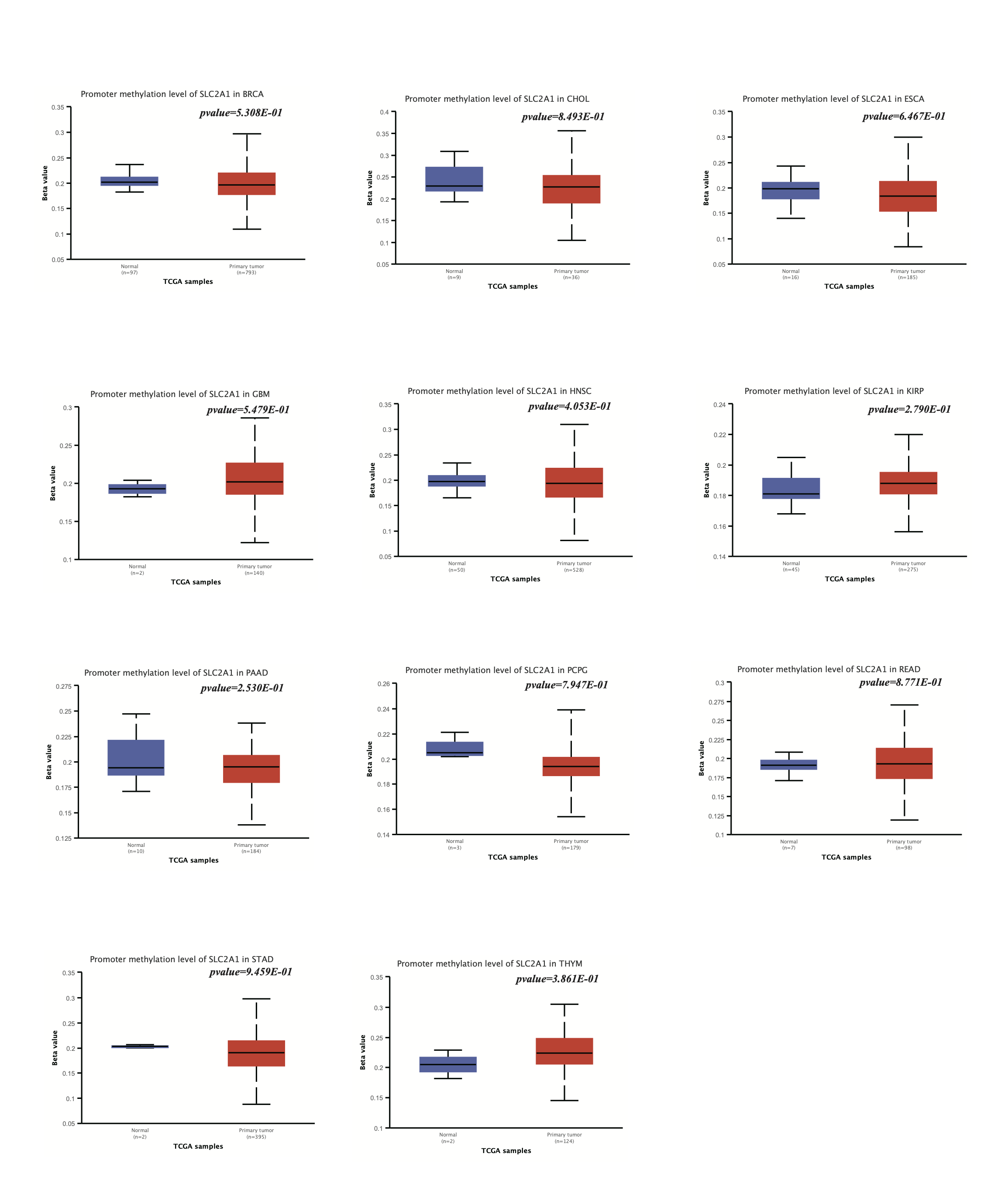

Supplement: Supplementary file 6 [file Image2.TIFF]

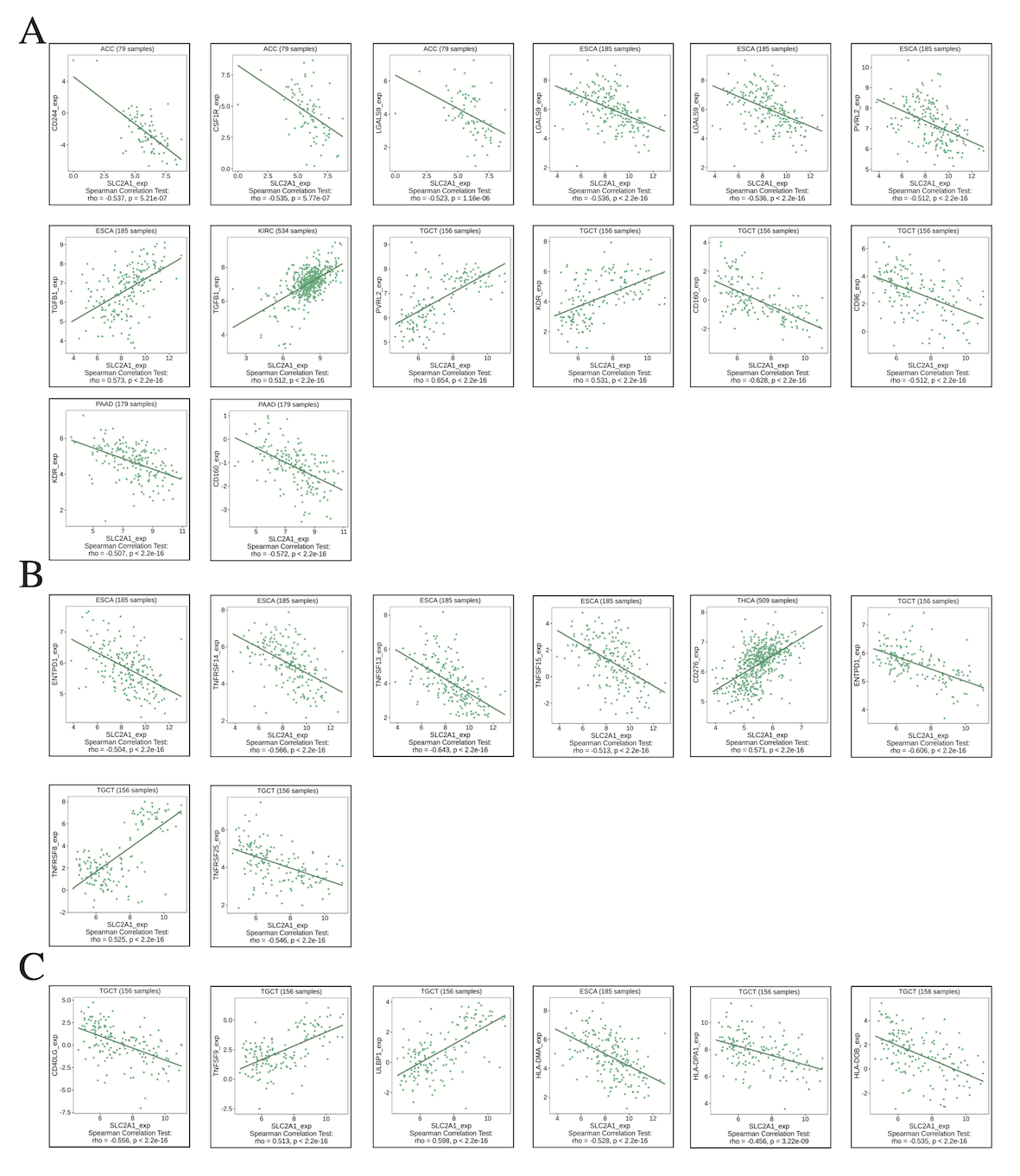

Supplement: Supplementary file 7 [file Image4.TIFF]
