# Supplementary material for: Cell death-related biomarker SLC2A1 has a significant role in prognosis prediction and immunotherapy efficacy evaluation in pan-cancer
Source: Front Genet. 2023 Jan 11;13:1068462. doi: 10.3389/fgene.2022.1068462 (PMC9873976; doi:10.3389/fgene.2022.1068462)
Supplement: Supplementary file 3 [file Table1.DOCX]

Supplementary Material

# Supplementary Tables

**Supplementary Table 1.** The results of the correlation between SLC2A1 expression and different immune infiltrating cells in each cancer (only terms of pvalue <0.05 were kept).

| cancer | cell_type | cor | pvalue |
| --- | --- | --- | --- |
| ACC | Myeloid dendritic cell activated | -0.3385065 | 0.00227792 |
| ACC | T cell CD4+ naive | -0.3498818 | 0.00157341 |
| ACC | T cell CD8+ | -0.3317988 | 0.00281545 |
| ACC | T cell CD8+ central memory | -0.3567191 | 0.00125133 |
| ACC | Myeloid dendritic cell | -0.3812309 | 0.00052761 |
| ACC | Endothelial cell | -0.4734421 | 1.34E-05 |
| ACC | Hematopoietic stem cell | -0.3898099 | 0.0003837 |
| ACC | Macrophage | -0.3871861 | 0.00042334 |
| ACC | Macrophage M1 | -0.3525653 | 0.00143901 |
| ACC | Macrophage M2 | -0.428843 | 8.04E-05 |
| ACC | Mast cell | -0.2227984 | 0.04842794 |
| ACC | Monocyte | -0.3743002 | 0.00067819 |
| ACC | Plasmacytoid dendritic cell | -0.3628751 | 0.00101373 |
| ACC | B cell plasma | 0.35205856 | 0.00146356 |
| ACC | T cell CD4+ Th1 | 0.2256708 | 0.04553144 |
| ACC | T cell regulatory (Tregs) | -0.2803338 | 0.01233754 |
| ACC | immune score | -0.41463 | 0.00016588 |
| ACC | stroma score | -0.441261 | 5.60E-05 |
| ACC | microenvironment score | -0.4789192 | 1.04E-05 |
| BLCA | Myeloid dendritic cell activated | -0.1439429 | 0.00357091 |
| BLCA | B cell | -0.1769696 | 0.00032811 |
| BLCA | T cell CD4+ naive | -0.2791946 | 9.66E-09 |
| BLCA | T cell CD4+ effector memory | 0.10062069 | 0.04221842 |
| BLCA | T cell CD8+ | -0.1189427 | 0.01623045 |
| BLCA | T cell CD8+ effector memory | -0.099285 | 0.04504214 |
| BLCA | Common lymphoid progenitor | 0.1428733 | 0.00382926 |
| BLCA | Myeloid dendritic cell | -0.2480958 | 3.87E-07 |
| BLCA | Endothelial cell | -0.3053389 | 2.98E-10 |
| BLCA | Cancer associated fibroblast | -0.190471 | 0.00010838 |
| BLCA | Hematopoietic stem cell | -0.2225304 | 5.68E-06 |
| BLCA | Macrophage M1 | -0.1458848 | 0.00314188 |
| BLCA | Macrophage M2 | -0.224085 | 4.86E-06 |
| BLCA | Mast cell | 0.16995501 | 0.00056579 |
| BLCA | B cell memory | -0.1124237 | 0.02314184 |
| BLCA | B cell naive | -0.1170031 | 0.01806797 |
| BLCA | T cell NK | -0.1344755 | 0.0065222 |
| BLCA | T cell CD4+ Th2 | 0.0985783 | 0.04659929 |
| BLCA | immune score | -0.1572496 | 0.0014575 |
| BLCA | stroma score | -0.3031846 | 4.03E-10 |
| BLCA | microenvironment score | -0.3051091 | 3.80E-10 |
| BRCA | T cell CD4+ memory | 0.06589735 | 0.02856505 |
| BRCA | T cell CD4+ naive | -0.1514245 | 4.31E-07 |
| BRCA | T cell CD8+ | -0.0778049 | 0.00970473 |
| BRCA | T cell CD8+ central memory | -0.0955235 | 0.00148508 |
| BRCA | Common lymphoid progenitor | 0.12666258 | 2.43E-05 |
| BRCA | Common myeloid progenitor | -0.1197128 | 6.68E-05 |
| BRCA | Endothelial cell | -0.2040945 | 7.60E-12 |
| BRCA | Cancer associated fibroblast | -0.2253891 | 3.51E-14 |
| BRCA | Hematopoietic stem cell | -0.2212504 | 1.04E-13 |
| BRCA | Macrophage M1 | 0.12046153 | 6.01E-05 |
| BRCA | Macrophage M2 | -0.182199 | 1.07E-09 |
| BRCA | Mast cell | -0.0899175 | 0.00278687 |
| BRCA | T cell CD4+ Th1 | 0.18256879 | 9.92E-10 |
| BRCA | T cell CD4+ Th2 | 0.26081025 | 1.26E-18 |
| BRCA | T cell regulatory (Tregs) | -0.1219018 | 4.89E-05 |
| BRCA | stroma score | -0.2471294 | 7.98E-17 |
| BRCA | microenvironment score | -0.2148494 | 5.39E-13 |
| CESC | T cell CD4+ central memory | 0.18594196 | 0.00106312 |
| CESC | Common lymphoid progenitor | 0.20447787 | 0.00031059 |
| CESC | Myeloid dendritic cell | -0.1669385 | 0.00334969 |
| CESC | Endothelial cell | -0.1745125 | 0.0021491 |
| CESC | Cancer associated fibroblast | -0.3353573 | 1.67E-09 |
| CESC | Macrophage M2 | -0.1646777 | 0.00381102 |
| CESC | Neutrophil | 0.23328206 | 3.66E-05 |
| CESC | B cell plasma | -0.3238041 | 6.34E-09 |
| CESC | T cell gamma delta | 0.27116794 | 1.42E-06 |
| CESC | T cell CD4+ Th2 | 0.15370078 | 0.00697323 |
| CESC | T cell regulatory (Tregs) | 0.13886993 | 0.01488842 |
| CESC | stroma score | -0.305524 | 4.69E-08 |
| CESC | microenvironment score | -0.2166767 | 0.00012995 |
| CHOL | T cell CD4+ central memory | 0.37036279 | 0.02618272 |
| CHOL | Common myeloid progenitor | 0.3580777 | 0.03200758 |
| CHOL | Granulocyte-monocyte progenitor | 0.39390597 | 0.01745282 |
| CHOL | Plasmacytoid dendritic cell | 0.34821943 | 0.03741386 |
| CHOL | T cell gamma delta | -0.3730691 | 0.02502498 |
| CHOL | T cell CD4+ Th2 | 0.33367682 | 0.04672155 |
| CHOL | stroma score | -0.3680824 | 0.02784092 |
| COAD | T cell CD8+ naive | -0.0952356 | 0.04253687 |
| COAD | Eosinophil | 0.17481899 | 0.00018146 |
| COAD | Plasmacytoid dendritic cell | -0.1053294 | 0.02481046 |
| ESCA | B cell | -0.2076207 | 0.00457149 |
| ESCA | T cell CD8+ | -0.275652 | 0.00014604 |
| ESCA | T cell CD8+ effector memory | 0.16643973 | 0.02355577 |
| ESCA | Class-switched memory B cell | -0.2058409 | 0.00493988 |
| ESCA | Common lymphoid progenitor | 0.26615008 | 0.00025048 |
| ESCA | Endothelial cell | -0.4540127 | 8.54E-11 |
| ESCA | Granulocyte-monocyte progenitor | 0.19525631 | 0.0077342 |
| ESCA | Hematopoietic stem cell | -0.3618749 | 4.16E-07 |
| ESCA | Macrophage M2 | -0.3789127 | 1.05E-07 |
| ESCA | B cell memory | -0.273428 | 0.00016599 |
| ESCA | T cell NK | -0.3158778 | 1.19E-05 |
| ESCA | B cell plasma | -0.4115831 | 5.88E-09 |
| ESCA | T cell CD4+ Th1 | 0.15038261 | 0.04103313 |
| ESCA | T cell CD4+ Th2 | 0.31623825 | 1.16E-05 |
| ESCA | immune score | -0.1467344 | 0.04632729 |
| ESCA | stroma score | -0.4621176 | 3.56E-11 |
| ESCA | microenvironment score | -0.3984326 | 2.59E-08 |
| GBM | B cell | -0.1589812 | 0.04015713 |
| GBM | T cell CD8+ naive | -0.1688699 | 0.02914046 |
| GBM | Class-switched memory B cell | -0.2096615 | 0.00654074 |
| HNSC | Myeloid dendritic cell activated | -0.2934718 | 7.91E-12 |
| HNSC | B cell | -0.2414878 | 2.31E-08 |
| HNSC | T cell CD4+ memory | -0.1681141 | 0.00011374 |
| HNSC | T cell CD4+ naive | -0.2560067 | 2.95E-09 |
| HNSC | T cell CD4+ (non-regulatory) | -0.0938347 | 0.03207592 |
| HNSC | T cell CD4+ central memory | 0.18876621 | 1.41E-05 |
| HNSC | T cell CD8+ naive | -0.1220987 | 0.00521574 |
| HNSC | T cell CD8+ | -0.1187404 | 0.00660735 |
| HNSC | T cell CD8+ central memory | -0.2364257 | 4.59E-08 |
| HNSC | T cell CD8+ effector memory | -0.1292447 | 0.00309409 |
| HNSC | Myeloid dendritic cell | -0.2677405 | 5.09E-10 |
| HNSC | Endothelial cell | -0.1176666 | 0.00711793 |
| HNSC | Eosinophil | 0.12396031 | 0.0045637 |
| HNSC | Cancer associated fibroblast | -0.3434416 | 6.76E-16 |
| HNSC | Granulocyte-monocyte progenitor | -0.0935916 | 0.03252607 |
| HNSC | Hematopoietic stem cell | -0.2234092 | 2.50E-07 |
| HNSC | Macrophage | -0.2008679 | 3.74E-06 |
| HNSC | Macrophage M1 | -0.2832081 | 4.39E-11 |
| HNSC | Macrophage M2 | -0.1332973 | 0.00227463 |
| HNSC | Mast cell | 0.143426 | 0.00101604 |
| HNSC | B cell memory | -0.1333254 | 0.00226971 |
| HNSC | B cell naive | -0.1060666 | 0.01533495 |
| HNSC | Neutrophil | 0.12853234 | 0.0032632 |
| HNSC | Plasmacytoid dendritic cell | -0.2076227 | 1.71E-06 |
| HNSC | B cell plasma | -0.1453713 | 0.00086505 |
| HNSC | T cell CD4+ Th1 | -0.1658197 | 0.00014133 |
| HNSC | immune score | -0.265746 | 8.05E-10 |
| HNSC | stroma score | -0.3085726 | 5.60E-13 |
| HNSC | microenvironment score | -0.3967342 | 0 |
| KICH | B cell | -0.3401393 | 0.00520044 |
| KICH | T cell CD8+ central memory | -0.2773118 | 0.02417999 |
| KICH | Endothelial cell | -0.306419 | 0.01262622 |
| KICH | Hematopoietic stem cell | -0.2991963 | 0.01496788 |
| KICH | NK cell | 0.3678133 | 0.00237907 |
| KICH | T cell regulatory (Tregs) | -0.2563715 | 0.03772383 |
| KIRC | B cell | -0.1031545 | 0.01710152 |
| KIRC | T cell CD8+ naive | -0.1513228 | 0.00045002 |
| KIRC | T cell CD8+ effector memory | -0.116019 | 0.00727898 |
| KIRC | Common lymphoid progenitor | 0.15423993 | 0.00034713 |
| KIRC | Cancer associated fibroblast | 0.13559679 | 0.001686 |
| KIRC | Macrophage M1 | 0.10818712 | 0.01236604 |
| KIRC | Macrophage M2 | -0.1177557 | 0.00644461 |
| KIRC | Mast cell | -0.1838802 | 1.91E-05 |
| KIRC | Monocyte | 0.13628888 | 0.00159519 |
| KIRC | Neutrophil | 0.09261325 | 0.03237635 |
| KIRC | T cell NK | 0.21282559 | 6.92E-07 |
| KIRC | B cell plasma | -0.1201484 | 0.00543568 |
| KIRC | stroma score | 0.13465505 | 0.00183184 |
| KIRP | T cell CD4+ memory | 0.14357182 | 0.01423321 |
| KIRP | T cell CD4+ central memory | -0.2035856 | 0.00047472 |
| KIRP | T cell CD4+ effector memory | -0.259039 | 7.59E-06 |
| KIRP | T cell CD8+ naive | -0.1809604 | 0.00193983 |
| KIRP | T cell CD8+ | -0.1450378 | 0.01326329 |
| KIRP | T cell CD8+ central memory | 0.14940844 | 0.01070796 |
| KIRP | Common myeloid progenitor | -0.127301 | 0.02992468 |
| KIRP | Endothelial cell | 0.33762879 | 3.44E-09 |
| KIRP | Hematopoietic stem cell | 0.23518741 | 5.33E-05 |
| KIRP | Mast cell | -0.1251506 | 0.03283319 |
| KIRP | Neutrophil | 0.13110425 | 0.02531859 |
| KIRP | Plasmacytoid dendritic cell | -0.1486753 | 0.01110355 |
| KIRP | T cell CD4+ Th2 | 0.2194431 | 0.00016104 |
| KIRP | T cell regulatory (Tregs) | -0.2060903 | 0.00040231 |
| KIRP | stroma score | 0.18290179 | 0.001756 |
| LGG | B cell | -0.1083684 | 0.01254993 |
| LGG | T cell CD8+ central memory | -0.0964551 | 0.02638479 |
| LGG | Class-switched memory B cell | -0.0867538 | 0.04590429 |
| LGG | Endothelial cell | 0.5195944 | 5.54E-38 |
| LGG | Eosinophil | 0.11300234 | 0.00922204 |
| LGG | Hematopoietic stem cell | 0.33396133 | 2.83E-15 |
| LGG | Mast cell | -0.148286 | 0.0006155 |
| LGG | Neutrophil | 0.1182897 | 0.00640329 |
| LGG | T cell NK | -0.0882203 | 0.04234148 |
| LGG | T cell CD4+ Th1 | -0.1039455 | 0.01667294 |
| LGG | T cell CD4+ Th2 | -0.2273499 | 1.22E-07 |
| LGG | stroma score | 0.34397283 | 3.63E-16 |
| LGG | microenvironment score | 0.20709418 | 1.52E-06 |
| LIHC | Myeloid dendritic cell activated | 0.21673029 | 2.42E-05 |
| LIHC | B cell | 0.26156882 | 2.99E-07 |
| LIHC | T cell CD4+ memory | 0.18747956 | 0.00027157 |
| LIHC | T cell CD4+ central memory | -0.1221621 | 0.01826161 |
| LIHC | T cell CD8+ | 0.17104157 | 0.00091072 |
| LIHC | Class-switched memory B cell | 0.12397535 | 0.01659437 |
| LIHC | Common lymphoid progenitor | 0.26168723 | 2.95E-07 |
| LIHC | Myeloid dendritic cell | 0.13002379 | 0.01195647 |
| LIHC | Endothelial cell | -0.2385579 | 3.17E-06 |
| LIHC | Eosinophil | -0.1357055 | 0.0086837 |
| LIHC | Hematopoietic stem cell | -0.2593669 | 3.78E-07 |
| LIHC | Macrophage | 0.12168299 | 0.01872588 |
| LIHC | Macrophage M1 | 0.18965744 | 0.00022952 |
| LIHC | Macrophage M2 | -0.1442874 | 0.00523958 |
| LIHC | Monocyte | 0.25668115 | 5.03E-07 |
| LIHC | B cell plasma | -0.162355 | 0.00165538 |
| LIHC | T cell CD4+ Th2 | 0.26520529 | 2.01E-07 |
| LIHC | immune score | 0.2114672 | 3.99E-05 |
| LIHC | stroma score | -0.2620066 | 3.15E-07 |
| LUAD | T cell CD4+ naive | -0.2718913 | 3.26E-10 |
| LUAD | T cell CD4+ central memory | -0.3859432 | 8.28E-20 |
| LUAD | T cell CD4+ effector memory | -0.0982023 | 0.02555664 |
| LUAD | T cell CD8+ | -0.2479015 | 1.11E-08 |
| LUAD | Class-switched memory B cell | -0.1601108 | 0.00025684 |
| LUAD | Common lymphoid progenitor | 0.23794548 | 4.35E-08 |
| LUAD | Common myeloid progenitor | -0.1764598 | 5.48E-05 |
| LUAD | Myeloid dendritic cell | -0.1333228 | 0.00238408 |
| LUAD | Endothelial cell | -0.181048 | 3.46E-05 |
| LUAD | Eosinophil | -0.1710539 | 9.28E-05 |
| LUAD | Cancer associated fibroblast | -0.2583025 | 2.52E-09 |
| LUAD | Granulocyte-monocyte progenitor | -0.164388 | 0.00017382 |
| LUAD | Hematopoietic stem cell | -0.4621191 | 1.03E-28 |
| LUAD | Macrophage M1 | 0.14772227 | 0.00075379 |
| LUAD | Macrophage M2 | -0.2010863 | 4.06E-06 |
| LUAD | Mast cell | -0.2104935 | 1.37E-06 |
| LUAD | NK cell | 0.10062281 | 0.02212606 |
| LUAD | Plasmacytoid dendritic cell | 0.22292184 | 3.04E-07 |
| LUAD | T cell CD4+ Th1 | 0.24945093 | 8.96E-09 |
| LUAD | T cell CD4+ Th2 | 0.48333445 | 1.27E-31 |
| LUAD | immune score | -0.093726 | 0.03311872 |
| LUAD | stroma score | -0.2751233 | 1.97E-10 |
| LUAD | microenvironment score | -0.1835713 | 2.67E-05 |
| LUSC | Myeloid dendritic cell activated | -0.3601702 | 8.03E-17 |
| LUSC | B cell | -0.3484019 | 8.99E-16 |
| LUSC | T cell CD4+ memory | -0.1773091 | 6.48E-05 |
| LUSC | T cell CD4+ naive | -0.2499583 | 1.37E-08 |
| LUSC | T cell CD4+ (non-regulatory) | -0.1490628 | 0.00080749 |
| LUSC | T cell CD4+ effector memory | -0.0938817 | 0.03547845 |
| LUSC | T cell CD8+ naive | -0.1837363 | 3.45E-05 |
| LUSC | T cell CD8+ | -0.3708646 | 8.17E-18 |
| LUSC | T cell CD8+ central memory | -0.3547117 | 2.49E-16 |
| LUSC | T cell CD8+ effector memory | -0.1469841 | 0.00095664 |
| LUSC | Class-switched memory B cell | -0.2673285 | 1.16E-09 |
| LUSC | Myeloid dendritic cell | -0.2361805 | 8.58E-08 |
| LUSC | Endothelial cell | -0.2376162 | 7.13E-08 |
| LUSC | Cancer associated fibroblast | -0.2267225 | 2.83E-07 |
| LUSC | Hematopoietic stem cell | -0.1413296 | 0.00150046 |
| LUSC | Macrophage | -0.2580686 | 4.43E-09 |
| LUSC | Macrophage M1 | -0.3004755 | 6.20E-12 |
| LUSC | Macrophage M2 | -0.2208 | 5.84E-07 |
| LUSC | Mast cell | -0.1024941 | 0.02163342 |
| LUSC | B cell memory | -0.2465677 | 2.18E-08 |
| LUSC | Monocyte | -0.1118639 | 0.01214238 |
| LUSC | B cell naive | -0.2235657 | 4.18E-07 |
| LUSC | T cell NK | -0.2274552 | 2.59E-07 |
| LUSC | Plasmacytoid dendritic cell | -0.3326231 | 1.96E-14 |
| LUSC | B cell plasma | -0.1596099 | 0.00033037 |
| LUSC | T cell regulatory (Tregs) | -0.1727548 | 0.00010013 |
| LUSC | immune score | -0.3704277 | 8.99E-18 |
| LUSC | stroma score | -0.2584933 | 4.17E-09 |
| LUSC | microenvironment score | -0.3773874 | 1.94E-18 |
| MESO | T cell CD4+ central memory | -0.3426623 | 0.00115866 |
| MESO | Myeloid dendritic cell | -0.3030771 | 0.0043245 |
| MESO | Mast cell | -0.2267307 | 0.03470351 |
| MESO | B cell memory | -0.3047113 | 0.00410957 |
| MESO | T cell CD4+ Th2 | 0.41755249 | 5.73E-05 |
| MESO | microenvironment score | -0.2636509 | 0.01382521 |
| OV | Myeloid dendritic cell activated | -0.1442909 | 0.01123575 |
| OV | B cell | -0.1229701 | 0.03096395 |
| OV | Hematopoietic stem cell | -0.1333562 | 0.01921457 |
| OV | Macrophage | -0.114771 | 0.04414737 |
| OV | Macrophage M1 | -0.1253989 | 0.02777246 |
| OV | B cell memory | -0.1414932 | 0.0129327 |
| OV | T cell NK | -0.1172398 | 0.0397555 |
| OV | Plasmacytoid dendritic cell | -0.1343068 | 0.01836455 |
| OV | immune score | -0.161332 | 0.0045734 |
| OV | microenvironment score | -0.1544449 | 0.00665883 |
| PAAD | Myeloid dendritic cell activated | -0.2073024 | 0.00536271 |
| PAAD | B cell | -0.1641464 | 0.02811641 |
| PAAD | T cell CD4+ naive | -0.2724419 | 0.00022467 |
| PAAD | T cell CD4+ central memory | 0.16281871 | 0.02943158 |
| PAAD | T cell CD8+ | -0.4218111 | 4.09E-09 |
| PAAD | T cell CD8+ central memory | -0.2770835 | 0.00017338 |
| PAAD | Class-switched memory B cell | -0.2140611 | 0.00401085 |
| PAAD | Common lymphoid progenitor | 0.29202095 | 7.30E-05 |
| PAAD | Common myeloid progenitor | -0.1665695 | 0.02584539 |
| PAAD | Myeloid dendritic cell | -0.1615196 | 0.0307689 |
| PAAD | Endothelial cell | -0.4864463 | 5.07E-12 |
| PAAD | Eosinophil | 0.16003718 | 0.03235771 |
| PAAD | Cancer associated fibroblast | -0.3078286 | 2.77E-05 |
| PAAD | Granulocyte-monocyte progenitor | -0.3473412 | 1.90E-06 |
| PAAD | Hematopoietic stem cell | -0.4728789 | 2.78E-11 |
| PAAD | Macrophage M2 | -0.368916 | 4.57E-07 |
| PAAD | Mast cell | -0.2219532 | 0.00282593 |
| PAAD | T cell CD4+ Th1 | 0.19842966 | 0.00775073 |
| PAAD | T cell CD4+ Th2 | 0.25825266 | 0.00048234 |
| PAAD | immune score | -0.1794907 | 0.01631311 |
| PAAD | stroma score | -0.3658674 | 5.75E-07 |
| PAAD | microenvironment score | -0.3406189 | 3.54E-06 |
| PCPG | Myeloid dendritic cell activated | 0.29605906 | 4.50E-05 |
| PCPG | B cell | -0.2193288 | 0.00277742 |
| PCPG | T cell CD4+ naive | 0.23315186 | 0.00144711 |
| PCPG | Class-switched memory B cell | -0.2524502 | 0.00054575 |
| PCPG | Common lymphoid progenitor | -0.2872725 | 7.69E-05 |
| PCPG | Common myeloid progenitor | 0.19721502 | 0.00728931 |
| PCPG | Myeloid dendritic cell | 0.18068356 | 0.01411026 |
| PCPG | Endothelial cell | 0.46707079 | 2.33E-11 |
| PCPG | Eosinophil | -0.149393 | 0.04296726 |
| PCPG | Granulocyte-monocyte progenitor | 0.18484147 | 0.01200803 |
| PCPG | Hematopoietic stem cell | 0.40137235 | 1.64E-08 |
| PCPG | Macrophage M1 | 0.17684192 | 0.01633199 |
| PCPG | Macrophage M2 | -0.2636163 | 0.00029958 |
| PCPG | Mast cell | -0.3108449 | 1.75E-05 |
| PCPG | Neutrophil | 0.16713057 | 0.02335403 |
| PCPG | T cell NK | -0.2535529 | 0.00051497 |
| PCPG | B cell plasma | -0.3044486 | 2.65E-05 |
| PCPG | T cell CD4+ Th1 | -0.3232774 | 7.60E-06 |
| PCPG | immune score | -0.2090963 | 0.00445934 |
| PCPG | stroma score | 0.40952207 | 1.07E-08 |
| PCPG | microenvironment score | 0.37349117 | 2.20E-07 |
| PRAD | B cell | 0.12435355 | 0.00545461 |
| PRAD | T cell CD4+ effector memory | 0.09088793 | 0.04262683 |
| PRAD | T cell CD8+ naive | -0.1427937 | 0.00139854 |
| PRAD | Common lymphoid progenitor | -0.1465056 | 0.00104212 |
| PRAD | Endothelial cell | -0.1052108 | 0.01884943 |
| PRAD | Cancer associated fibroblast | -0.1650626 | 0.00021588 |
| PRAD | Granulocyte-monocyte progenitor | -0.1204149 | 0.00714069 |
| PRAD | Hematopoietic stem cell | -0.2551766 | 7.65E-09 |
| PRAD | Mast cell | 0.1684663 | 0.00015869 |
| PRAD | Plasmacytoid dendritic cell | 0.11263458 | 0.01189528 |
| PRAD | B cell plasma | 0.12768376 | 0.00431849 |
| PRAD | T cell regulatory (Tregs) | 0.10808553 | 0.01582013 |
| PRAD | stroma score | -0.1687864 | 0.00015412 |
| PRAD | microenvironment score | -0.1380258 | 0.00202037 |
| READ | B cell | -0.193008 | 0.01416603 |
| READ | T cell CD4+ central memory | -0.1602312 | 0.04231573 |
| READ | Class-switched memory B cell | -0.1908587 | 0.0153003 |
| READ | Endothelial cell | -0.161994 | 0.04006884 |
| READ | Eosinophil | 0.19120668 | 0.01511144 |
| READ | B cell naive | -0.1855588 | 0.01844134 |
| READ | B cell plasma | -0.2426834 | 0.00192336 |
| READ | T cell gamma delta | -0.2152204 | 0.00611094 |
| READ | T cell CD4+ Th1 | -0.2247068 | 0.00416042 |
| READ | T cell CD4+ Th2 | 0.15795951 | 0.04545396 |
| SARC | T cell CD4+ naive | -0.1938009 | 0.00158886 |
| SARC | T cell CD4+ central memory | -0.1395616 | 0.02359768 |
| SARC | T cell CD8+ central memory | -0.2165151 | 0.00040531 |
| SARC | Common lymphoid progenitor | 0.3212061 | 1.00E-07 |
| SARC | Common myeloid progenitor | -0.3361006 | 2.30E-08 |
| SARC | Myeloid dendritic cell | -0.1347185 | 0.0289382 |
| SARC | Endothelial cell | -0.2823367 | 3.29E-06 |
| SARC | Cancer associated fibroblast | -0.355603 | 2.95E-09 |
| SARC | Granulocyte-monocyte progenitor | -0.2222602 | 0.00028033 |
| SARC | Hematopoietic stem cell | -0.3919296 | 4.36E-11 |
| SARC | Macrophage M2 | -0.1615382 | 0.0086779 |
| SARC | B cell memory | -0.1266073 | 0.04019804 |
| SARC | Plasmacytoid dendritic cell | 0.12373266 | 0.04498887 |
| SARC | T cell CD4+ Th1 | 0.12247321 | 0.04723391 |
| SARC | T cell CD4+ Th2 | 0.25635707 | 2.57E-05 |
| SARC | stroma score | -0.3968074 | 3.37E-11 |
| SARC | microenvironment score | -0.309783 | 3.40E-07 |
| SKCM | Myeloid dendritic cell activated | -0.197457 | 1.52E-05 |
| SKCM | B cell | -0.28436 | 3.00E-10 |
| SKCM | T cell CD4+ memory | -0.2821959 | 4.14E-10 |
| SKCM | T cell CD4+ (non-regulatory) | -0.154374 | 0.00075512 |
| SKCM | T cell CD8+ naive | -0.2097087 | 4.22E-06 |
| SKCM | T cell CD8+ | -0.1838376 | 5.77E-05 |
| SKCM | T cell CD8+ central memory | -0.2251696 | 7.50E-07 |
| SKCM | Class-switched memory B cell | -0.2037174 | 7.98E-06 |
| SKCM | Myeloid dendritic cell | -0.1172811 | 0.01068726 |
| SKCM | Eosinophil | 0.10198959 | 0.02655179 |
| SKCM | Hematopoietic stem cell | -0.2124675 | 3.13E-06 |
| SKCM | Macrophage | -0.1289115 | 0.00498584 |
| SKCM | Macrophage M1 | -0.1219131 | 0.00794666 |
| SKCM | Macrophage M2 | -0.1287238 | 0.00505005 |
| SKCM | Mast cell | -0.2069729 | 5.66E-06 |
| SKCM | B cell memory | -0.1194319 | 0.00932477 |
| SKCM | B cell naive | -0.1968258 | 1.62E-05 |
| SKCM | Plasmacytoid dendritic cell | -0.2255646 | 7.17E-07 |
| SKCM | B cell plasma | -0.1841172 | 5.62E-05 |
| SKCM | T cell regulatory (Tregs) | -0.2285254 | 5.07E-07 |
| SKCM | immune score | -0.2447793 | 7.70E-08 |
| SKCM | microenvironment score | -0.2370112 | 2.00E-07 |
| STAD | Myeloid dendritic cell activated | -0.2203339 | 5.88E-06 |
| STAD | B cell | -0.24699 | 3.48E-07 |
| STAD | T cell CD4+ memory | -0.171437 | 0.00045165 |
| STAD | T cell CD4+ naive | -0.2428406 | 5.52E-07 |
| STAD | T cell CD8+ naive | -0.251183 | 2.16E-07 |
| STAD | T cell CD8+ | -0.2272362 | 2.92E-06 |
| STAD | T cell CD8+ effector memory | -0.1928762 | 7.67E-05 |
| STAD | Class-switched memory B cell | -0.2554164 | 1.33E-07 |
| STAD | Endothelial cell | -0.3457536 | 4.26E-13 |
| STAD | Cancer associated fibroblast | -0.3056027 | 2.02E-10 |
| STAD | Hematopoietic stem cell | -0.4238106 | 1.61E-19 |
| STAD | Macrophage | -0.1244672 | 0.01115459 |
| STAD | Macrophage M2 | -0.2497792 | 2.54E-07 |
| STAD | Mast cell | -0.1598412 | 0.00108531 |
| STAD | B cell memory | -0.2009291 | 3.74E-05 |
| STAD | B cell naive | -0.1847199 | 0.00015414 |
| STAD | T cell NK | -0.1667386 | 0.00064872 |
| STAD | Plasmacytoid dendritic cell | -0.1046402 | 0.03308034 |
| STAD | T cell CD4+ Th1 | 0.16078233 | 0.00101291 |
| STAD | immune score | -0.3074645 | 1.55E-10 |
| STAD | stroma score | -0.3801727 | 1.02E-15 |
| STAD | microenvironment score | -0.4979017 | 2.21E-27 |
| TGCT | Myeloid dendritic cell activated | -0.4417923 | 7.77E-09 |
| TGCT | B cell | -0.6180916 | 8.24E-18 |
| TGCT | T cell CD4+ memory | -0.5105633 | 9.82E-12 |
| TGCT | T cell CD4+ naive | -0.481735 | 1.93E-10 |
| TGCT | T cell CD4+ (non-regulatory) | -0.2452312 | 0.0020317 |
| TGCT | T cell CD4+ effector memory | -0.2837706 | 0.00033084 |
| TGCT | T cell CD8+ naive | 0.21117206 | 0.00813929 |
| TGCT | T cell CD8+ | -0.5886366 | 6.41E-16 |
| TGCT | T cell CD8+ central memory | -0.4756199 | 3.51E-10 |
| TGCT | T cell CD8+ effector memory | -0.4895383 | 8.86E-11 |
| TGCT | Class-switched memory B cell | -0.6226505 | 4.03E-18 |
| TGCT | Common lymphoid progenitor | 0.45420687 | 2.59E-09 |
| TGCT | Myeloid dendritic cell | -0.4420569 | 7.59E-09 |
| TGCT | Endothelial cell | 0.45622526 | 2.16E-09 |
| TGCT | Granulocyte-monocyte progenitor | -0.1681815 | 0.03584651 |
| TGCT | Hematopoietic stem cell | 0.52123722 | 0 |
| TGCT | Macrophage M2 | -0.2812993 | 0.00037472 |
| TGCT | Mast cell | -0.5482827 | 1.27E-13 |
| TGCT | B cell memory | -0.3594456 | 4.06E-06 |
| TGCT | Monocyte | -0.1923831 | 0.01612566 |
| TGCT | B cell naive | -0.603323 | 7.73E-17 |
| TGCT | NK cell | -0.2255777 | 0.00463457 |
| TGCT | Plasmacytoid dendritic cell | -0.4387662 | 1.01E-08 |
| TGCT | T cell gamma delta | -0.2853263 | 0.00030571 |
| TGCT | T cell CD4+ Th1 | 0.28779699 | 0.00026942 |
| TGCT | T cell regulatory (Tregs) | -0.5917604 | 4.12E-16 |
| TGCT | immune score | -0.5756824 | 0 |
| TGCT | stroma score | 0.38990691 | 6.06E-07 |
| TGCT | microenvironment score | -0.5308435 | 0 |
| THCA | Myeloid dendritic cell activated | 0.22034777 | 4.64E-07 |
| THCA | B cell | 0.10685939 | 0.01546321 |
| THCA | T cell CD4+ memory | 0.09418725 | 0.03293836 |
| THCA | T cell CD4+ (non-regulatory) | -0.0983515 | 0.02590839 |
| THCA | T cell CD4+ central memory | -0.0924449 | 0.03633061 |
| THCA | T cell CD8+ naive | -0.299573 | 4.26E-12 |
| THCA | Class-switched memory B cell | 0.33990879 | 2.43E-15 |
| THCA | Myeloid dendritic cell | 0.32322018 | 6.13E-14 |
| THCA | Endothelial cell | -0.3533205 | 1.58E-16 |
| THCA | Hematopoietic stem cell | -0.2910552 | 2.22E-11 |
| THCA | Macrophage | 0.18916667 | 1.61E-05 |
| THCA | Macrophage M1 | 0.16039884 | 0.00026456 |
| THCA | Macrophage M2 | -0.208926 | 1.81E-06 |
| THCA | Mast cell | 0.31597569 | 3.11E-13 |
| THCA | Monocyte | 0.15894184 | 0.00030131 |
| THCA | Neutrophil | 0.1054113 | 0.01692437 |
| THCA | T cell NK | 0.22638746 | 2.19E-07 |
| THCA | B cell plasma | -0.4056516 | 9.67E-22 |
| THCA | T cell CD4+ Th1 | -0.2803183 | 1.02E-10 |
| THCA | T cell CD4+ Th2 | 0.16429675 | 0.00018579 |
| THCA | T cell regulatory (Tregs) | 0.10118633 | 0.02189899 |
| THCA | immune score | 0.29967987 | 5.33E-12 |
| THCA | stroma score | -0.314817 | 3.84E-13 |
| THYM | Myeloid dendritic cell activated | 0.25843921 | 0.00437176 |
| THYM | B cell | 0.25339445 | 0.00523073 |
| THYM | Class-switched memory B cell | 0.23772576 | 0.00893606 |
| THYM | Common lymphoid progenitor | -0.1846517 | 0.04361577 |
| THYM | Endothelial cell | -0.2212108 | 0.01518073 |
| THYM | Cancer associated fibroblast | -0.2608961 | 0.00400105 |
| THYM | Macrophage | 0.20313536 | 0.02606789 |
| THYM | Macrophage M1 | 0.2935353 | 0.00113852 |
| THYM | Mast cell | 0.23210709 | 0.01074359 |
| THYM | B cell memory | 0.32711556 | 0.00026561 |
| THYM | B cell naive | 0.30987423 | 0.00057285 |
| THYM | Neutrophil | -0.2346287 | 0.00989611 |
| THYM | T cell NK | -0.3252123 | 0.00028978 |
| THYM | B cell plasma | 0.18839304 | 0.03933825 |
| THYM | stroma score | -0.3042786 | 0.00072802 |
| UCEC | Myeloid dendritic cell activated | -0.1519401 | 0.00043149 |
| UCEC | T cell CD8+ naive | -0.207754 | 1.31E-06 |
| UCEC | Endothelial cell | -0.1836784 | 1.98E-05 |
| UCEC | Cancer associated fibroblast | -0.0894996 | 0.03887055 |
| UCEC | Hematopoietic stem cell | -0.179195 | 3.17E-05 |
| UCEC | Mast cell | 0.08534792 | 0.04890967 |
| UCEC | T cell NK | -0.1491982 | 0.0005487 |
| UCEC | Plasmacytoid dendritic cell | -0.1043531 | 0.01594793 |
| UCEC | stroma score | -0.1695091 | 8.40E-05 |
| UCS | Myeloid dendritic cell activated | -0.2611684 | 0.04972781 |
| UCS | T cell CD8+ naive | -0.309019 | 0.01933965 |
| UCS | T cell CD8+ | -0.3629777 | 0.00551948 |
| UCS | Endothelial cell | -0.3383259 | 0.01004974 |
| UCS | Granulocyte-monocyte progenitor | -0.3481757 | 0.00795351 |
| UCS | T cell NK | -0.2900863 | 0.02860513 |
| UVM | B cell | -0.2527674 | 0.02369081 |
| UVM | T cell CD4+ memory | -0.3420572 | 0.00189924 |
| UVM | T cell CD4+ (non-regulatory) | -0.3363716 | 0.00228332 |
| UVM | T cell CD8+ | -0.2264085 | 0.04343593 |
| UVM | Common lymphoid progenitor | -0.2243635 | 0.04541708 |
| UVM | Endothelial cell | 0.338604 | 0.00212489 |
| UVM | Macrophage M1 | 0.22944018 | 0.04063105 |
| UVM | Mast cell | -0.3451672 | 0.00171474 |
| UVM | Plasmacytoid dendritic cell | -0.2651357 | 0.01746216 |
| UVM | T cell regulatory (Tregs) | -0.2938813 | 0.00814775 |
| UVM | stroma score | 0.35042194 | 0.00152986 |

**Supplementary Table 2.** The results of the correlation of SLC2A1 expression with immune checkpoint genes in pan-cancer (only terms of pvalue<0.05 were displayed).

| cancer | genes | cor | pvalue |
| --- | --- | --- | --- |
| ACC | CTLA4 | -0.3179323 | 0.00429979 |
| ACC | HAVCR2 | -0.3781402 | 0.00064376 |
| ACC | PDCD1LG2 | -0.3212775 | 0.00388911 |
| ACC | TIGIT | -0.2433125 | 0.03071677 |
| BLCA | CTLA4 | -0.1452065 | 0.00328612 |
| BLCA | HAVCR2 | -0.1577033 | 0.00139493 |
| BLCA | LAG3 | -0.1608105 | 0.00113069 |
| BLCA | PDCD1 | -0.1688225 | 0.00061663 |
| BLCA | SIGLEC15 | -0.1463867 | 0.00303889 |
| BLCA | TIGIT | -0.1649248 | 0.00082572 |
| BRCA | HAVCR2 | -0.0793227 | 0.00836956 |
| BRCA | SIGLEC15 | -0.1822272 | 1.07E-09 |
| CESC | CD274 | 0.28412298 | 4.13E-07 |
| CESC | PDCD1LG2 | 0.11835233 | 0.03821599 |
| CESC | SIGLEC15 | -0.2231562 | 8.02E-05 |
| ESCA | CD274 | 0.25927182 | 0.00038169 |
| ESCA | CTLA4 | -0.1862989 | 0.01120919 |
| ESCA | PDCD1 | -0.2002502 | 0.00635425 |
| ESCA | PDCD1LG2 | 0.15892309 | 0.03081467 |
| ESCA | SIGLEC15 | -0.4555781 | 7.23E-11 |
| ESCA | TIGIT | -0.1504549 | 0.04101744 |
| GBM | CD274 | 0.2447798 | 0.00147374 |
| GBM | CTLA4 | -0.1585741 | 0.04067704 |
| GBM | LAG3 | -0.2489049 | 0.00121763 |
| HNSC | CTLA4 | -0.3309034 | 8.37E-15 |
| HNSC | HAVCR2 | -0.3118855 | 4.06E-13 |
| HNSC | LAG3 | -0.32725 | 1.71E-14 |
| HNSC | PDCD1 | -0.3495218 | 1.91E-16 |
| HNSC | PDCD1LG2 | -0.0963878 | 0.02769517 |
| HNSC | SIGLEC15 | -0.1860884 | 1.88E-05 |
| HNSC | TIGIT | -0.319676 | 9.72E-14 |
| KICH | CD274 | 0.30788018 | 0.01219328 |
| KICH | SIGLEC15 | 0.3453293 | 0.00471557 |
| KIRC | CD274 | -0.1359083 | 0.00165842 |
| KIRP | CD274 | 0.24091571 | 3.46E-05 |
| KIRP | PDCD1LG2 | 0.18371119 | 0.00164832 |
| KIRP | TIGIT | 0.12411373 | 0.03431944 |
| LGG | CD274 | 0.09610286 | 0.02694228 |
| LGG | LAG3 | -0.1174455 | 0.00679379 |
| LIHC | CD274 | 0.24644334 | 1.45E-06 |
| LIHC | CTLA4 | 0.23824 | 3.27E-06 |
| LIHC | HAVCR2 | 0.36318568 | 6.19E-13 |
| LIHC | PDCD1 | 0.19904346 | 0.00010881 |
| LIHC | PDCD1LG2 | 0.17228428 | 0.00083414 |
| LIHC | SIGLEC15 | -0.1624738 | 0.00164221 |
| LIHC | TIGIT | 0.20199854 | 8.54E-05 |
| LUAD | CD274 | 0.24738935 | 1.20E-08 |
| LUAD | LAG3 | 0.10077471 | 0.02192477 |
| LUAD | PDCD1LG2 | 0.17468043 | 6.53E-05 |
| LUAD | SIGLEC15 | 0.19588029 | 7.24E-06 |
| LUSC | CTLA4 | -0.4072889 | 1.76E-21 |
| LUSC | HAVCR2 | -0.3226111 | 1.27E-13 |
| LUSC | LAG3 | -0.3980748 | 1.64E-20 |
| LUSC | PDCD1 | -0.4486965 | 3.08E-26 |
| LUSC | PDCD1LG2 | -0.1818647 | 4.15E-05 |
| LUSC | SIGLEC15 | -0.1969395 | 8.78E-06 |
| LUSC | TIGIT | -0.3732673 | 4.83E-18 |
| MESO | CD274 | 0.22548662 | 0.03594287 |
| OV | HAVCR2 | -0.1513079 | 0.00786448 |
| OV | SIGLEC15 | 0.1837462 | 0.00119847 |
| PAAD | CTLA4 | -0.1774508 | 0.01748308 |
| PAAD | LAG3 | -0.2341766 | 0.00164592 |
| PAAD | PDCD1 | -0.2534555 | 0.000642 |
| PAAD | TIGIT | -0.200318 | 0.00725963 |
| PCPG | CD274 | -0.1772399 | 0.01618875 |
| PCPG | HAVCR2 | 0.175327 | 0.01738951 |
| PCPG | PDCD1LG2 | 0.33802662 | 3.10E-06 |
| PRAD | CD274 | 0.09661678 | 0.03110561 |
| PRAD | CTLA4 | -0.1358425 | 0.00238214 |
| PRAD | HAVCR2 | -0.1060309 | 0.01793751 |
| PRAD | LAG3 | -0.1010285 | 0.02415541 |
| PRAD | PDCD1 | -0.1169227 | 0.00901085 |
| PRAD | PDCD1LG2 | -0.13352 | 0.00283104 |
| PRAD | TIGIT | -0.1107027 | 0.01344251 |
| READ | TIGIT | -0.2047619 | 0.0092746 |
| SARC | CD274 | 0.22570183 | 0.00022375 |
| SARC | PDCD1 | -0.1368488 | 0.02647357 |
| SARC | SIGLEC15 | 0.29280168 | 1.35E-06 |
| SKCM | CD274 | -0.1709084 | 0.00018795 |
| SKCM | HAVCR2 | -0.1503006 | 0.00105445 |
| SKCM | LAG3 | -0.1626733 | 0.00038832 |
| SKCM | PDCD1 | -0.1742732 | 0.0001425 |
| SKCM | PDCD1LG2 | -0.2165199 | 2.12E-06 |
| SKCM | SIGLEC15 | 0.22125064 | 1.18E-06 |
| SKCM | TIGIT | -0.2134637 | 2.81E-06 |
| STAD | CTLA4 | -0.1296378 | 0.00819028 |
| STAD | HAVCR2 | -0.1936126 | 7.19E-05 |
| STAD | LAG3 | -0.1409696 | 0.00400854 |
| STAD | PDCD1 | -0.2008755 | 3.76E-05 |
| STAD | PDCD1LG2 | -0.1856633 | 0.0001424 |
| STAD | TIGIT | -0.2588895 | 8.82E-08 |
| TGCT | CD274 | -0.2582763 | 0.00117167 |
| TGCT | CTLA4 | -0.4233251 | 5.02E-08 |
| TGCT | HAVCR2 | -0.1691233 | 0.0349165 |
| TGCT | LAG3 | -0.3723728 | 2.03E-06 |
| TGCT | PDCD1 | -0.4417379 | 1.13E-08 |
| TGCT | PDCD1LG2 | -0.3371845 | 1.88E-05 |
| TGCT | SIGLEC15 | -0.3911207 | 4.46E-07 |
| TGCT | TIGIT | -0.38392 | 9.22E-07 |
| THCA | CD274 | 0.28589547 | 4.17E-11 |
| THCA | CTLA4 | 0.17488532 | 6.83E-05 |
| THCA | HAVCR2 | 0.26280658 | 1.50E-09 |
| THCA | PDCD1LG2 | 0.1724764 | 8.62E-05 |
| THCA | SIGLEC15 | 0.4538034 | 2.00E-27 |
| THCA | TIGIT | 0.17485339 | 6.85E-05 |
| THYM | CD274 | 0.32257796 | 0.00034988 |
| THYM | CTLA4 | 0.30040975 | 0.00090025 |
| THYM | HAVCR2 | 0.18778387 | 0.04012507 |
| THYM | LAG3 | 0.19146469 | 0.0363258 |
| THYM | PDCD1LG2 | 0.43330787 | 9.99E-07 |
| THYM | SIGLEC15 | -0.2819541 | 0.00180927 |
| THYM | TIGIT | 0.26699076 | 0.00329066 |
| UCEC | CD274 | 0.11120469 | 0.01019026 |
| UCEC | CTLA4 | -0.2180662 | 3.70E-07 |
| UCEC | HAVCR2 | -0.130627 | 0.00251413 |
| UCEC | LAG3 | -0.1307011 | 0.00249981 |
| UCEC | PDCD1 | -0.1874608 | 1.32E-05 |
| UCEC | SIGLEC15 | 0.18566087 | 1.60E-05 |
| UCEC | TIGIT | -0.1354695 | 0.00172044 |
| UCS | PDCD1LG2 | -0.359217 | 0.00633445 |
| UCS | SIGLEC15 | 0.51411624 | 4.30E-05 |

**Supplementary Table 3.** Correlation between SLC2A1 expression and different tumor functional status in different cancers (only terms of pvalue<0.05 were displayed).

| Cancer | group | cor | pvalue |
| --- | --- | --- | --- |
| AML | Hypoxia | 0.558 | 0.025 |
| AST | Metastasis | 0.113 | 0 |
| AST | Hypoxia | 0.114 | 0 |
| BRCA | Quiescence | -0.549 | 0.024 |
| BRCA | DNArepair | -0.211 | 0 |
| BRCA | Inflammation | -0.17 | 0.001 |
| BRCA | CellCycle | -0.16 | 0.002 |
| BRCA | EMT | 0.145 | 0.016 |
| BRCA | Invasion | 0.158 | 0.002 |
| BRCA | Invasion | 0.159 | 0.008 |
| BRCA | Inflammation | 0.172 | 0.004 |
| BRCA | Stemness | 0.176 | 0.001 |
| BRCA | Angiogenesis | 0.198 | 0.001 |
| BRCA | Angiogenesis | 0.207 | 0 |
| BRCA | Apoptosis | 0.227 | 0 |
| BRCA | EMT | 0.239 | 0 |
| BRCA | Differentiation | 0.242 | 0 |
| BRCA | Hypoxia | 0.264 | 0 |
| BRCA | Quiescence | 0.275 | 0 |
| BRCA | Metastasis | 0.279 | 0 |
| BRCA | Metastasis | 0.553 | 0 |
| BRCA | Hypoxia | 0.631 | 0 |
| CML | Inflammation | 0.138 | 0.016 |
| CML | Hypoxia | 0.157 | 0.006 |
| CML | Apoptosis | 0.162 | 0.005 |
| CML | Differentiation | 0.175 | 0.002 |
| CRC | DNArepair | -0.362 | 0 |
| CRC | CellCycle | -0.322 | 0 |
| CRC | Invasion | -0.308 | 0.001 |
| CRC | Quiescence | 0.186 | 0.045 |
| CRC | Inflammation | 0.238 | 0.01 |
| CRC | Angiogenesis | 0.24 | 0.009 |
| CRC | Metastasis | 0.406 | 0 |
| CRC | Hypoxia | 0.41 | 0 |
| GBM | Stemness | -0.134 | 0.005 |
| GBM | Stemness | -0.121 | 0.019 |
| GBM | Invasion | -0.107 | 0.027 |
| GBM | Metastasis | 0.096 | 0.045 |
| GBM | Invasion | 0.126 | 0.014 |
| GBM | Inflammation | 0.133 | 0.006 |
| GBM | Angiogenesis | 0.162 | 0.001 |
| GBM | Metastasis | 0.164 | 0.001 |
| GBM | Angiogenesis | 0.206 | 0 |
| GBM | Hypoxia | 0.288 | 0 |
| GBM | Hypoxia | 0.452 | 0 |
| Glioma | DNAdamage | -0.071 | 0.038 |
| Glioma | Apoptosis | -0.07 | 0.038 |
| Glioma | Proliferation | 0.069 | 0.041 |
| Glioma | CellCycle | 0.072 | 0.033 |
| Glioma | EMT | 0.088 | 0.009 |
| Glioma | Hypoxia | 0.105 | 0.002 |
| Glioma | Differentiation | 0.116 | 0.001 |
| Glioma | Metastasis | 0.116 | 0.001 |
| HGG | DNArepair | -0.152 | 0 |
| HGG | Differentiation | 0.095 | 0.01 |
| HGG | Angiogenesis | 0.104 | 0.005 |
| HGG | Hypoxia | 0.104 | 0.005 |
| HGG | CellCycle | 0.108 | 0.003 |
| HGG | Apoptosis | 0.172 | 0 |
| HGG | EMT | 0.182 | 0 |
| HGG | Metastasis | 0.28 | 0 |
| HNSCC | DNArepair | -0.209 | 0 |
| HNSCC | CellCycle | -0.163 | 0 |
| HNSCC | DNAdamage | -0.154 | 0 |
| HNSCC | Inflammation | 0.119 | 0 |
| HNSCC | Differentiation | 0.126 | 0 |
| HNSCC | Apoptosis | 0.168 | 0 |
| HNSCC | Angiogenesis | 0.181 | 0 |
| HNSCC | Metastasis | 0.358 | 0 |
| HNSCC | Hypoxia | 0.501 | 0 |
| LUAD | Metastasis | 0.187 | 0.042 |
| LUAD | Stemness | 0.372 | 0.047 |
| LUAD | Proliferation | 0.394 | 0.035 |
| MEL | Invasion | -0.114 | 0.008 |
| MEL | Hypoxia | 0.256 | 0 |
| NSCLC | DNArepair | -0.299 | 0 |
| NSCLC | DNAdamage | -0.289 | 0 |
| NSCLC | Stemness | -0.188 | 0 |
| NSCLC | Proliferation | -0.156 | 0 |
| NSCLC | Invasion | -0.136 | 0 |
| NSCLC | CellCycle | -0.115 | 0 |
| NSCLC | Apoptosis | -0.084 | 0 |
| NSCLC | Differentiation | 0.042 | 0.048 |
| NSCLC | Inflammation | 0.115 | 0 |
| NSCLC | Angiogenesis | 0.255 | 0 |
| NSCLC | EMT | 0.316 | 0 |
| NSCLC | Metastasis | 0.392 | 0 |
| NSCLC | Hypoxia | 0.416 | 0 |
| ODG | DNAdamage | -0.1 | 0.018 |
| ODG | Apoptosis | -0.093 | 0.029 |
| ODG | Inflammation | 0.103 | 0.015 |
| ODG | Stemness | 0.14 | 0.001 |
| ODG | Metastasis | 0.188 | 0 |
| OV | Invasion | -0.434 | 0 |
| OV | Stemness | -0.269 | 0.005 |
| OV | DNArepair | -0.206 | 0.035 |
| OV | EMT | 0.223 | 0.021 |
| OV | Apoptosis | 0.308 | 0.001 |
| OV | Metastasis | 0.395 | 0 |
| OV | Hypoxia | 0.527 | 0 |
| RB | DNArepair | -0.497 | 0 |
| RB | CellCycle | -0.429 | 0 |
| RB | DNAdamage | -0.346 | 0 |
| RB | Invasion | -0.133 | 0 |
| RB | Apoptosis | -0.073 | 0 |
| RB | EMT | 0.085 | 0 |
| RB | Hypoxia | 0.139 | 0 |
| RB | Stemness | 0.212 | 0 |
| RB | Quiescence | 0.257 | 0 |
| RB | Metastasis | 0.311 | 0 |
| RB | Inflammation | 0.37 | 0 |
| RB | Differentiation | 0.497 | 0 |
| RB | Angiogenesis | 0.548 | 0 |
| RCC | DNArepair | -0.367 | 0.001 |
| RCC | Inflammation | -0.36 | 0.001 |
| RCC | CellCycle | -0.274 | 0.013 |
| RCC | Stemness | 0.345 | 0.002 |
| RCC | Angiogenesis | 0.407 | 0 |
| RCC | Stemness | 0.421 | 0.012 |
| RCC | Differentiation | 0.468 | 0 |
| RCC | Hypoxia | 0.505 | 0.002 |
| RCC | Hypoxia | 0.562 | 0 |
| UM | DNArepair | -0.491 | 0 |
| UM | DNAdamage | -0.424 | 0 |
| UM | Apoptosis | -0.358 | 0 |
| UM | Invasion | -0.297 | 0 |
| UM | Metastasis | -0.247 | 0 |
| UM | Quiescence | -0.203 | 0 |
| UM | Inflammation | -0.187 | 0 |
| UM | EMT | -0.15 | 0 |
| UM | Differentiation | -0.146 | 0 |
| UM | Proliferation | -0.118 | 0 |
| UM | Angiogenesis | -0.048 | 0.002 |
| UM | Stemness | 0.044 | 0.005 |
| UM | Hypoxia | 0.245 | 0 |

**Supplementary Table 4.** The results of GSEA analysis of HALLMARK betweenSLC2A1 low expression group and high expression group (only terms of |NES| >2 were displayed).

| Tumor | Term | NES | FDR |
| --- | --- | --- | --- |
| ACC | HALLMARK_ALLOGRAFT_REJECTION | -2.6988669 | 1.48E-09 |
| ACC | HALLMARK_G2M_CHECKPOINT | 2.66572319 | 1.48E-09 |
| ACC | HALLMARK_E2F_TARGETS | 2.50579697 | 1.48E-09 |
| ACC | HALLMARK_INTERFERON_GAMMA_RESPONSE | -2.0809703 | 1.48E-09 |
| BLCA | HALLMARK_P53_PATHWAY | 2.30938329 | 2.50E-09 |
| BRCA | HALLMARK_E2F_TARGETS | 3.21924184 | 8.33E-10 |
| BRCA | HALLMARK_G2M_CHECKPOINT | 3.09728475 | 8.33E-10 |
| BRCA | HALLMARK_MYC_TARGETS_V1 | 2.77172585 | 8.33E-10 |
| BRCA | HALLMARK_MTORC1_SIGNALING | 2.48770662 | 8.33E-10 |
| BRCA | HALLMARK_GLYCOLYSIS | 2.09959726 | 8.33E-10 |
| BRCA | HALLMARK_MITOTIC_SPINDLE | 2.09564914 | 8.33E-10 |
| BRCA | HALLMARK_MYC_TARGETS_V2 | 2.56263286 | 9.14E-10 |
| CESC | HALLMARK_P53_PATHWAY | 2.35872765 | 2.50E-09 |
| CESC | HALLMARK_TNFA_SIGNALING_VIA_NFKB | 2.2757049 | 2.50E-09 |
| CHOL | HALLMARK_XENOBIOTIC_METABOLISM | -2.6456661 | 1.25E-09 |
| CHOL | HALLMARK_BILE_ACID_METABOLISM | -2.51633 | 1.25E-09 |
| CHOL | HALLMARK_OXIDATIVE_PHOSPHORYLATION | -2.474606 | 1.25E-09 |
| CHOL | HALLMARK_COAGULATION | -2.3277473 | 1.25E-09 |
| CHOL | HALLMARK_FATTY_ACID_METABOLISM | -2.1144379 | 3.94E-08 |
| COAD | HALLMARK_EPITHELIAL_MESENCHYMAL_TRANSITION | 2.54771872 | 1.67E-09 |
| COAD | HALLMARK_TNFA_SIGNALING_VIA_NFKB | 2.44906597 | 1.67E-09 |
| COAD | HALLMARK_HYPOXIA | 2.16387352 | 1.67E-09 |
| COAD | HALLMARK_APICAL_JUNCTION | 2.11740909 | 2.49E-09 |
| COAD | HALLMARK_INFLAMMATORY_RESPONSE | 2.09353989 | 2.49E-09 |
| GBM | HALLMARK_EPITHELIAL_MESENCHYMAL_TRANSITION | 3.07284761 | 3.85E-10 |
| GBM | HALLMARK_HYPOXIA | 2.96296075 | 3.85E-10 |
| GBM | HALLMARK_TNFA_SIGNALING_VIA_NFKB | 2.90649377 | 3.85E-10 |
| GBM | HALLMARK_INFLAMMATORY_RESPONSE | 2.55751926 | 3.85E-10 |
| GBM | HALLMARK_GLYCOLYSIS | 2.45043311 | 3.85E-10 |
| GBM | HALLMARK_COAGULATION | 2.446494 | 3.85E-10 |
| GBM | HALLMARK_APOPTOSIS | 2.39999073 | 3.85E-10 |
| GBM | HALLMARK_P53_PATHWAY | 2.33360702 | 3.85E-10 |
| GBM | HALLMARK_MTORC1_SIGNALING | 2.28408864 | 3.85E-10 |
| GBM | HALLMARK_UV_RESPONSE_DN | 2.22748175 | 3.85E-10 |
| GBM | HALLMARK_KRAS_SIGNALING_UP | 2.22064292 | 3.85E-10 |
| GBM | HALLMARK_IL2_STAT5_SIGNALING | 2.21945537 | 3.85E-10 |
| GBM | HALLMARK_ESTROGEN_RESPONSE_EARLY | 2.12207684 | 3.85E-10 |
| GBM | HALLMARK_APICAL_JUNCTION | 2.09655409 | 5.27E-10 |
| GBM | HALLMARK_COMPLEMENT | 2.07524409 | 1.49E-09 |
| GBM | HALLMARK_ESTROGEN_RESPONSE_LATE | 2.05306329 | 1.89E-09 |
| GBM | HALLMARK_IL6_JAK_STAT3_SIGNALING | 2.2197518 | 1.33E-08 |
| GBM | HALLMARK_ANGIOGENESIS | 2.19549714 | 7.61E-07 |
| GBM | HALLMARK_ANDROGEN_RESPONSE | 2.01353545 | 2.61E-06 |
| HNSC | HALLMARK_HYPOXIA | 2.08271108 | 1.67E-09 |
| HNSC | HALLMARK_P53_PATHWAY | 2.06732988 | 1.67E-09 |
| HNSC | HALLMARK_ALLOGRAFT_REJECTION | -2.0633167 | 1.67E-09 |
| KICH | HALLMARK_E2F_TARGETS | 2.62200723 | 1.67E-09 |
| KICH | HALLMARK_G2M_CHECKPOINT | 2.44863131 | 1.67E-09 |
| KICH | HALLMARK_MTORC1_SIGNALING | 2.09293506 | 1.67E-09 |
| KICH | HALLMARK_EPITHELIAL_MESENCHYMAL_TRANSITION | 2.00105118 | 3.81E-08 |
| KIRC | HALLMARK_EPITHELIAL_MESENCHYMAL_TRANSITION | 2.77583775 | 8.33E-10 |
| KIRC | HALLMARK_OXIDATIVE_PHOSPHORYLATION | -2.4126557 | 8.33E-10 |
| KIRC | HALLMARK_TNFA_SIGNALING_VIA_NFKB | 2.30721552 | 8.33E-10 |
| KIRC | HALLMARK_HYPOXIA | 2.28098877 | 8.33E-10 |
| KIRC | HALLMARK_INFLAMMATORY_RESPONSE | 2.04006634 | 8.33E-10 |
| KIRC | HALLMARK_GLYCOLYSIS | 2.02699622 | 8.33E-10 |
| KIRC | HALLMARK_UV_RESPONSE_DN | 2.06138555 | 6.16E-09 |
| KIRC | HALLMARK_FATTY_ACID_METABOLISM | -2.0965389 | 1.48E-08 |
| KIRP | HALLMARK_G2M_CHECKPOINT | 2.53525967 | 7.14E-10 |
| KIRP | HALLMARK_EPITHELIAL_MESENCHYMAL_TRANSITION | 2.43989203 | 7.14E-10 |
| KIRP | HALLMARK_MTORC1_SIGNALING | 2.27949425 | 7.14E-10 |
| KIRP | HALLMARK_E2F_TARGETS | 2.24011896 | 7.14E-10 |
| KIRP | HALLMARK_HYPOXIA | 2.16928925 | 7.14E-10 |
| KIRP | HALLMARK_TNFA_SIGNALING_VIA_NFKB | 2.145456 | 7.14E-10 |
| KIRP | HALLMARK_GLYCOLYSIS | 2.11608779 | 7.14E-10 |
| KIRP | HALLMARK_MYC_TARGETS_V1 | 2.04133707 | 1.06E-08 |
| LGG | HALLMARK_E2F_TARGETS | -2.6020436 | 1.67E-09 |
| LGG | HALLMARK_G2M_CHECKPOINT | -2.2837568 | 1.67E-09 |
| LGG | HALLMARK_EPITHELIAL_MESENCHYMAL_TRANSITION | 2.06125796 | 1.67E-09 |
| LIHC | HALLMARK_BILE_ACID_METABOLISM | -2.7257466 | 8.33E-10 |
| LIHC | HALLMARK_XENOBIOTIC_METABOLISM | -2.4062737 | 8.33E-10 |
| LIHC | HALLMARK_FATTY_ACID_METABOLISM | -2.3259332 | 8.33E-10 |
| LIHC | HALLMARK_COAGULATION | -2.0689326 | 6.16E-08 |
| LUAD | HALLMARK_G2M_CHECKPOINT | 2.89875928 | 7.14E-10 |
| LUAD | HALLMARK_E2F_TARGETS | 2.89135297 | 7.14E-10 |
| LUAD | HALLMARK_EPITHELIAL_MESENCHYMAL_TRANSITION | 2.42294685 | 7.14E-10 |
| LUAD | HALLMARK_MYC_TARGETS_V1 | 2.41982539 | 7.14E-10 |
| LUAD | HALLMARK_MTORC1_SIGNALING | 2.35667988 | 7.14E-10 |
| LUAD | HALLMARK_MITOTIC_SPINDLE | 2.23383442 | 7.14E-10 |
| LUAD | HALLMARK_GLYCOLYSIS | 2.21895283 | 7.14E-10 |
| LUAD | HALLMARK_HYPOXIA | 2.0683334 | 3.19E-09 |
| LUAD | HALLMARK_MYC_TARGETS_V2 | 2.01185873 | 8.78E-05 |
| LUSC | HALLMARK_ALLOGRAFT_REJECTION | -2.396433 | 8.33E-10 |
| LUSC | HALLMARK_HYPOXIA | 2.185968 | 8.33E-10 |
| LUSC | HALLMARK_INTERFERON_GAMMA_RESPONSE | -2.178342 | 8.33E-10 |
| LUSC | HALLMARK_GLYCOLYSIS | 2.17207594 | 8.33E-10 |
| LUSC | HALLMARK_MTORC1_SIGNALING | 2.16560745 | 8.33E-10 |
| LUSC | HALLMARK_P53_PATHWAY | 2.13849111 | 8.33E-10 |
| LUSC | HALLMARK_INTERFERON_ALPHA_RESPONSE | -2.0718997 | 2.00E-08 |
| MESO | HALLMARK_G2M_CHECKPOINT | 2.84395172 | 7.14E-10 |
| MESO | HALLMARK_E2F_TARGETS | 2.82358457 | 7.14E-10 |
| MESO | HALLMARK_EPITHELIAL_MESENCHYMAL_TRANSITION | 2.5666257 | 7.14E-10 |
| MESO | HALLMARK_MTORC1_SIGNALING | 2.52303425 | 7.14E-10 |
| MESO | HALLMARK_MITOTIC_SPINDLE | 2.33211647 | 7.14E-10 |
| MESO | HALLMARK_GLYCOLYSIS | 2.22310148 | 7.14E-10 |
| MESO | HALLMARK_INTERFERON_GAMMA_RESPONSE | 2.13542858 | 7.14E-10 |
| MESO | HALLMARK_HYPOXIA | 2.00324986 | 6.69E-09 |
| MESO | HALLMARK_INFLAMMATORY_RESPONSE | 2.00879888 | 1.82E-08 |
| MESO | HALLMARK_INTERFERON_ALPHA_RESPONSE | 2.06179227 | 4.70E-06 |
| OV | HALLMARK_INTERFERON_GAMMA_RESPONSE | -2.4534415 | 8.33E-10 |
| OV | HALLMARK_ALLOGRAFT_REJECTION | -2.4324014 | 8.33E-10 |
| OV | HALLMARK_INTERFERON_ALPHA_RESPONSE | -2.4295437 | 8.33E-10 |
| OV | HALLMARK_EPITHELIAL_MESENCHYMAL_TRANSITION | 2.37308355 | 8.33E-10 |
| OV | HALLMARK_HYPOXIA | 2.3303526 | 8.33E-10 |
| OV | HALLMARK_GLYCOLYSIS | 2.1358848 | 8.33E-10 |
| OV | HALLMARK_MTORC1_SIGNALING | 2.04885471 | 1.28E-08 |
| PAAD | HALLMARK_G2M_CHECKPOINT | 2.53487121 | 1.00E-09 |
| PAAD | HALLMARK_E2F_TARGETS | 2.44696226 | 1.00E-09 |
| PAAD | HALLMARK_TNFA_SIGNALING_VIA_NFKB | 2.23757476 | 1.00E-09 |
| PAAD | HALLMARK_GLYCOLYSIS | 2.23511794 | 1.00E-09 |
| PAAD | HALLMARK_MITOTIC_SPINDLE | 2.2045821 | 1.00E-09 |
| PAAD | HALLMARK_HYPOXIA | 2.05917451 | 1.65E-09 |
| PAAD | HALLMARK_MTORC1_SIGNALING | 2.03831284 | 2.35E-09 |
| PAAD | HALLMARK_P53_PATHWAY | 2.02833856 | 3.82E-09 |
| PAAD | HALLMARK_INTERFERON_ALPHA_RESPONSE | 2.2764571 | 1.96E-08 |
| PAAD | HALLMARK_PANCREAS_BETA_CELLS | -2.074161 | 1.70E-06 |
| PCPG | HALLMARK_EPITHELIAL_MESENCHYMAL_TRANSITION | 2.38728569 | 1.00E-09 |
| PCPG | HALLMARK_TNFA_SIGNALING_VIA_NFKB | 2.28810646 | 1.00E-09 |
| PCPG | HALLMARK_HYPOXIA | 2.21326277 | 1.00E-09 |
| PCPG | HALLMARK_INFLAMMATORY_RESPONSE | 2.01717252 | 1.00E-09 |
| PCPG | HALLMARK_KRAS_SIGNALING_UP | 2.0095899 | 1.00E-09 |
| PRAD | HALLMARK_ANDROGEN_RESPONSE | 2.65633554 | 1.25E-09 |
| PRAD | HALLMARK_EPITHELIAL_MESENCHYMAL_TRANSITION | -2.2329333 | 1.25E-09 |
| PRAD | HALLMARK_MTORC1_SIGNALING | 2.22604468 | 1.25E-09 |
| PRAD | HALLMARK_G2M_CHECKPOINT | 2.22206801 | 1.25E-09 |
| READ | HALLMARK_EPITHELIAL_MESENCHYMAL_TRANSITION | 2.1727115 | 6.05E-09 |
| READ | HALLMARK_INTERFERON_ALPHA_RESPONSE | -2.0559756 | 2.37E-05 |
| SKCM | HALLMARK_ALLOGRAFT_REJECTION | -2.7988719 | 1.00E-09 |
| SKCM | HALLMARK_INTERFERON_GAMMA_RESPONSE | -2.7332269 | 1.00E-09 |
| SKCM | HALLMARK_INTERFERON_ALPHA_RESPONSE | -2.5373207 | 1.00E-09 |
| SKCM | HALLMARK_GLYCOLYSIS | 2.23458044 | 1.00E-09 |
| SKCM | HALLMARK_HYPOXIA | 2.22992396 | 1.00E-09 |
| SKCM | HALLMARK_ESTROGEN_RESPONSE_LATE | 2.06084164 | 7.15E-09 |
| SKCM | HALLMARK_ESTROGEN_RESPONSE_EARLY | 2.01683117 | 5.16E-08 |
| SKCM | HALLMARK_KRAS_SIGNALING_DN | 2.03548109 | 6.75E-08 |
| STAD | HALLMARK_E2F_TARGETS | 2.94559868 | 6.25E-10 |
| STAD | HALLMARK_MYC_TARGETS_V2 | 2.61574028 | 6.25E-10 |
| STAD | HALLMARK_G2M_CHECKPOINT | 2.56635378 | 6.25E-10 |
| STAD | HALLMARK_MYC_TARGETS_V1 | 2.52746323 | 6.25E-10 |
| STAD | HALLMARK_MTORC1_SIGNALING | 2.51214448 | 6.25E-10 |
| STAD | HALLMARK_GLYCOLYSIS | 2.3537009 | 6.25E-10 |
| STAD | HALLMARK_ESTROGEN_RESPONSE_LATE | 2.18650806 | 6.25E-10 |
| STAD | HALLMARK_ALLOGRAFT_REJECTION | -2.1017893 | 6.25E-10 |
| STAD | HALLMARK_CHOLESTEROL_HOMEOSTASIS | 2.09751349 | 2.54E-05 |
| THCA | HALLMARK_OXIDATIVE_PHOSPHORYLATION | -2.3136602 | 7.14E-10 |
| THCA | HALLMARK_TNFA_SIGNALING_VIA_NFKB | 2.22839543 | 7.14E-10 |
| THCA | HALLMARK_INFLAMMATORY_RESPONSE | 2.21258024 | 7.14E-10 |
| THCA | HALLMARK_EPITHELIAL_MESENCHYMAL_TRANSITION | 2.19729958 | 7.14E-10 |
| THCA | HALLMARK_ALLOGRAFT_REJECTION | 2.15438008 | 7.14E-10 |
| THCA | HALLMARK_INTERFERON_GAMMA_RESPONSE | 2.12884249 | 7.14E-10 |
| THCA | HALLMARK_KRAS_SIGNALING_UP | 2.01961813 | 7.14E-10 |
| THCA | HALLMARK_COAGULATION | 2.03452128 | 2.31E-09 |
| UCEC | HALLMARK_G2M_CHECKPOINT | 2.9204232 | 8.33E-10 |
| UCEC | HALLMARK_HYPOXIA | 2.85686291 | 8.33E-10 |
| UCEC | HALLMARK_E2F_TARGETS | 2.79461075 | 8.33E-10 |
| UCEC | HALLMARK_MTORC1_SIGNALING | 2.55259785 | 8.33E-10 |
| UCEC | HALLMARK_TNFA_SIGNALING_VIA_NFKB | 2.55074405 | 8.33E-10 |
| UCEC | HALLMARK_MITOTIC_SPINDLE | 2.27451838 | 8.33E-10 |
| UCEC | HALLMARK_GLYCOLYSIS | 2.13548573 | 1.48E-09 |
| UCS | HALLMARK_MYOGENESIS | -3.0116688 | 1.67E-09 |
| UCS | HALLMARK_ALLOGRAFT_REJECTION | -2.1270587 | 1.67E-09 |
| UCS | HALLMARK_ESTROGEN_RESPONSE_LATE | 2.09901663 | 1.67E-09 |
| UVM | HALLMARK_EPITHELIAL_MESENCHYMAL_TRANSITION | 2.39206574 | 1.67E-09 |
| UVM | HALLMARK_HYPOXIA | 2.19600427 | 1.67E-09 |
| UVM | HALLMARK_MYOGENESIS | 2.17685004 | 1.67E-09 |
| UVM | HALLMARK_COAGULATION | 2.23024963 | 7.44E-09 |

**Supplementary Table 5.** The GO enrichment analysis for 51 genes contained in PPI network (only the TOP250 terms were displayed).

| Term ID | | Type | Description | GeneRatio | p.adjust |
| --- | --- | --- | --- | --- | --- |
| GO:0006090 | BP | | pyruvate metabolic process | 15/51 | 2.46E-19 |
| GO:0006096 | BP | | glycolytic process | 14/51 | 2.46E-19 |
| GO:0006757 | BP | | ATP generation from ADP | 14/51 | 2.46E-19 |
| GO:0046031 | BP | | ADP metabolic process | 14/51 | 7.46E-19 |
| GO:0006165 | BP | | nucleoside diphosphate phosphorylation | 14/51 | 2.39E-18 |
| GO:0046939 | BP | | nucleotide phosphorylation | 14/51 | 2.39E-18 |
| GO:0009135 | BP | | purine nucleoside diphosphate metabolic process | 14/51 | 2.77E-18 |
| GO:0009179 | BP | | purine ribonucleoside diphosphate metabolic process | 14/51 | 2.77E-18 |
| GO:0009185 | BP | | ribonucleoside diphosphate metabolic process | 14/51 | 8.47E-18 |
| GO:0016052 | BP | | carbohydrate catabolic process | 15/51 | 1.17E-17 |
| GO:0009132 | BP | | nucleoside diphosphate metabolic process | 14/51 | 6.10E-17 |
| GO:0005996 | BP | | monosaccharide metabolic process | 16/51 | 1.08E-15 |
| GO:0019318 | BP | | hexose metabolic process | 15/51 | 1.07E-14 |
| GO:0046323 | BP | | glucose import | 11/51 | 2.40E-14 |
| GO:1904659 | BP | | glucose transmembrane transport | 12/51 | 2.46E-14 |
| GO:0008645 | BP | | hexose transmembrane transport | 12/51 | 3.54E-14 |
| GO:0015749 | BP | | monosaccharide transmembrane transport | 12/51 | 4.11E-14 |
| GO:0006091 | BP | | generation of precursor metabolites and energy | 18/51 | 4.22E-14 |
| GO:0046034 | BP | | ATP metabolic process | 15/51 | 4.45E-14 |
| GO:0034219 | BP | | carbohydrate transmembrane transport | 12/51 | 1.14E-13 |
| GO:0036293 | BP | | response to decreased oxygen levels | 15/51 | 1.56E-13 |
| GO:0070482 | BP | | response to oxygen levels | 15/51 | 4.87E-13 |
| GO:0006006 | BP | | glucose metabolic process | 13/51 | 5.41E-13 |
| GO:0008643 | BP | | carbohydrate transport | 12/51 | 8.03E-13 |
| GO:0001666 | BP | | response to hypoxia | 14/51 | 1.87E-12 |
| GO:0009150 | BP | | purine ribonucleotide metabolic process | 15/51 | 2.47E-12 |
| GO:0009259 | BP | | ribonucleotide metabolic process | 15/51 | 4.79E-12 |
| GO:0006163 | BP | | purine nucleotide metabolic process | 15/51 | 6.49E-12 |
| GO:0019693 | BP | | ribose phosphate metabolic process | 15/51 | 6.49E-12 |
| GO:0072521 | BP | | purine-containing compound metabolic process | 15/51 | 1.38E-11 |
| GO:0036294 | BP | | cellular response to decreased oxygen levels | 11/51 | 1.67E-11 |
| GO:0071453 | BP | | cellular response to oxygen levels | 11/51 | 4.62E-11 |
| GO:0009117 | BP | | nucleotide metabolic process | 15/51 | 1.22E-10 |
| GO:0006753 | BP | | nucleoside phosphate metabolic process | 15/51 | 1.49E-10 |
| GO:0071456 | BP | | cellular response to hypoxia | 10/51 | 3.05E-10 |
| GO:0006007 | BP | | glucose catabolic process | 6/51 | 7.11E-10 |
| GO:0042593 | BP | | glucose homeostasis | 11/51 | 2.65E-09 |
| GO:0033500 | BP | | carbohydrate homeostasis | 11/51 | 2.69E-09 |
| GO:0006735 | BP | | NADH regeneration | 5/51 | 3.09E-09 |
| GO:0061621 | BP | | canonical glycolysis | 5/51 | 3.09E-09 |
| GO:0061718 | BP | | glucose catabolic process to pyruvate | 5/51 | 3.09E-09 |
| GO:0005355 | MF | | glucose transmembrane transporter activity | 6/51 | 3.41E-09 |
| GO:0015149 | MF | | hexose transmembrane transporter activity | 6/51 | 3.41E-09 |
| GO:0015145 | MF | | monosaccharide transmembrane transporter activity | 6/51 | 3.41E-09 |
| GO:0055056 | MF | | D-glucose transmembrane transporter activity | 5/51 | 3.41E-09 |
| GO:0051119 | MF | | sugar transmembrane transporter activity | 6/51 | 4.23E-09 |
| GO:0019674 | BP | | NAD metabolic process | 6/51 | 7.33E-09 |
| GO:0061620 | BP | | glycolytic process through glucose-6-phosphate | 5/51 | 8.18E-09 |
| GO:0006734 | BP | | NADH metabolic process | 6/51 | 8.73E-09 |
| GO:0061615 | BP | | glycolytic process through fructose-6-phosphate | 5/51 | 1.21E-08 |
| GO:0019320 | BP | | hexose catabolic process | 6/51 | 2.26E-08 |
| GO:0046365 | BP | | monosaccharide catabolic process | 6/51 | 4.41E-08 |
| GO:0015144 | MF | | carbohydrate transmembrane transporter activity | 6/51 | 5.20E-08 |
| GO:0006109 | BP | | regulation of carbohydrate metabolic process | 9/51 | 6.64E-08 |
| GO:0045913 | BP | | positive regulation of carbohydrate metabolic process | 7/51 | 6.92E-08 |
| GO:0032868 | BP | | response to insulin | 10/51 | 7.45E-08 |
| GO:0019200 | MF | | carbohydrate kinase activity | 5/51 | 8.40E-08 |
| GO:0043467 | BP | | regulation of generation of precursor metabolites and energy | 8/51 | 1.20E-07 |
| GO:0006112 | BP | | energy reserve metabolic process | 7/51 | 1.40E-07 |
| GO:1900542 | BP | | regulation of purine nucleotide metabolic process | 7/51 | 1.40E-07 |
| GO:0006140 | BP | | regulation of nucleotide metabolic process | 7/51 | 1.62E-07 |
| GO:1903578 | BP | | regulation of ATP metabolic process | 7/51 | 1.72E-07 |
| GO:0044262 | BP | | cellular carbohydrate metabolic process | 10/51 | 1.79E-07 |
| GO:0062013 | BP | | positive regulation of small molecule metabolic process | 8/51 | 2.12E-07 |
| GO:1901652 | BP | | response to peptide | 12/51 | 2.21E-07 |
| GO:0046835 | BP | | carbohydrate phosphorylation | 5/51 | 2.40E-07 |
| GO:0043470 | BP | | regulation of carbohydrate catabolic process | 6/51 | 2.97E-07 |
| GO:0043434 | BP | | response to peptide hormone | 11/51 | 3.29E-07 |
| GO:0046324 | BP | | regulation of glucose import | 6/51 | 4.91E-07 |
| GO:0062012 | BP | | regulation of small molecule metabolic process | 10/51 | 8.42E-07 |
| GO:0045121 | CC | | membrane raft | 10/51 | 9.37E-07 |
| GO:0098857 | CC | | membrane microdomain | 10/51 | 9.37E-07 |
| GO:0048029 | MF | | monosaccharide binding | 6/51 | 1.17E-06 |
| GO:0061418 | BP | | regulation of transcription from RNA polymerase II promoter in response to hypoxia | 4/51 | 1.22E-06 |
| GO:1903580 | BP | | positive regulation of ATP metabolic process | 5/51 | 1.74E-06 |
| GO:0045725 | BP | | positive regulation of glycogen biosynthetic process | 4/51 | 2.25E-06 |
| GO:0010827 | BP | | regulation of glucose transmembrane transport | 6/51 | 2.25E-06 |
| GO:0032869 | BP | | cellular response to insulin stimulus | 8/51 | 2.52E-06 |
| GO:0060284 | BP | | regulation of cell development | 11/51 | 2.57E-06 |
| GO:0070875 | BP | | positive regulation of glycogen metabolic process | 4/51 | 2.82E-06 |
| GO:0015293 | MF | | symporter activity | 7/51 | 3.34E-06 |
| GO:0045981 | BP | | positive regulation of nucleotide metabolic process | 5/51 | 3.87E-06 |
| GO:1900544 | BP | | positive regulation of purine nucleotide metabolic process | 5/51 | 3.87E-06 |
| GO:0010232 | BP | | vascular transport | 6/51 | 3.93E-06 |
| GO:0150104 | BP | | transport across blood-brain barrier | 6/51 | 3.93E-06 |
| GO:0006110 | BP | | regulation of glycolytic process | 5/51 | 4.17E-06 |
| GO:1905039 | BP | | carboxylic acid transmembrane transport | 7/51 | 5.08E-06 |
| GO:0043618 | BP | | regulation of transcription from RNA polymerase II promoter in response to stress | 5/51 | 5.08E-06 |
| GO:1903825 | BP | | organic acid transmembrane transport | 7/51 | 5.23E-06 |
| GO:0015980 | BP | | energy derivation by oxidation of organic compounds | 9/51 | 5.23E-06 |
| GO:0045821 | BP | | positive regulation of glycolytic process | 4/51 | 6.51E-06 |
| GO:0015291 | MF | | secondary active transmembrane transporter activity | 8/51 | 6.53E-06 |
| GO:0062197 | BP | | cellular response to chemical stress | 9/51 | 6.78E-06 |
| GO:0051960 | BP | | regulation of nervous system development | 10/51 | 6.80E-06 |
| GO:0043620 | BP | | regulation of DNA-templated transcription in response to stress | 5/51 | 8.70E-06 |
| GO:0015711 | BP | | organic anion transport | 9/51 | 1.29E-05 |
| GO:0050767 | BP | | regulation of neurogenesis | 9/51 | 1.29E-05 |
| GO:1901653 | BP | | cellular response to peptide | 9/51 | 1.29E-05 |
| GO:0051156 | BP | | glucose 6-phosphate metabolic process | 4/51 | 1.30E-05 |
| GO:0003018 | BP | | vascular process in circulatory system | 8/51 | 1.31E-05 |
| GO:0001938 | BP | | positive regulation of endothelial cell proliferation | 6/51 | 1.39E-05 |
| GO:0051302 | BP | | regulation of cell division | 7/51 | 1.71E-05 |
| GO:0010906 | BP | | regulation of glucose metabolic process | 6/51 | 2.50E-05 |
| GO:0071375 | BP | | cellular response to peptide hormone stimulus | 8/51 | 2.90E-05 |
| GO:0005979 | BP | | regulation of glycogen biosynthetic process | 4/51 | 3.52E-05 |
| GO:0010962 | BP | | regulation of glucan biosynthetic process | 4/51 | 3.52E-05 |
| GO:0001558 | BP | | regulation of cell growth | 9/51 | 3.72E-05 |
| GO:0050679 | BP | | positive regulation of epithelial cell proliferation | 7/51 | 3.72E-05 |
| GO:0005977 | BP | | glycogen metabolic process | 5/51 | 3.74E-05 |
| GO:0006073 | BP | | cellular glucan metabolic process | 5/51 | 3.92E-05 |
| GO:0044042 | BP | | glucan metabolic process | 5/51 | 3.92E-05 |
| GO:0015849 | BP | | organic acid transport | 8/51 | 4.80E-05 |
| GO:0005536 | MF | | glucose binding | 3/51 | 6.62E-05 |
| GO:0046326 | BP | | positive regulation of glucose import | 4/51 | 6.73E-05 |
| GO:0070873 | BP | | regulation of glycogen metabolic process | 4/51 | 6.73E-05 |
| GO:0006002 | BP | | fructose 6-phosphate metabolic process | 3/51 | 6.86E-05 |
| GO:0010675 | BP | | regulation of cellular carbohydrate metabolic process | 6/51 | 6.95E-05 |
| GO:2000177 | BP | | regulation of neural precursor cell proliferation | 5/51 | 7.37E-05 |
| GO:0045834 | BP | | positive regulation of lipid metabolic process | 6/51 | 7.65E-05 |
| GO:0032885 | BP | | regulation of polysaccharide biosynthetic process | 4/51 | 7.96E-05 |
| GO:0051781 | BP | | positive regulation of cell division | 5/51 | 8.95E-05 |
| GO:0045861 | BP | | negative regulation of proteolysis | 8/51 | 8.95E-05 |
| GO:0010907 | BP | | positive regulation of glucose metabolic process | 4/51 | 0.00010471 |
| GO:0016049 | BP | | cell growth | 9/51 | 0.00011178 |
| GO:0010828 | BP | | positive regulation of glucose transmembrane transport | 4/51 | 0.00012423 |
| GO:0044264 | BP | | cellular polysaccharide metabolic process | 5/51 | 0.00013023 |
| GO:0010817 | BP | | regulation of hormone levels | 9/51 | 0.00013023 |
| GO:0008631 | BP | | intrinsic apoptotic signaling pathway in response to oxidative stress | 4/51 | 0.00013023 |
| GO:0032881 | BP | | regulation of polysaccharide metabolic process | 4/51 | 0.00013023 |
| GO:1902895 | BP | | positive regulation of miRNA transcription | 4/51 | 0.00013023 |
| GO:2001242 | BP | | regulation of intrinsic apoptotic signaling pathway | 6/51 | 0.00013023 |
| GO:0032352 | BP | | positive regulation of hormone metabolic process | 3/51 | 0.00013023 |
| GO:0030900 | BP | | forebrain development | 8/51 | 0.00013544 |
| GO:0044282 | BP | | small molecule catabolic process | 8/51 | 0.00013544 |
| GO:0005978 | BP | | glycogen biosynthetic process | 4/51 | 0.00013544 |
| GO:0009250 | BP | | glucan biosynthetic process | 4/51 | 0.00013544 |
| GO:2000378 | BP | | negative regulation of reactive oxygen species metabolic process | 4/51 | 0.00014655 |
| GO:0050678 | BP | | regulation of epithelial cell proliferation | 8/51 | 0.00014822 |
| GO:0090276 | BP | | regulation of peptide hormone secretion | 6/51 | 0.00014879 |
| GO:0001936 | BP | | regulation of endothelial cell proliferation | 6/51 | 0.00015245 |
| GO:0002791 | BP | | regulation of peptide secretion | 6/51 | 0.00016124 |
| GO:0030246 | MF | | carbohydrate binding | 7/51 | 0.00016814 |
| GO:0046942 | BP | | carboxylic acid transport | 7/51 | 0.00017183 |
| GO:0045766 | BP | | positive regulation of angiogenesis | 6/51 | 0.00017183 |
| GO:0090087 | BP | | regulation of peptide transport | 6/51 | 0.00017183 |
| GO:1904018 | BP | | positive regulation of vasculature development | 6/51 | 0.00017183 |
| GO:0006089 | BP | | lactate metabolic process | 3/51 | 0.00017858 |
| GO:0061138 | BP | | morphogenesis of a branching epithelium | 6/51 | 0.0001801 |
| GO:0005976 | BP | | polysaccharide metabolic process | 5/51 | 0.00018572 |
| GO:2000179 | BP | | positive regulation of neural precursor cell proliferation | 4/51 | 0.00018572 |
| GO:0005402 | MF | | carbohydrate:cation symporter activity | 3/51 | 0.0001884 |
| GO:0098739 | BP | | import across plasma membrane | 6/51 | 0.00019306 |
| GO:0008514 | MF | | organic anion transmembrane transporter activity | 6/51 | 0.00019378 |
| GO:0022804 | MF | | active transmembrane transporter activity | 8/51 | 0.00020894 |
| GO:0009914 | BP | | hormone transport | 7/51 | 0.00020974 |
| GO:0001935 | BP | | endothelial cell proliferation | 6/51 | 0.00022751 |
| GO:0009925 | CC | | basal plasma membrane | 7/51 | 0.00022864 |
| GO:0097193 | BP | | intrinsic apoptotic signaling pathway | 7/51 | 0.00023093 |
| GO:0060644 | BP | | mammary gland epithelial cell differentiation | 3/51 | 0.00024452 |
| GO:0001763 | BP | | morphogenesis of a branching structure | 6/51 | 0.00025702 |
| GO:0010676 | BP | | positive regulation of cellular carbohydrate metabolic process | 4/51 | 0.00025702 |
| GO:0045178 | CC | | basal part of cell | 7/51 | 0.00027028 |
| GO:0001658 | BP | | branching involved in ureteric bud morphogenesis | 4/51 | 0.00027395 |
| GO:0007409 | BP | | axonogenesis | 8/51 | 0.00030095 |
| GO:0010975 | BP | | regulation of neuron projection development | 8/51 | 0.00030185 |
| GO:0048732 | BP | | gland development | 8/51 | 0.00030185 |
| GO:1902893 | BP | | regulation of miRNA transcription | 4/51 | 0.00030589 |
| GO:1902176 | BP | | negative regulation of oxidative stress-induced intrinsic apoptotic signaling pathway | 3/51 | 0.00032059 |
| GO:0090287 | BP | | regulation of cellular response to growth factor stimulus | 7/51 | 0.00032059 |
| GO:0061614 | BP | | miRNA transcription | 4/51 | 0.00032059 |
| GO:0050673 | BP | | epithelial cell proliferation | 8/51 | 0.00035543 |
| GO:0046716 | BP | | muscle cell cellular homeostasis | 3/51 | 0.00036518 |
| GO:0060675 | BP | | ureteric bud morphogenesis | 4/51 | 0.00038168 |
| GO:0072171 | BP | | mesonephric tubule morphogenesis | 4/51 | 0.0004037 |
| GO:0030072 | BP | | peptide hormone secretion | 6/51 | 0.00040867 |
| GO:0046885 | BP | | regulation of hormone biosynthetic process | 3/51 | 0.00040867 |
| GO:0051000 | BP | | positive regulation of nitric-oxide synthase activity | 3/51 | 0.00040867 |
| GO:0005158 | MF | | insulin receptor binding | 3/51 | 0.00041606 |
| GO:0050769 | BP | | positive regulation of neurogenesis | 6/51 | 0.00042585 |
| GO:0002790 | BP | | peptide secretion | 6/51 | 0.00045628 |
| GO:0030879 | BP | | mammary gland development | 5/51 | 0.00045864 |
| GO:0033692 | BP | | cellular polysaccharide biosynthetic process | 4/51 | 0.00046317 |
| GO:0019216 | BP | | regulation of lipid metabolic process | 7/51 | 0.00048395 |
| GO:0031346 | BP | | positive regulation of cell projection organization | 7/51 | 0.00049934 |
| GO:0036003 | BP | | positive regulation of transcription from RNA polymerase II promoter in response to stress | 3/51 | 0.00050655 |
| GO:1901522 | BP | | positive regulation of transcription from RNA polymerase II promoter involved in cellular response to chemical stimulus | 3/51 | 0.00050655 |
| GO:0046883 | BP | | regulation of hormone secretion | 6/51 | 0.00050655 |
| GO:0045765 | BP | | regulation of angiogenesis | 7/51 | 0.00052495 |
| GO:0007411 | BP | | axon guidance | 6/51 | 0.00053798 |
| GO:0097485 | BP | | neuron projection guidance | 6/51 | 0.0005477 |
| GO:0061564 | BP | | axon development | 8/51 | 0.00055083 |
| GO:0001667 | BP | | ameboidal-type cell migration | 8/51 | 0.00055577 |
| GO:0061351 | BP | | neural precursor cell proliferation | 5/51 | 0.00056237 |
| GO:1901342 | BP | | regulation of vasculature development | 7/51 | 0.00056544 |
| GO:0072078 | BP | | nephron tubule morphogenesis | 4/51 | 0.00056782 |
| GO:0098657 | BP | | import into cell | 6/51 | 0.00056782 |
| GO:0014065 | BP | | phosphatidylinositol 3-kinase signaling | 5/51 | 0.00056828 |
| GO:0050796 | BP | | regulation of insulin secretion | 5/51 | 0.00061595 |
| GO:0000271 | BP | | polysaccharide biosynthetic process | 4/51 | 0.00061595 |
| GO:0072088 | BP | | nephron epithelium morphogenesis | 4/51 | 0.00061595 |
| GO:0010631 | BP | | epithelial cell migration | 7/51 | 0.00061595 |
| GO:0043536 | BP | | positive regulation of blood vessel endothelial cell migration | 4/51 | 0.00063871 |
| GO:0045926 | BP | | negative regulation of growth | 6/51 | 0.00063871 |
| GO:0090132 | BP | | epithelium migration | 7/51 | 0.00063871 |
| GO:0030902 | BP | | hindbrain development | 5/51 | 0.00066058 |
| GO:0061333 | BP | | renal tubule morphogenesis | 4/51 | 0.00066321 |
| GO:0090130 | BP | | tissue migration | 7/51 | 0.00067984 |
| GO:0006814 | BP | | sodium ion transport | 6/51 | 0.00067984 |
| GO:0015833 | BP | | peptide transport | 6/51 | 0.00067984 |
| GO:0072028 | BP | | nephron morphogenesis | 4/51 | 0.00068346 |
| GO:0048754 | BP | | branching morphogenesis of an epithelial tube | 5/51 | 0.00070306 |
| GO:1903672 | BP | | positive regulation of sprouting angiogenesis | 3/51 | 0.00070725 |
| GO:2001233 | BP | | regulation of apoptotic signaling pathway | 7/51 | 0.0007078 |
| GO:0050708 | BP | | regulation of protein secretion | 6/51 | 0.0007078 |
| GO:0001678 | BP | | cellular glucose homeostasis | 5/51 | 0.0007103 |
| GO:0034637 | BP | | cellular carbohydrate biosynthetic process | 4/51 | 0.00073267 |
| GO:1902175 | BP | | regulation of oxidative stress-induced intrinsic apoptotic signaling pathway | 3/51 | 0.00076736 |
| GO:0016241 | BP | | regulation of macroautophagy | 5/51 | 0.0007901 |
| GO:0016323 | CC | | basolateral plasma membrane | 6/51 | 0.0009466 |
| GO:0046889 | BP | | positive regulation of lipid biosynthetic process | 4/51 | 0.00096089 |
| GO:1903034 | BP | | regulation of response to wounding | 5/51 | 0.0009882 |
| GO:0051962 | BP | | positive regulation of nervous system development | 6/51 | 0.00099996 |
| GO:0032770 | BP | | positive regulation of monooxygenase activity | 3/51 | 0.00101003 |
| GO:0016324 | CC | | apical plasma membrane | 7/51 | 0.00113989 |
| GO:0046943 | MF | | carboxylic acid transmembrane transporter activity | 5/51 | 0.00114022 |
| GO:0005342 | MF | | organic acid transmembrane transporter activity | 5/51 | 0.00114022 |
| GO:1902692 | BP | | regulation of neuroblast proliferation | 3/51 | 0.00120792 |
| GO:0010634 | BP | | positive regulation of epithelial cell migration | 5/51 | 0.00120894 |
| GO:0072080 | BP | | nephron tubule development | 4/51 | 0.00120894 |
| GO:0090277 | BP | | positive regulation of peptide hormone secretion | 4/51 | 0.00120894 |
| GO:0046879 | BP | | hormone secretion | 6/51 | 0.00120894 |
| GO:0019217 | BP | | regulation of fatty acid metabolic process | 4/51 | 0.00125103 |
| GO:0034599 | BP | | cellular response to oxidative stress | 6/51 | 0.00126865 |
| GO:0048015 | BP | | phosphatidylinositol-mediated signaling | 5/51 | 0.00126865 |
| GO:0016242 | BP | | negative regulation of macroautophagy | 3/51 | 0.00126865 |
| GO:0002793 | BP | | positive regulation of peptide secretion | 4/51 | 0.00127402 |
| GO:0060993 | BP | | kidney morphogenesis | 4/51 | 0.00127402 |
| GO:0042886 | BP | | amide transport | 6/51 | 0.00129753 |
| GO:0008361 | BP | | regulation of cell size | 5/51 | 0.00129753 |
| GO:0030073 | BP | | insulin secretion | 5/51 | 0.00129753 |
| GO:0001657 | BP | | ureteric bud development | 4/51 | 0.00129753 |
| GO:0061326 | BP | | renal tubule development | 4/51 | 0.00129753 |
| GO:0032350 | BP | | regulation of hormone metabolic process | 3/51 | 0.00132142 |
| GO:1905332 | BP | | positive regulation of morphogenesis of an epithelium | 3/51 | 0.00132142 |
| GO:0048661 | BP | | positive regulation of smooth muscle cell proliferation | 4/51 | 0.00132142 |
| GO:0072163 | BP | | mesonephric epithelium development | 4/51 | 0.00132142 |
| GO:0072164 | BP | | mesonephric tubule development | 4/51 | 0.00132142 |
| GO:0048017 | BP | | inositol lipid-mediated signaling | 5/51 | 0.00132142 |
| GO:0010632 | BP | | regulation of epithelial cell migration | 6/51 | 0.00132142 |
| GO:0042063 | BP | | gliogenesis | 6/51 | 0.00134051 |

**Supplementary Table 6.** The KEGG enrichment analysis for 51 genes contained in PPI network (only terms of pvalue<0.05 were displayed).

| ID | Description | GeneRatio | p.adjust |
| --- | --- | --- | --- |
| hsa04066 | HIF-1 signaling pathway | 19/38 | 1.76E-24 |
| hsa05230 | Central carbon metabolism in cancer | 17/38 | 1.76E-24 |
| hsa00010 | Glycolysis / Gluconeogenesis | 10/38 | 1.41E-11 |
| hsa04930 | Type II diabetes mellitus | 9/38 | 1.41E-11 |
| hsa01200 | Carbon metabolism | 9/38 | 5.75E-08 |
| hsa04152 | AMPK signaling pathway | 8/38 | 1.62E-06 |
| hsa04910 | Insulin signaling pathway | 8/38 | 3.66E-06 |
| hsa05205 | Proteoglycans in cancer | 9/38 | 5.63E-06 |
| hsa00051 | Fructose and mannose metabolism | 5/38 | 6.24E-06 |
| hsa04922 | Glucagon signaling pathway | 7/38 | 7.46E-06 |
| hsa04931 | Insulin resistance | 7/38 | 7.46E-06 |
| hsa05211 | Renal cell carcinoma | 6/38 | 8.49E-06 |
| hsa01230 | Biosynthesis of amino acids | 6/38 | 1.27E-05 |
| hsa04919 | Thyroid hormone signaling pathway | 7/38 | 1.27E-05 |
| hsa04973 | Carbohydrate digestion and absorption | 5/38 | 2.31E-05 |
| hsa04211 | Longevity regulating pathway | 6/38 | 2.89E-05 |
| hsa04151 | PI3K-Akt signaling pathway | 10/38 | 3.03E-05 |
| hsa04213 | Longevity regulating pathway - multiple species | 5/38 | 7.71E-05 |
| hsa00052 | Galactose metabolism | 4/38 | 9.45E-05 |
| hsa04920 | Adipocytokine signaling pathway | 5/38 | 0.00011777 |
| hsa05167 | Kaposi sarcoma-associated herpesvirus infection | 7/38 | 0.00019307 |
| hsa01521 | EGFR tyrosine kinase inhibitor resistance | 5/38 | 0.00020758 |
| hsa05415 | Diabetic cardiomyopathy | 7/38 | 0.00023608 |
| hsa04140 | Autophagy - animal | 6/38 | 0.00027221 |
| hsa04010 | MAPK signaling pathway | 8/38 | 0.000306 |
| hsa05224 | Breast cancer | 6/38 | 0.00031759 |
| hsa04150 | mTOR signaling pathway | 6/38 | 0.00042631 |
| hsa05215 | Prostate cancer | 5/38 | 0.0004389 |
| hsa04950 | Maturity onset diabetes of the young | 3/38 | 0.00121378 |
| hsa04917 | Prolactin signaling pathway | 4/38 | 0.00153242 |
| hsa04068 | FoxO signaling pathway | 5/38 | 0.00160127 |
| hsa05218 | Melanoma | 4/38 | 0.00160127 |
| hsa05214 | Glioma | 4/38 | 0.00180161 |
| hsa05207 | Chemical carcinogenesis - receptor activation | 6/38 | 0.00180161 |
| hsa05212 | Pancreatic cancer | 4/38 | 0.00180161 |
| hsa05226 | Gastric cancer | 5/38 | 0.00253004 |
| hsa00500 | Starch and sucrose metabolism | 3/38 | 0.00253004 |
| hsa04911 | Insulin secretion | 4/38 | 0.00253863 |
| hsa05210 | Colorectal cancer | 4/38 | 0.00253863 |
| hsa01250 | Biosynthesis of nucleotide sugars | 3/38 | 0.00253863 |
| hsa04014 | Ras signaling pathway | 6/38 | 0.00265376 |
| hsa05219 | Bladder cancer | 3/38 | 0.00327475 |
| hsa05225 | Hepatocellular carcinoma | 5/38 | 0.00366509 |
| hsa01522 | Endocrine resistance | 4/38 | 0.00375038 |
| hsa00520 | Amino sugar and nucleotide sugar metabolism | 3/38 | 0.00514888 |
| hsa04722 | Neurotrophin signaling pathway | 4/38 | 0.00735471 |
| hsa04923 | Regulation of lipolysis in adipocytes | 3/38 | 0.00785478 |
| hsa05213 | Endometrial cancer | 3/38 | 0.00785478 |
| hsa04015 | Rap1 signaling pathway | 5/38 | 0.00862259 |
| hsa05221 | Acute myeloid leukemia | 3/38 | 0.0111561 |
| hsa05163 | Human cytomegalovirus infection | 5/38 | 0.0111561 |
| hsa04550 | Signaling pathways regulating pluripotency of stem cells | 4/38 | 0.01263721 |
| hsa05220 | Chronic myeloid leukemia | 3/38 | 0.015302 |
| hsa05131 | Shigellosis | 5/38 | 0.01566017 |
| hsa04218 | Cellular senescence | 4/38 | 0.01626616 |
| hsa04012 | ErbB signaling pathway | 3/38 | 0.01977176 |
| hsa05235 | PD-L1 expression and PD-1 checkpoint pathway in cancer | 3/38 | 0.02205164 |
| hsa00030 | Pentose phosphate pathway | 2/38 | 0.02285113 |
| hsa05222 | Small cell lung cancer | 3/38 | 0.02333436 |
| hsa05231 | Choline metabolism in cancer | 3/38 | 0.02726488 |
| hsa04914 | Progesterone-mediated oocyte maturation | 3/38 | 0.02989197 |
| hsa04960 | Aldosterone-regulated sodium reabsorption | 2/38 | 0.03155823 |
| hsa05216 | Thyroid cancer | 2/38 | 0.03155823 |
| hsa05206 | MicroRNAs in cancer | 5/38 | 0.03355833 |
| hsa05165 | Human papillomavirus infection | 5/38 | 0.04267912 |
| hsa04935 | Growth hormone synthesis, secretion and action | 3/38 | 0.04267912 |
| hsa05166 | Human T-cell leukemia virus 1 infection | 4/38 | 0.04454525 |
| hsa05208 | Chemical carcinogenesis - reactive oxygen species | 4/38 | 0.04454525 |
| hsa00620 | Pyruvate metabolism | 2/38 | 0.04542755 |
|  |  |  |  |

**Supplementary Table 6.** The results of spearman correlation analysis for SLC2A1 and cell death-related genes (only the terms of p<0.05 were displayed).

|  | | | **cancer** | | | **genes** | | | **cor** | | | **pvalue** | | |
| --- | --- | --- | --- | --- | --- | --- | --- | --- | --- | --- | --- | --- | --- | --- |
| Ferroptosis | | | ACC | | | FANCD2 | | | 0.34535054 | | | 0.00193116 | | |
| Ferroptosis | | | ACC | | | FDFT1 | | | 0.22234664 | | | 0.04908377 | | |
| Ferroptosis | | | ACC | | | RRM2 | | | 0.37297955 | | | 0.00077086 | | |
| Ferroptosis | | | BLCA | | | CDKN1A | | | 0.28153474 | | | 8.35E-09 | | |
| Ferroptosis | | | BLCA | | | TTC35 | | | 0.12496842 | | | 0.01156549 | | |
| Ferroptosis | | | BLCA | | | NFE2L2 | | | 0.23777003 | | | 1.28E-06 | | |
| Ferroptosis | | | BLCA | | | FANCD2 | | | -0.1855867 | | | 0.00016739 | | |
| Ferroptosis | | | BLCA | | | RRM2 | | | 0.17772648 | | | 0.00031539 | | |
| Ferroptosis | | | BRCA | | | CDKN1A | | | 0.08095976 | | | 0.00711569 | | |
| Ferroptosis | | | BRCA | | | TTC35 | | | 0.15666717 | | | 1.68E-07 | | |
| Ferroptosis | | | BRCA | | | FANCD2 | | | 0.20608184 | | | 4.71E-12 | | |
| Ferroptosis | | | BRCA | | | CISD1 | | | 0.12436496 | | | 3.42E-05 | | |
| Ferroptosis | | | BRCA | | | CARS | | | 0.16863106 | | | 1.73E-08 | | |
| Ferroptosis | | | BRCA | | | RRM2 | | | 0.25497684 | | | 7.63E-18 | | |
| Ferroptosis | | | BRCA | | | GLS2 | | | -0.1478349 | | | 8.07E-07 | | |
| Ferroptosis | | | CESC | | | CDKN1A | | | 0.45801518 | | | 2.51E-17 | | |
| Ferroptosis | | | CESC | | | TTC35 | | | 0.24211259 | | | 1.80E-05 | | |
| Ferroptosis | | | CESC | | | NFE2L2 | | | 0.37252334 | | | 1.53E-11 | | |
| Ferroptosis | | | CESC | | | CISD1 | | | -0.2448322 | | | 1.43E-05 | | |
| Ferroptosis | | | CESC | | | RRM2 | | | 0.18008419 | | | 0.0015328 | | |
| Ferroptosis | | | CESC | | | LPCAT3 | | | 0.11410316 | | | 0.04575766 | | |
| Ferroptosis | | | CHOL | | | CARS | | | 0.35675676 | | | 0.03332777 | | |
| Ferroptosis | | | COAD | | | CDKN1A | | | 0.22579463 | | | 1.25E-06 | | |
| Ferroptosis | | | COAD | | | CISD1 | | | -0.1754535 | | | 0.00017558 | | |
| Ferroptosis | | | ESCA | | | CDKN1A | | | 0.43029074 | | | 1.42E-09 | | |
| Ferroptosis | | | ESCA | | | TTC35 | | | 0.24901255 | | | 0.00065376 | | |
| Ferroptosis | | | ESCA | | | NFE2L2 | | | 0.49335128 | | | 0 | | |
| Ferroptosis | | | ESCA | | | CISD1 | | | -0.1633714 | | | 0.02638354 | | |
| Ferroptosis | | | ESCA | | | CARS | | | -0.1525378 | | | 0.03827519 | | |
| Ferroptosis | | | ESCA | | | RRM2 | | | 0.18854858 | | | 0.01025357 | | |
| Ferroptosis | | | GBM | | | CDKN1A | | | 0.27927278 | | | 0.00027135 | | |
| Ferroptosis | | | GBM | | | FANCD2 | | | -0.2555397 | | | 0.00088996 | | |
| Ferroptosis | | | GBM | | | CARS | | | 0.18793223 | | | 0.01512197 | | |
| Ferroptosis | | | HNSC | | | CDKN1A | | | 0.37880963 | | | 0 | | |
| Ferroptosis | | | HNSC | | | TTC35 | | | 0.10346275 | | | 0.01805305 | | |
| Ferroptosis | | | HNSC | | | NFE2L2 | | | 0.13703266 | | | 0.0017147 | | |
| Ferroptosis | | | HNSC | | | FANCD2 | | | -0.1858193 | | | 2.00E-05 | | |
| Ferroptosis | | | HNSC | | | CISD1 | | | -0.2359867 | | | 5.35E-08 | | |
| Ferroptosis | | | HNSC | | | RRM2 | | | -0.0894712 | | | 0.04104322 | | |
| Ferroptosis | | | HNSC | | | GLS2 | | | -0.2251874 | | | 2.16E-07 | | |
| Ferroptosis | | | KICH | | | FANCD2 | | | 0.43655151 | | | 0.0002841 | | |
| Ferroptosis | | | KICH | | | CARS | | | 0.43371256 | | | 0.0003137 | | |
| Ferroptosis | | | KICH | | | RRM2 | | | 0.53103016 | | | 6.35E-06 | | |
| Ferroptosis | | | KIRC | | | CDKN1A | | | 0.18969667 | | | 1.06E-05 | | |
| Ferroptosis | | | KIRC | | | CISD1 | | | -0.1764448 | | | 4.25E-05 | | |
| Ferroptosis | | | KIRC | | | FDFT1 | | | -0.2199095 | | | 3.06E-07 | | |
| Ferroptosis | | | KIRC | | | CARS | | | 0.1875972 | | | 1.33E-05 | | |
| Ferroptosis | | | KIRC | | | RRM2 | | | 0.23334027 | | | 5.37E-08 | | |
| Ferroptosis | | | KIRC | | | GLS2 | | | -0.1035602 | | | 0.01670197 | | |
| Ferroptosis | | | KIRP | | | CDKN1A | | | 0.12860009 | | | 0.02833741 | | |
| Ferroptosis | | | KIRP | | | NFE2L2 | | | 0.15015072 | | | 0.01037762 | | |
| Ferroptosis | | | KIRP | | | FANCD2 | | | 0.31615055 | | | 4.22E-08 | | |
| Ferroptosis | | | KIRP | | | CISD1 | | | -0.212618 | | | 0.00026695 | | |
| Ferroptosis | | | KIRP | | | FDFT1 | | | 0.25360289 | | | 1.27E-05 | | |
| Ferroptosis | | | KIRP | | | CARS | | | 0.22539092 | | | 0.00010941 | | |
| Ferroptosis | | | KIRP | | | RRM2 | | | 0.39777793 | | | 2.45E-12 | | |
| Ferroptosis | | | KIRP | | | GLS2 | | | -0.1342378 | | | 0.02205855 | | |
| Ferroptosis | | | LGG | | | CDKN1A | | | 0.13769666 | | | 0.00148459 | | |
| Ferroptosis | | | LGG | | | TTC35 | | | 0.23800342 | | | 2.92E-08 | | |
| Ferroptosis | | | LGG | | | NFE2L2 | | | 0.1361508 | | | 0.00167989 | | |
| Ferroptosis | | | LGG | | | FANCD2 | | | -0.154446 | | | 0.00035882 | | |
| Ferroptosis | | | LGG | | | FDFT1 | | | 0.16435191 | | | 0.00014434 | | |
| Ferroptosis | | | LGG | | | RRM2 | | | -0.1489994 | | | 0.00057881 | | |
| Ferroptosis | | | LGG | | | GLS2 | | | 0.10125579 | | | 0.01972308 | | |
| Ferroptosis | | | LGG | | | LPCAT3 | | | -0.1735483 | | | 5.91E-05 | | |
| Ferroptosis | | | LIHC | | | FANCD2 | | | 0.20825462 | | | 5.25E-05 | | |
| Ferroptosis | | | LIHC | | | CISD1 | | | 0.12907308 | | | 0.01259952 | | |
| Ferroptosis | | | LIHC | | | RRM2 | | | 0.23174175 | | | 6.12E-06 | | |
| Ferroptosis | | | LIHC | | | GLS2 | | | -0.2037144 | | | 7.41E-05 | | |
| Ferroptosis | | | LIHC | | | LPCAT3 | | | -0.1592053 | | | 0.00206388 | | |
| Ferroptosis | | | LUAD | | | CDKN1A | | | 0.20506753 | | | 2.58E-06 | | |
| Ferroptosis | | | LUAD | | | TTC35 | | | -0.1355046 | | | 0.0020161 | | |
| Ferroptosis | | | LUAD | | | FANCD2 | | | 0.41866249 | | | 2.34E-23 | | |
| Ferroptosis | | | LUAD | | | CISD1 | | | 0.1547183 | | | 0.00041447 | | |
| Ferroptosis | | | LUAD | | | FDFT1 | | | -0.1490402 | | | 0.00067479 | | |
| Ferroptosis | | | LUAD | | | CARS | | | 0.23257296 | | | 8.85E-08 | | |
| Ferroptosis | | | LUAD | | | RRM2 | | | 0.61396203 | | | 7.02E-55 | | |
| Ferroptosis | | | LUAD | | | GLS2 | | | -0.4677035 | | | 1.85E-29 | | |
| Ferroptosis | | | LUAD | | | LPCAT3 | | | -0.1976908 | | | 5.93E-06 | | |
| Ferroptosis | | | LUSC | | | CDKN1A | | | 0.29914784 | | | 7.75E-12 | | |
| Ferroptosis | | | LUSC | | | TTC35 | | | 0.14311464 | | | 0.00130396 | | |
| Ferroptosis | | | LUSC | | | NFE2L2 | | | 0.28948404 | | | 3.79E-11 | | |
| Ferroptosis | | | LUSC | | | FDFT1 | | | 0.21143407 | | | 1.76E-06 | | |
| Ferroptosis | | | LUSC | | | LPCAT3 | | | 0.2530063 | | | 9.01E-09 | | |
| Ferroptosis | | | MESO | | | NFE2L2 | | | -0.3283152 | | | 0.00200249 | | |
| Ferroptosis | | | MESO | | | FANCD2 | | | 0.22681709 | | | 0.03484101 | | |
| Ferroptosis | | | MESO | | | CARS | | | 0.39658818 | | | 0.00016151 | | |
| Ferroptosis | | | MESO | | | RRM2 | | | 0.39088358 | | | 0.00020345 | | |
| Ferroptosis | | | OV | | | CISD1 | | | -0.12238 | | | 0.03184019 | | |
| Ferroptosis | | | OV | | | FDFT1 | | | -0.1413963 | | | 0.01305248 | | |
| Ferroptosis | | | PAAD | | | CDKN1A | | | 0.31976859 | | | 1.42E-05 | | |
| Ferroptosis | | | PAAD | | | TTC35 | | | 0.27373883 | | | 0.00022042 | | |
| Ferroptosis | | | PAAD | | | FANCD2 | | | 0.4391731 | | | 1.14E-09 | | |
| Ferroptosis | | | PAAD | | | CISD1 | | | -0.2403176 | | | 0.00122895 | | |
| Ferroptosis | | | PAAD | | | FDFT1 | | | 0.24767225 | | | 0.00085795 | | |
| Ferroptosis | | | PAAD | | | CARS | | | 0.40346285 | | | 2.87E-08 | | |
| Ferroptosis | | | PAAD | | | RRM2 | | | 0.51543741 | | | 0 | | |
| Ferroptosis | | | PAAD | | | GLS2 | | | -0.4613542 | | | 1.14E-10 | | |
| Ferroptosis | | | PCPG | | | CDKN1A | | | 0.18397835 | | | 0.01251638 | | |
| Ferroptosis | | | PCPG | | | TTC35 | | | -0.3252798 | | | 7.46E-06 | | |
| Ferroptosis | | | PCPG | | | NFE2L2 | | | 0.22236135 | | | 0.00246558 | | |
| Ferroptosis | | | PCPG | | | FDFT1 | | | -0.1735008 | | | 0.01860753 | | |
| Ferroptosis | | | PCPG | | | CARS | | | -0.1772803 | | | 0.01616415 | | |
| Ferroptosis | | | PCPG | | | GLS2 | | | -0.4467666 | | | 3.03E-10 | | |
| Ferroptosis | | | PRAD | | | FANCD2 | | | 0.21960403 | | | 7.46E-07 | | |
| Ferroptosis | | | PRAD | | | CISD1 | | | -0.1440289 | | | 0.0012691 | | |
| Ferroptosis | | | PRAD | | | FDFT1 | | | 0.21752375 | | | 9.54E-07 | | |
| Ferroptosis | | | PRAD | | | RRM2 | | | 0.10223696 | | | 0.02250354 | | |
| Ferroptosis | | | PRAD | | | GLS2 | | | -0.0921739 | | | 0.03976755 | | |
| Ferroptosis | | | PRAD | | | LPCAT3 | | | 0.16446935 | | | 0.00022763 | | |
| Ferroptosis | | | READ | | | CDKN1A | | | 0.23274385 | | | 0.00303174 | | |
| Ferroptosis | | | SARC | | | NFE2L2 | | | -0.1441368 | | | 0.01935635 | | |
| Ferroptosis | | | SARC | | | FANCD2 | | | 0.30502094 | | | 5.21E-07 | | |
| Ferroptosis | | | SARC | | | FDFT1 | | | 0.20562862 | | | 0.00081277 | | |
| Ferroptosis | | | SARC | | | RRM2 | | | 0.39262051 | | | 5.65E-11 | | |
| Ferroptosis | | | SARC | | | LPCAT3 | | | -0.1722063 | | | 0.00515438 | | |
| Ferroptosis | | | SKCM | | | CDKN1A | | | 0.14208642 | | | 0.00196731 | | |
| Ferroptosis | | | SKCM | | | TTC35 | | | -0.2245082 | | | 8.10E-07 | | |
| Ferroptosis | | | SKCM | | | RRM2 | | | 0.16921155 | | | 0.00022245 | | |
| Ferroptosis | | | SKCM | | | LPCAT3 | | | 0.1369313 | | | 0.00286374 | | |
| Ferroptosis | | | STAD | | | CDKN1A | | | 0.34372678 | | | 5.95E-13 | | |
| Ferroptosis | | | STAD | | | FANCD2 | | | 0.21596353 | | | 9.05E-06 | | |
| Ferroptosis | | | STAD | | | CISD1 | | | -0.1151759 | | | 0.01892268 | | |
| Ferroptosis | | | STAD | | | FDFT1 | | | 0.20806966 | | | 1.93E-05 | | |
| Ferroptosis | | | STAD | | | CARS | | | 0.21815592 | | | 7.29E-06 | | |
| Ferroptosis | | | STAD | | | RRM2 | | | 0.21982261 | | | 6.18E-06 | | |
| Ferroptosis | | | TGCT | | | CDKN1A | | | 0.52427494 | | | 0 | | |
| Ferroptosis | | | TGCT | | | FANCD2 | | | -0.1890029 | | | 0.01824817 | | |
| Ferroptosis | | | TGCT | | | CISD1 | | | 0.30135449 | | | 0.00014135 | | |
| Ferroptosis | | | TGCT | | | FDFT1 | | | -0.2401732 | | | 0.00258924 | | |
| Ferroptosis | | | TGCT | | | CARS | | | 0.15759827 | | | 0.04951299 | | |
| Ferroptosis | | | TGCT | | | RRM2 | | | 0.68967774 | | | 0 | | |
| Ferroptosis | | | TGCT | | | GLS2 | | | 0.50695263 | | | 5.12E-12 | | |
| Ferroptosis | | | TGCT | | | LPCAT3 | | | 0.48399741 | | | 2.18E-10 | | |
| Ferroptosis | | | THCA | | | CDKN1A | | | 0.29309346 | | | 1.59E-11 | | |
| Ferroptosis | | | THCA | | | TTC35 | | | -0.1464163 | | | 0.00089067 | | |
| Ferroptosis | | | THCA | | | FANCD2 | | | 0.32295035 | | | 8.68E-14 | | |
| Ferroptosis | | | THCA | | | CISD1 | | | -0.097983 | | | 0.02647347 | | |
| Ferroptosis | | | THCA | | | FDFT1 | | | -0.1476878 | | | 0.00079275 | | |
| Ferroptosis | | | THCA | | | CARS | | | -0.2387116 | | | 4.43E-08 | | |
| Ferroptosis | | | THCA | | | RRM2 | | | 0.20726369 | | | 2.31E-06 | | |
| Ferroptosis | | | THCA | | | GLS2 | | | 0.32921216 | | | 2.66E-14 | | |
| Ferroptosis | | | THYM | | | CDKN1A | | | 0.40156261 | | | 6.60E-06 | | |
| Ferroptosis | | | THYM | | | NFE2L2 | | | 0.19747205 | | | 0.0307782 | | |
| Ferroptosis | | | THYM | | | CISD1 | | | 0.26380999 | | | 0.00369389 | | |
| Ferroptosis | | | THYM | | | CARS | | | 0.24795472 | | | 0.00644502 | | |
| Ferroptosis | | | THYM | | | GLS2 | | | 0.21191749 | | | 0.02030675 | | |
| Ferroptosis | | | THYM | | | LPCAT3 | | | 0.34155844 | | | 0.00014719 | | |
| Ferroptosis | | | UCEC | | | NFE2L2 | | | 0.12729206 | | | 0.00326142 | | |
| Ferroptosis | | | UCEC | | | FANCD2 | | | 0.21209302 | | | 8.24E-07 | | |
| Ferroptosis | | | UCEC | | | CARS | | | 0.1513378 | | | 0.00046137 | | |
| Ferroptosis | | | UCEC | | | RRM2 | | | 0.21416834 | | | 6.40E-07 | | |
| Ferroptosis | | | UCEC | | | LPCAT3 | | | -0.136158 | | | 0.00164229 | | |
| Ferroptosis | | | UCS | | | CDKN1A | | | -0.3098911 | | | 0.01934765 | | |
| Ferroptosis | | | UCS | | | GLS2 | | | 0.42500648 | | | 0.0010867 | | |
| Ferroptosis | | | UCS | | | LPCAT3 | | | 0.38682914 | | | 0.0031472 | | |
| Ferroptosis | | | UVM | | | CDKN1A | | | 0.47998125 | | | 8.65E-06 | | |
| Ferroptosis | | | UVM | | | NFE2L2 | | | -0.4384904 | | | 5.64E-05 | | |
| Ferroptosis | | | UVM | | | FANCD2 | | | -0.2626582 | | | 0.01883015 | | |
| Ferroptosis | | | UVM | | | LPCAT3 | | | 0.30067979 | | | 0.00691994 | | |
| anoikis | | | BLCA | | | CEACAM6 | | | 0.19395831 | | | 8.04E-05 | | |
| anoikis | | | BLCA | | | CEACAM5 | | | 0.19499531 | | | 7.35E-05 | | |
| anoikis | | | BRCA | | | BRMS1 | | | 0.09331509 | | | 0.00191055 | | |
| anoikis | | | BRCA | | | PTK2 | | | 0.18353056 | | | 8.08E-10 | | |
| anoikis | | | BRCA | | | NTRK2 | | | -0.1343427 | | | 7.50E-06 | | |
| anoikis | | | BRCA | | | BCL2L11 | | | -0.0745542 | | | 0.01321883 | | |
| anoikis | | | BRCA | | | SRC | | | 0.25387001 | | | 1.07E-17 | | |
| anoikis | | | BRCA | | | CEACAM6 | | | -0.0942653 | | | 0.00171537 | | |
| anoikis | | | BRCA | | | CAV1 | | | -0.1382655 | | | 4.01E-06 | | |
| anoikis | | | BRCA | | | AKT1 | | | 0.07098607 | | | 0.01832808 | | |
| anoikis | | | BRCA | | | CEACAM5 | | | -0.0970245 | | | 0.00124768 | | |
| anoikis | | | CESC | | | NTRK2 | | | 0.18030774 | | | 0.00151185 | | |
| anoikis | | | CESC | | | BCL2L11 | | | 0.19144939 | | | 0.00074618 | | |
| anoikis | | | CESC | | | SRC | | | 0.21644775 | | | 0.00013215 | | |
| anoikis | | | CESC | | | CAV1 | | | 0.33128147 | | | 2.69E-09 | | |
| anoikis | | | CHOL | | | CEACAM6 | | | 0.46512227 | | | 0.00464879 | | |
| anoikis | | | CHOL | | | CEACAM5 | | | 0.45045045 | | | 0.00628556 | | |
| anoikis | | | COAD | | | BRMS1 | | | 0.13019726 | | | 0.00549327 | | |
| anoikis | | | COAD | | | CEACAM6 | | | 0.15189803 | | | 0.00118188 | | |
| anoikis | | | COAD | | | ITGB1 | | | 0.11188619 | | | 0.01712319 | | |
| anoikis | | | ESCA | | | BRMS1 | | | 0.17280429 | | | 0.01876435 | | |
| anoikis | | | ESCA | | | PTK2 | | | 0.26247299 | | | 0.00032128 | | |
| anoikis | | | ESCA | | | NTRK2 | | | 0.47103408 | | | 1.45E-11 | | |
| anoikis | | | ESCA | | | SRC | | | -0.4262916 | | | 2.09E-09 | | |
| anoikis | | | ESCA | | | CEACAM6 | | | -0.2092244 | | | 0.004327 | | |
| anoikis | | | ESCA | | | CAV1 | | | 0.47927486 | | | 3.31E-12 | | |
| anoikis | | | ESCA | | | AKT1 | | | 0.23837989 | | | 0.00111671 | | |
| anoikis | | | ESCA | | | CEACAM5 | | | -0.3354744 | | | 3.48E-06 | | |
| anoikis | | | GBM | | | PTK2 | | | -0.3058942 | | | 6.30E-05 | | |
| anoikis | | | GBM | | | SRC | | | -0.2208741 | | | 0.00419802 | | |
| anoikis | | | GBM | | | CAV1 | | | 0.46101343 | | | 5.41E-10 | | |
| anoikis | | | GBM | | | AKT1 | | | 0.19699929 | | | 0.01082178 | | |
| anoikis | | | GBM | | | ITGB1 | | | 0.33495058 | | | 1.09E-05 | | |
| anoikis | | | HNSC | | | PTK2 | | | 0.20202771 | | | 3.27E-06 | | |
| anoikis | | | HNSC | | | BCL2L11 | | | -0.1862934 | | | 1.90E-05 | | |
| anoikis | | | HNSC | | | SRC | | | 0.08786894 | | | 0.04478803 | | |
| anoikis | | | HNSC | | | CAV1 | | | 0.31209308 | | | 3.91E-13 | | |
| anoikis | | | HNSC | | | AKT1 | | | 0.20089234 | | | 3.73E-06 | | |
| anoikis | | | HNSC | | | ITGB1 | | | 0.22509611 | | | 2.18E-07 | | |
| anoikis | | | KICH | | | AKT1 | | | -0.3258741 | | | 0.00783029 | | |
| anoikis | | | KIRC | | | BRMS1 | | | -0.0921276 | | | 0.03332947 | | |
| anoikis | | | KIRC | | | BCL2L11 | | | 0.18304081 | | | 2.08E-05 | | |
| anoikis | | | KIRC | | | CAV1 | | | 0.34073497 | | | 6.93E-16 | | |
| anoikis | | | KIRC | | | AKT1 | | | 0.10836362 | | | 0.0122234 | | |
| anoikis | | | KIRC | | | ITGB1 | | | 0.34068698 | | | 7.01E-16 | | |
| anoikis | | | KIRP | | | PTK2 | | | 0.21815641 | | | 0.00018245 | | |
| anoikis | | | KIRP | | | NTRK2 | | | 0.16412839 | | | 0.0050478 | | |
| anoikis | | | KIRP | | | SRC | | | 0.11970256 | | | 0.04134868 | | |
| anoikis | | | KIRP | | | CEACAM6 | | | 0.1894344 | | | 0.00116608 | | |
| anoikis | | | KIRP | | | ITGB1 | | | 0.28776717 | | | 6.64E-07 | | |
| anoikis | | | KIRP | | | CEACAM5 | | | 0.21027927 | | | 0.0003037 | | |
| anoikis | | | LGG | | | NTRK2 | | | 0.10698571 | | | 0.01372976 | | |
| anoikis | | | LGG | | | SRC | | | -0.1588679 | | | 0.00024054 | | |
| anoikis | | | LGG | | | CAV1 | | | 0.27931674 | | | 5.91E-11 | | |
| anoikis | | | LGG | | | ITGB1 | | | 0.20641941 | | | 1.65E-06 | | |
| anoikis | | | LIHC | | | BRMS1 | | | 0.10427946 | | | 0.04418307 | | |
| anoikis | | | LIHC | | | SRC | | | 0.28016571 | | | 4.26E-08 | | |
| anoikis | | | LIHC | | | CEACAM6 | | | 0.19058795 | | | 0.00021348 | | |
| anoikis | | | LIHC | | | CAV1 | | | 0.11064889 | | | 0.03269363 | | |
| anoikis | | | LIHC | | | AKT1 | | | -0.149599 | | | 0.00381194 | | |
| anoikis | | | LIHC | | | CEACAM5 | | | 0.2767669 | | | 5.52E-08 | | |
| anoikis | | | LUAD | | | PTK2 | | | -0.1064412 | | | 0.01546749 | | |
| anoikis | | | LUAD | | | NTRK2 | | | -0.1725326 | | | 8.04E-05 | | |
| anoikis | | | LUAD | | | BCL2L11 | | | 0.18151912 | | | 3.30E-05 | | |
| anoikis | | | LUAD | | | CEACAM6 | | | -0.165514 | | | 0.00015658 | | |
| anoikis | | | LUAD | | | ITGB1 | | | 0.47717882 | | | 9.32E-31 | | |
| anoikis | | | LUSC | | | BRMS1 | | | 0.09963926 | | | 0.02558552 | | |
| anoikis | | | LUSC | | | PTK2 | | | 0.21225673 | | | 1.60E-06 | | |
| anoikis | | | LUSC | | | NTRK2 | | | 0.33072383 | | | 2.81E-14 | | |
| anoikis | | | LUSC | | | BCL2L11 | | | -0.1286427 | | | 0.00388806 | | |
| anoikis | | | LUSC | | | SRC | | | 0.10075236 | | | 0.02397609 | | |
| anoikis | | | LUSC | | | CAV1 | | | 0.09321223 | | | 0.0368164 | | |
| anoikis | | | LUSC | | | AKT1 | | | 0.30624902 | | | 2.32E-12 | | |
| anoikis | | | MESO | | | NTRK2 | | | -0.3387585 | | | 0.00140876 | | |
| anoikis | | | MESO | | | AKT1 | | | 0.25736313 | | | 0.01632913 | | |
| anoikis | | | MESO | | | ITGB1 | | | 0.24396734 | | | 0.02300136 | | |
| anoikis | | | OV | | | SRC | | | 0.14912702 | | | 0.00881382 | | |
| anoikis | | | OV | | | CEACAM6 | | | -0.1688038 | | | 0.00296056 | | |
| anoikis | | | PAAD | | | PTK2 | | | 0.21299981 | | | 0.00426842 | | |
| anoikis | | | PAAD | | | NTRK2 | | | -0.3397736 | | | 3.75E-06 | | |
| anoikis | | | PAAD | | | SRC | | | 0.45172933 | | | 3.24E-10 | | |
| anoikis | | | PAAD | | | CEACAM6 | | | 0.35277883 | | | 1.28E-06 | | |
| anoikis | | | PAAD | | | AKT1 | | | 0.34479526 | | | 2.65E-06 | | |
| anoikis | | | PAAD | | | ITGB1 | | | 0.31899651 | | | 1.50E-05 | | |
| anoikis | | | PAAD | | | CEACAM5 | | | 0.44696179 | | | 3.57E-10 | | |
| anoikis | | | PCPG | | | BRMS1 | | | -0.181318 | | | 0.01386804 | | |
| anoikis | | | PCPG | | | PTK2 | | | 0.29906378 | | | 4.04E-05 | | |
| anoikis | | | PCPG | | | NTRK2 | | | 0.36439868 | | | 4.46E-07 | | |
| anoikis | | | PCPG | | | CAV1 | | | 0.4002408 | | | 2.42E-08 | | |
| anoikis | | | PCPG | | | ITGB1 | | | 0.28113695 | | | 0.00011776 | | |
| anoikis | | | PRAD | | | BCL2L11 | | | 0.16187246 | | | 0.00028651 | | |
| anoikis | | | PRAD | | | CAV1 | | | -0.0964761 | | | 0.03135285 | | |
| anoikis | | | PRAD | | | AKT1 | | | 0.21184334 | | | 1.84E-06 | | |
| anoikis | | | PRAD | | | ITGB1 | | | -0.1542404 | | | 0.00055222 | | |
| anoikis | | | READ | | | AKT1 | | | 0.1548683 | | | 0.04988907 | | |
| anoikis | | | SARC | | | NTRK2 | | | -0.2068219 | | | 0.00075669 | | |
| anoikis | | | SARC | | | SRC | | | 0.2000004 | | | 0.00113275 | | |
| anoikis | | | SARC | | | CAV1 | | | 0.18517915 | | | 0.00260666 | | |
| anoikis | | | SARC | | | AKT1 | | | 0.22757683 | | | 0.00020497 | | |
| anoikis | | | SARC | | | ITGB1 | | | 0.23894014 | | | 9.53E-05 | | |
| anoikis | | | SKCM | | | BRMS1 | | | 0.12946323 | | | 0.00482816 | | |
| anoikis | | | SKCM | | | SRC | | | 0.11857388 | | | 0.00988323 | | |
| anoikis | | | SKCM | | | CEACAM6 | | | 0.24234619 | | | 9.48E-08 | | |
| anoikis | | | SKCM | | | CAV1 | | | -0.1422353 | | | 0.00194574 | | |
| anoikis | | | SKCM | | | AKT1 | | | 0.18042771 | | | 8.15E-05 | | |
| anoikis | | | SKCM | | | ITGB1 | | | -0.1487986 | | | 0.0011846 | | |
| anoikis | | | SKCM | | | CEACAM5 | | | 0.24530724 | | | 6.53E-08 | | |
| anoikis | | | STAD | | | BRMS1 | | | 0.10245422 | | | 0.03694704 | | |
| anoikis | | | STAD | | | PTK2 | | | 0.15580945 | | | 0.00145269 | | |
| anoikis | | | STAD | | | BCL2L11 | | | 0.10913 | | | 0.02621007 | | |
| anoikis | | | STAD | | | SRC | | | 0.16414026 | | | 0.00078936 | | |
| anoikis | | | STAD | | | CEACAM6 | | | 0.30072188 | | | 4.03E-10 | | |
| anoikis | | | STAD | | | CAV1 | | | -0.1619669 | | | 0.00092812 | | |
| anoikis | | | STAD | | | CEACAM5 | | | 0.20087151 | | | 8.59E-05 | | |
| anoikis | | | TGCT | | | BRMS1 | | | 0.28715841 | | | 0.00029431 | | |
| anoikis | | | TGCT | | | PTK2 | | | -0.4610137 | | | 2.10E-09 | | |
| anoikis | | | TGCT | | | NTRK2 | | | 0.23757172 | | | 0.00288872 | | |
| anoikis | | | TGCT | | | CEACAM6 | | | -0.287503 | | | 0.00028924 | | |
| anoikis | | | TGCT | | | CAV1 | | | 0.62199428 | | | 0 | | |
| anoikis | | | TGCT | | | CEACAM5 | | | 0.20394437 | | | 0.01065924 | | |
| anoikis | | | THCA | | | BRMS1 | | | -0.242669 | | | 2.60E-08 | | |
| anoikis | | | THCA | | | SRC | | | 0.32770076 | | | 2.63E-14 | | |
| anoikis | | | THCA | | | CEACAM6 | | | 0.47546173 | | | 2.70E-30 | | |
| anoikis | | | THCA | | | CAV1 | | | 0.45349627 | | | 0 | | |
| anoikis | | | THCA | | | ITGB1 | | | 0.33221222 | | | 1.49E-14 | | |
| anoikis | | | THCA | | | CEACAM5 | | | 0.27375099 | | | 2.86E-10 | | |
| anoikis | | | THYM | | | PTK2 | | | 0.30404889 | | | 0.00077457 | | |
| anoikis | | | THYM | | | NTRK2 | | | 0.30626432 | | | 0.00070617 | | |
| anoikis | | | THYM | | | BCL2L11 | | | 0.52406417 | | | 1.23E-09 | | |
| anoikis | | | THYM | | | SRC | | | 0.41583443 | | | 2.89E-06 | | |
| anoikis | | | THYM | | | CEACAM6 | | | 0.41409663 | | | 2.58E-06 | | |
| anoikis | | | THYM | | | AKT1 | | | 0.29037433 | | | 0.00134996 | | |
| anoikis | | | THYM | | | CEACAM5 | | | 0.30591711 | | | 0.000679 | | |
| anoikis | | | UCEC | | | BRMS1 | | | -0.1024913 | | | 0.01793899 | | |
| anoikis | | | UCEC | | | PTK2 | | | 0.19399137 | | | 6.72E-06 | | |
| anoikis | | | UCEC | | | BCL2L11 | | | 0.13564465 | | | 0.00171075 | | |
| anoikis | | | UCEC | | | ITGB1 | | | 0.09014283 | | | 0.03751243 | | |
| anoikis | | | UCS | | | CEACAM6 | | | 0.48202741 | | | 0.00014667 | | |
| anoikis | | | UCS | | | CEACAM5 | | | 0.31236784 | | | 0.01800058 | | |
| anoikis | | | UVM | | | SRC | | | 0.32390999 | | | 0.00352146 | | |
| anoikis | | | UVM | | | AKT1 | | | 0.33947492 | | | 0.00217655 | | |
| Necroptosis | | ACC | | | FANCD2 | | | 0.34535054 | | | 0.00193116 | | |  |
| Necroptosis | | ACC | | | FDFT1 | | | 0.22234664 | | | 0.04908377 | | |  |
| Necroptosis | | ACC | | | RRM2 | | | 0.37297955 | | | 0.00077086 | | |  |
| Necroptosis | | BLCA | | | CDKN1A | | | 0.28153474 | | | 8.35E-09 | | |  |
| Necroptosis | | BLCA | | | TTC35 | | | 0.12496842 | | | 0.01156549 | | |  |
| Necroptosis | | BLCA | | | NFE2L2 | | | 0.23777003 | | | 1.28E-06 | | |  |
| Necroptosis | | BLCA | | | FANCD2 | | | -0.1855867 | | | 0.00016739 | | |  |
| Necroptosis | | BLCA | | | RRM2 | | | 0.17772648 | | | 0.00031539 | | |  |
| Necroptosis | | BRCA | | | CDKN1A | | | 0.08095976 | | | 0.00711569 | | |  |
| Necroptosis | | BRCA | | | TTC35 | | | 0.15666717 | | | 1.68E-07 | | |  |
| Necroptosis | | BRCA | | | FANCD2 | | | 0.20608184 | | | 4.71E-12 | | |  |
| Necroptosis | | BRCA | | | CISD1 | | | 0.12436496 | | | 3.42E-05 | | |  |
| Necroptosis | | BRCA | | | CARS | | | 0.16863106 | | | 1.73E-08 | | |  |
| Necroptosis | | BRCA | | | RRM2 | | | 0.25497684 | | | 7.63E-18 | | |  |
| Necroptosis | | BRCA | | | GLS2 | | | -0.1478349 | | | 8.07E-07 | | |  |
| Necroptosis | | CESC | | | CDKN1A | | | 0.45801518 | | | 2.51E-17 | | |  |
| Necroptosis | | CESC | | | TTC35 | | | 0.24211259 | | | 1.80E-05 | | |  |
| Necroptosis | | CESC | | | NFE2L2 | | | 0.37252334 | | | 1.53E-11 | | |  |
| Necroptosis | | CESC | | | CISD1 | | | -0.2448322 | | | 1.43E-05 | | |  |
| Necroptosis | | CESC | | | RRM2 | | | 0.18008419 | | | 0.0015328 | | |  |
| Necroptosis | | CESC | | | LPCAT3 | | | 0.11410316 | | | 0.04575766 | | |  |
| Necroptosis | | CHOL | | | CARS | | | 0.35675676 | | | 0.03332777 | | |  |
| Necroptosis | | COAD | | | CDKN1A | | | 0.22579463 | | | 1.25E-06 | | |  |
| Necroptosis | | COAD | | | CISD1 | | | -0.1754535 | | | 0.00017558 | | |  |
| Necroptosis | | ESCA | | | CDKN1A | | | 0.43029074 | | | 1.42E-09 | | |  |
| Necroptosis | | ESCA | | | TTC35 | | | 0.24901255 | | | 0.00065376 | | |  |
| Necroptosis | | ESCA | | | NFE2L2 | | | 0.49335128 | | | 0 | | |  |
| Necroptosis | | ESCA | | | CISD1 | | | -0.1633714 | | | 0.02638354 | | |  |
| Necroptosis | | ESCA | | | CARS | | | -0.1525378 | | | 0.03827519 | | |  |
| Necroptosis | | ESCA | | | RRM2 | | | 0.18854858 | | | 0.01025357 | | |  |
| Necroptosis | | GBM | | | CDKN1A | | | 0.27927278 | | | 0.00027135 | | |  |
| Necroptosis | | GBM | | | FANCD2 | | | -0.2555397 | | | 0.00088996 | | |  |
| Necroptosis | | GBM | | | CARS | | | 0.18793223 | | | 0.01512197 | | |  |
| Necroptosis | | HNSC | | | CDKN1A | | | 0.37880963 | | | 0 | | |  |
| Necroptosis | | HNSC | | | TTC35 | | | 0.10346275 | | | 0.01805305 | | |  |
| Necroptosis | | HNSC | | | NFE2L2 | | | 0.13703266 | | | 0.0017147 | | |  |
| Necroptosis | | HNSC | | | FANCD2 | | | -0.1858193 | | | 2.00E-05 | | |  |
| Necroptosis | | HNSC | | | CISD1 | | | -0.2359867 | | | 5.35E-08 | | |  |
| Necroptosis | | HNSC | | | RRM2 | | | -0.0894712 | | | 0.04104322 | | |  |
| Necroptosis | | HNSC | | | GLS2 | | | -0.2251874 | | | 2.16E-07 | | |  |
| Necroptosis | | KICH | | | FANCD2 | | | 0.43655151 | | | 0.0002841 | | |  |
| Necroptosis | | KICH | | | CARS | | | 0.43371256 | | | 0.0003137 | | |  |
| Necroptosis | | KICH | | | RRM2 | | | 0.53103016 | | | 6.35E-06 | | |  |
| Necroptosis | | KIRC | | | CDKN1A | | | 0.18969667 | | | 1.06E-05 | | |  |
| Necroptosis | | KIRC | | | CISD1 | | | -0.1764448 | | | 4.25E-05 | | |  |
| Necroptosis | | KIRC | | | FDFT1 | | | -0.2199095 | | | 3.06E-07 | | |  |
| Necroptosis | | KIRC | | | CARS | | | 0.1875972 | | | 1.33E-05 | | |  |
| Necroptosis | | KIRC | | | RRM2 | | | 0.23334027 | | | 5.37E-08 | | |  |
| Necroptosis | | KIRC | | | GLS2 | | | -0.1035602 | | | 0.01670197 | | |  |
| Necroptosis | | KIRP | | | CDKN1A | | | 0.12860009 | | | 0.02833741 | | |  |
| Necroptosis | | KIRP | | | NFE2L2 | | | 0.15015072 | | | 0.01037762 | | |  |
| Necroptosis | | KIRP | | | FANCD2 | | | 0.31615055 | | | 4.22E-08 | | |  |
| Necroptosis | | KIRP | | | CISD1 | | | -0.212618 | | | 0.00026695 | | |  |
| Necroptosis | | KIRP | | | FDFT1 | | | 0.25360289 | | | 1.27E-05 | | |  |
| Necroptosis | | KIRP | | | CARS | | | 0.22539092 | | | 0.00010941 | | |  |
| Necroptosis | | KIRP | | | RRM2 | | | 0.39777793 | | | 2.45E-12 | | |  |
| Necroptosis | | KIRP | | | GLS2 | | | -0.1342378 | | | 0.02205855 | | |  |
| Necroptosis | | LGG | | | CDKN1A | | | 0.13769666 | | | 0.00148459 | | |  |
| Necroptosis | | LGG | | | TTC35 | | | 0.23800342 | | | 2.92E-08 | | |  |
| Necroptosis | | LGG | | | NFE2L2 | | | 0.1361508 | | | 0.00167989 | | |  |
| Necroptosis | | LGG | | | FANCD2 | | | -0.154446 | | | 0.00035882 | | |  |
| Necroptosis | | LGG | | | FDFT1 | | | 0.16435191 | | | 0.00014434 | | |  |
| Necroptosis | | LGG | | | RRM2 | | | -0.1489994 | | | 0.00057881 | | |  |
| Necroptosis | | LGG | | | GLS2 | | | 0.10125579 | | | 0.01972308 | | |  |
| Necroptosis | | LGG | | | LPCAT3 | | | -0.1735483 | | | 5.91E-05 | | |  |
| Necroptosis | | LIHC | | | FANCD2 | | | 0.20825462 | | | 5.25E-05 | | |  |
| Necroptosis | | LIHC | | | CISD1 | | | 0.12907308 | | | 0.01259952 | | |  |
| Necroptosis | | LIHC | | | RRM2 | | | 0.23174175 | | | 6.12E-06 | | |  |
| Necroptosis | | LIHC | | | GLS2 | | | -0.2037144 | | | 7.41E-05 | | |  |
| Necroptosis | | LIHC | | | LPCAT3 | | | -0.1592053 | | | 0.00206388 | | |  |
| Necroptosis | | LUAD | | | CDKN1A | | | 0.20506753 | | | 2.58E-06 | | |  |
| Necroptosis | | LUAD | | | TTC35 | | | -0.1355046 | | | 0.0020161 | | |  |
| Necroptosis | | LUAD | | | FANCD2 | | | 0.41866249 | | | 2.34E-23 | | |  |
| Necroptosis | | LUAD | | | CISD1 | | | 0.1547183 | | | 0.00041447 | | |  |
| Necroptosis | | LUAD | | | FDFT1 | | | -0.1490402 | | | 0.00067479 | | |  |
| Necroptosis | | LUAD | | | CARS | | | 0.23257296 | | | 8.85E-08 | | |  |
| Necroptosis | | LUAD | | | RRM2 | | | 0.61396203 | | | 7.02E-55 | | |  |
| Necroptosis | | LUAD | | | GLS2 | | | -0.4677035 | | | 1.85E-29 | | |  |
| Necroptosis | | LUAD | | | LPCAT3 | | | -0.1976908 | | | 5.93E-06 | | |  |
| Necroptosis | | LUSC | | | CDKN1A | | | 0.29914784 | | | 7.75E-12 | | |  |
| Necroptosis | | LUSC | | | TTC35 | | | 0.14311464 | | | 0.00130396 | | |  |
| Necroptosis | | LUSC | | | NFE2L2 | | | 0.28948404 | | | 3.79E-11 | | |  |
| Necroptosis | | LUSC | | | FDFT1 | | | 0.21143407 | | | 1.76E-06 | | |  |
| Necroptosis | | LUSC | | | LPCAT3 | | | 0.2530063 | | | 9.01E-09 | | |  |
| Necroptosis | | MESO | | | NFE2L2 | | | -0.3283152 | | | 0.00200249 | | |  |
| Necroptosis | | MESO | | | FANCD2 | | | 0.22681709 | | | 0.03484101 | | |  |
| Necroptosis | | MESO | | | CARS | | | 0.39658818 | | | 0.00016151 | | |  |
| Necroptosis | | MESO | | | RRM2 | | | 0.39088358 | | | 0.00020345 | | |  |
| Necroptosis | | OV | | | CISD1 | | | -0.12238 | | | 0.03184019 | | |  |
| Necroptosis | | OV | | | FDFT1 | | | -0.1413963 | | | 0.01305248 | | |  |
| Necroptosis | | PAAD | | | CDKN1A | | | 0.31976859 | | | 1.42E-05 | | |  |
| Necroptosis | | PAAD | | | TTC35 | | | 0.27373883 | | | 0.00022042 | | |  |
| Necroptosis | | PAAD | | | FANCD2 | | | 0.4391731 | | | 1.14E-09 | | |  |
| Necroptosis | | PAAD | | | CISD1 | | | -0.2403176 | | | 0.00122895 | | |  |
| Necroptosis | | PAAD | | | FDFT1 | | | 0.24767225 | | | 0.00085795 | | |  |
| Necroptosis | | PAAD | | | CARS | | | 0.40346285 | | | 2.87E-08 | | |  |
| Necroptosis | | PAAD | | | RRM2 | | | 0.51543741 | | | 0 | | |  |
| Necroptosis | | PAAD | | | GLS2 | | | -0.4613542 | | | 1.14E-10 | | |  |
| Necroptosis | | PCPG | | | CDKN1A | | | 0.18397835 | | | 0.01251638 | | |  |
| Necroptosis | | PCPG | | | TTC35 | | | -0.3252798 | | | 7.46E-06 | | |  |
| Necroptosis | | PCPG | | | NFE2L2 | | | 0.22236135 | | | 0.00246558 | | |  |
| Necroptosis | | PCPG | | | FDFT1 | | | -0.1735008 | | | 0.01860753 | | |  |
| Necroptosis | | PCPG | | | CARS | | | -0.1772803 | | | 0.01616415 | | |  |
| Necroptosis | | PCPG | | | GLS2 | | | -0.4467666 | | | 3.03E-10 | | |  |
| Necroptosis | | PRAD | | | FANCD2 | | | 0.21960403 | | | 7.46E-07 | | |  |
| Necroptosis | | PRAD | | | CISD1 | | | -0.1440289 | | | 0.0012691 | | |  |
| Necroptosis | | PRAD | | | FDFT1 | | | 0.21752375 | | | 9.54E-07 | | |  |
| Necroptosis | | PRAD | | | RRM2 | | | 0.10223696 | | | 0.02250354 | | |  |
| Necroptosis | | PRAD | | | GLS2 | | | -0.0921739 | | | 0.03976755 | | |  |
| Necroptosis | | PRAD | | | LPCAT3 | | | 0.16446935 | | | 0.00022763 | | |  |
| Necroptosis | | READ | | | CDKN1A | | | 0.23274385 | | | 0.00303174 | | |  |
| Necroptosis | | SARC | | | NFE2L2 | | | -0.1441368 | | | 0.01935635 | | |  |
| Necroptosis | | SARC | | | FANCD2 | | | 0.30502094 | | | 5.21E-07 | | |  |
| Necroptosis | | SARC | | | FDFT1 | | | 0.20562862 | | | 0.00081277 | | |  |
| Necroptosis | | SARC | | | RRM2 | | | 0.39262051 | | | 5.65E-11 | | |  |
| Necroptosis | | SARC | | | LPCAT3 | | | -0.1722063 | | | 0.00515438 | | |  |
| Necroptosis | | SKCM | | | CDKN1A | | | 0.14208642 | | | 0.00196731 | | |  |
| Necroptosis | | SKCM | | | TTC35 | | | -0.2245082 | | | 8.10E-07 | | |  |
| Necroptosis | | SKCM | | | RRM2 | | | 0.16921155 | | | 0.00022245 | | |  |
| Necroptosis | | SKCM | | | LPCAT3 | | | 0.1369313 | | | 0.00286374 | | |  |
| Necroptosis | | STAD | | | CDKN1A | | | 0.34372678 | | | 5.95E-13 | | |  |
| Necroptosis | | STAD | | | FANCD2 | | | 0.21596353 | | | 9.05E-06 | | |  |
| Necroptosis | | STAD | | | CISD1 | | | -0.1151759 | | | 0.01892268 | | |  |
| Necroptosis | | STAD | | | FDFT1 | | | 0.20806966 | | | 1.93E-05 | | |  |
| Necroptosis | | STAD | | | CARS | | | 0.21815592 | | | 7.29E-06 | | |  |
| Necroptosis | | STAD | | | RRM2 | | | 0.21982261 | | | 6.18E-06 | | |  |
| Necroptosis | | TGCT | | | CDKN1A | | | 0.52427494 | | | 0 | | |  |
| Necroptosis | | TGCT | | | FANCD2 | | | -0.1890029 | | | 0.01824817 | | |  |
| Necroptosis | | TGCT | | | CISD1 | | | 0.30135449 | | | 0.00014135 | | |  |
| Necroptosis | | TGCT | | | FDFT1 | | | -0.2401732 | | | 0.00258924 | | |  |
| Necroptosis | | TGCT | | | CARS | | | 0.15759827 | | | 0.04951299 | | |  |
| Necroptosis | | TGCT | | | RRM2 | | | 0.68967774 | | | 0 | | |  |
| Necroptosis | | TGCT | | | GLS2 | | | 0.50695263 | | | 5.12E-12 | | |  |
| Necroptosis | | TGCT | | | LPCAT3 | | | 0.48399741 | | | 2.18E-10 | | |  |
| Necroptosis | | THCA | | | CDKN1A | | | 0.29309346 | | | 1.59E-11 | | |  |
| Necroptosis | | THCA | | | TTC35 | | | -0.1464163 | | | 0.00089067 | | |  |
| Necroptosis | | THCA | | | FANCD2 | | | 0.32295035 | | | 8.68E-14 | | |  |
| Necroptosis | | THCA | | | CISD1 | | | -0.097983 | | | 0.02647347 | | |  |
| Necroptosis | | THCA | | | FDFT1 | | | -0.1476878 | | | 0.00079275 | | |  |
| Necroptosis | | THCA | | | CARS | | | -0.2387116 | | | 4.43E-08 | | |  |
| Necroptosis | | THCA | | | RRM2 | | | 0.20726369 | | | 2.31E-06 | | |  |
| Necroptosis | | THCA | | | GLS2 | | | 0.32921216 | | | 2.66E-14 | | |  |
| Necroptosis | | THYM | | | CDKN1A | | | 0.40156261 | | | 6.60E-06 | | |  |
| Necroptosis | | THYM | | | NFE2L2 | | | 0.19747205 | | | 0.0307782 | | |  |
| Necroptosis | | THYM | | | CISD1 | | | 0.26380999 | | | 0.00369389 | | |  |
| Necroptosis | | THYM | | | CARS | | | 0.24795472 | | | 0.00644502 | | |  |
| Necroptosis | | THYM | | | GLS2 | | | 0.21191749 | | | 0.02030675 | | |  |
| Necroptosis | | THYM | | | LPCAT3 | | | 0.34155844 | | | 0.00014719 | | |  |
| Necroptosis | | UCEC | | | NFE2L2 | | | 0.12729206 | | | 0.00326142 | | |  |
| Necroptosis | | UCEC | | | FANCD2 | | | 0.21209302 | | | 8.24E-07 | | |  |
| Necroptosis | | UCEC | | | CARS | | | 0.1513378 | | | 0.00046137 | | |  |
| Necroptosis | | UCEC | | | RRM2 | | | 0.21416834 | | | 6.40E-07 | | |  |
| Necroptosis | | UCEC | | | LPCAT3 | | | -0.136158 | | | 0.00164229 | | |  |
| Necroptosis | | UCS | | | CDKN1A | | | -0.3098911 | | | 0.01934765 | | |  |
| Necroptosis | | UCS | | | GLS2 | | | 0.42500648 | | | 0.0010867 | | |  |
| Necroptosis | | UCS | | | LPCAT3 | | | 0.38682914 | | | 0.0031472 | | |  |
| Necroptosis | | UVM | | | CDKN1A | | | 0.47998125 | | | 8.65E-06 | | |  |
| Necroptosis | | UVM | | | NFE2L2 | | | -0.4384904 | | | 5.64E-05 | | |  |
| Necroptosis | | UVM | | | FANCD2 | | | -0.2626582 | | | 0.01883015 | | |  |
| Necroptosis | | UVM | | | LPCAT3 | | | 0.30067979 | | | 0.00691994 | | |  |
| Necroptosis | | ACC | | | CDCA8 | | | 0.4400925 | | | 5.89E-05 | | |  |
| Necroptosis | | ACC | | | SPP1 | | | 0.31380234 | | | 0.00503081 | | |  |
| Necroptosis | | ACC | | | MMP1 | | | 0.38387447 | | | 0.00047873 | | |  |
| Necroptosis | | BLCA | | | MTMR2 | | | 0.15033954 | | | 0.00235099 | | |  |
| Necroptosis | | BLCA | | | S100A9 | | | 0.30560368 | | | 3.55E-10 | | |  |
| Necroptosis | | BLCA | | | ANXA10 | | | 0.17572161 | | | 0.00036206 | | |  |
| Necroptosis | | BLCA | | | G6PD | | | 0.25518902 | | | 1.92E-07 | | |  |
| Necroptosis | | BLCA | | | SLC1A5 | | | 0.20666543 | | | 2.69E-05 | | |  |
| Necroptosis | | BLCA | | | SPP1 | | | 0.15043124 | | | 0.00233642 | | |  |
| Necroptosis | | BLCA | | | PLOD2 | | | 0.22577973 | | | 4.35E-06 | | |  |
| Necroptosis | | BLCA | | | MMP1 | | | 0.23659506 | | | 1.44E-06 | | |  |
| Necroptosis | | BRCA | | | MTMR2 | | | 0.11502163 | | | 0.00012806 | | |  |
| Necroptosis | | BRCA | | | CDCA8 | | | 0.36580144 | | | 2.75E-36 | | |  |
| Necroptosis | | BRCA | | | S100A9 | | | 0.15594389 | | | 1.91E-07 | | |  |
| Necroptosis | | BRCA | | | G6PD | | | 0.23025505 | | | 9.47E-15 | | |  |
| Necroptosis | | BRCA | | | SLC1A5 | | | 0.17979551 | | | 1.78E-09 | | |  |
| Necroptosis | | BRCA | | | SPP1 | | | 0.17565555 | | | 4.21E-09 | | |  |
| Necroptosis | | BRCA | | | PLOD2 | | | 0.19898621 | | | 2.54E-11 | | |  |
| Necroptosis | | BRCA | | | MMP1 | | | 0.24366492 | | | 2.19E-16 | | |  |
| Necroptosis | | CESC | | | CDCA8 | | | 0.16947195 | | | 0.0028933 | | |  |
| Necroptosis | | CESC | | | S100A9 | | | 0.5112008 | | | 7.69E-22 | | |  |
| Necroptosis | | CESC | | | ANXA10 | | | -0.181897 | | | 0.0013703 | | |  |
| Necroptosis | | CESC | | | G6PD | | | 0.16079984 | | | 0.00473767 | | |  |
| Necroptosis | | CESC | | | SPP1 | | | 0.32333099 | | | 6.69E-09 | | |  |
| Necroptosis | | CESC | | | PLOD2 | | | 0.24664337 | | | 1.23E-05 | | |  |
| Necroptosis | | CESC | | | MMP1 | | | 0.19425942 | | | 0.00062054 | | |  |
| Necroptosis | | CHOL | | | MTMR2 | | | 0.38301158 | | | 0.02177483 | | |  |
| Necroptosis | | CHOL | | | S100A9 | | | 0.48314028 | | | 0.00315589 | | |  |
| Necroptosis | | CHOL | | | G6PD | | | 0.43835264 | | | 0.00798939 | | |  |
| Necroptosis | | CHOL | | | SLC1A5 | | | 0.52303732 | | | 0.00124576 | | |  |
| Necroptosis | | CHOL | | | MMP1 | | | 0.55032175 | | | 0.00061933 | | |  |
| Necroptosis | | COAD | | | S100A9 | | | 0.15748214 | | | 0.0007694 | | |  |
| Necroptosis | | COAD | | | ANXA10 | | | 0.1385514 | | | 0.01865139 | | |  |
| Necroptosis | | COAD | | | G6PD | | | 0.24565224 | | | 1.27E-07 | | |  |
| Necroptosis | | COAD | | | SLC1A5 | | | 0.26257001 | | | 1.54E-08 | | |  |
| Necroptosis | | COAD | | | SPP1 | | | 0.10109826 | | | 0.03126286 | | |  |
| Necroptosis | | COAD | | | MMP1 | | | 0.10409703 | | | 0.0265981 | | |  |
| Necroptosis | | ESCA | | | S100A9 | | | 0.55397635 | | | 0 | | |  |
| Necroptosis | | ESCA | | | ANXA10 | | | -0.370666 | | | 2.06E-07 | | |  |
| Necroptosis | | ESCA | | | G6PD | | | 0.46164285 | | | 5.05E-11 | | |  |
| Necroptosis | | ESCA | | | SLC1A5 | | | 0.26562299 | | | 0.00027062 | | |  |
| Necroptosis | | ESCA | | | SPP1 | | | 0.32312081 | | | 8.14E-06 | | |  |
| Necroptosis | | ESCA | | | PLOD2 | | | 0.32007884 | | | 9.98E-06 | | |  |
| Necroptosis | | ESCA | | | MMP1 | | | 0.19527501 | | | 0.007812 | | |  |
| Necroptosis | | GBM | | | S100A9 | | | 0.25236017 | | | 0.00103526 | | |  |
| Necroptosis | | GBM | | | ANXA10 | | | 0.19604402 | | | 0.01111359 | | |  |
| Necroptosis | | GBM | | | G6PD | | | 0.17648696 | | | 0.02262964 | | |  |
| Necroptosis | | GBM | | | SPP1 | | | 0.28011018 | | | 0.00025972 | | |  |
| Necroptosis | | GBM | | | PLOD2 | | | 0.47903161 | | | 7.59E-11 | | |  |
| Necroptosis | | GBM | | | MMP1 | | | 0.16245731 | | | 0.03594069 | | |  |
| Necroptosis | | HNSC | | | MTMR2 | | | 0.19419653 | | | 8.19E-06 | | |  |
| Necroptosis | | HNSC | | | S100A9 | | | 0.18191087 | | | 2.99E-05 | | |  |
| Necroptosis | | HNSC | | | G6PD | | | 0.24347776 | | | 1.95E-08 | | |  |
| Necroptosis | | HNSC | | | PLOD2 | | | 0.10691381 | | | 0.01456565 | | |  |
| Necroptosis | | HNSC | | | MMP1 | | | 0.25056094 | | | 7.31E-09 | | |  |
| Necroptosis | | KICH | | | CDCA8 | | | 0.46631876 | | | 9.56E-05 | | |  |
| Necroptosis | | KICH | | | SLC1A5 | | | 0.36971089 | | | 0.00239166 | | |  |
| Necroptosis | | KICH | | | SPP1 | | | 0.363031 | | | 0.00289478 | | |  |
| Necroptosis | | KICH | | | PLOD2 | | | 0.41855756 | | | 0.00052533 | | |  |
| Necroptosis | | KIRC | | | MTMR2 | | | 0.10167705 | | | 0.01879807 | | |  |
| Necroptosis | | KIRC | | | CDCA8 | | | 0.20500762 | | | 1.78E-06 | | |  |
| Necroptosis | | KIRC | | | S100A9 | | | 0.13069702 | | | 0.00249447 | | |  |
| Necroptosis | | KIRC | | | ANXA10 | | | -0.1582558 | | | 0.000241 | | |  |
| Necroptosis | | KIRC | | | SLC1A5 | | | 0.12361536 | | | 0.00424756 | | |  |
| Necroptosis | | KIRC | | | SPP1 | | | 0.28432378 | | | 2.68E-11 | | |  |
| Necroptosis | | KIRC | | | PLOD2 | | | 0.5426117 | | | 0 | | |  |
| Necroptosis | | KIRC | | | MMP1 | | | 0.15291043 | | | 0.00039095 | | |  |
| Necroptosis | | KIRP | | | MTMR2 | | | -0.2351017 | | | 5.37E-05 | | |  |
| Necroptosis | | KIRP | | | CDCA8 | | | 0.38110977 | | | 1.71E-11 | | |  |
| Necroptosis | | KIRP | | | S100A9 | | | 0.27027061 | | | 3.16E-06 | | |  |
| Necroptosis | | KIRP | | | G6PD | | | 0.4578873 | | | 0 | | |  |
| Necroptosis | | KIRP | | | SLC1A5 | | | 0.16930932 | | | 0.00381033 | | |  |
| Necroptosis | | KIRP | | | SPP1 | | | 0.33014965 | | | 9.73E-09 | | |  |
| Necroptosis | | KIRP | | | PLOD2 | | | 0.34653638 | | | 1.59E-09 | | |  |
| Necroptosis | | KIRP | | | MMP1 | | | 0.39248188 | | | 3.73E-12 | | |  |
| Necroptosis | | LGG | | | S100A9 | | | 0.11443821 | | | 0.00836395 | | |  |
| Necroptosis | | LIHC | | | MTMR2 | | | 0.36220639 | | | 7.23E-13 | | |  |
| Necroptosis | | LIHC | | | CDCA8 | | | 0.2511607 | | | 9.74E-07 | | |  |
| Necroptosis | | LIHC | | | S100A9 | | | 0.32155696 | | | 2.57E-10 | | |  |
| Necroptosis | | LIHC | | | ANXA10 | | | -0.2615945 | | | 2.98E-07 | | |  |
| Necroptosis | | LIHC | | | G6PD | | | 0.43657415 | | | 0 | | |  |
| Necroptosis | | LIHC | | | SLC1A5 | | | 0.39889635 | | | 2.20E-16 | | |  |
| Necroptosis | | LIHC | | | SPP1 | | | 0.48795207 | | | 0 | | |  |
| Necroptosis | | LIHC | | | PLOD2 | | | 0.19632693 | | | 0.00013949 | | |  |
| Necroptosis | | LIHC | | | MMP1 | | | 0.36093596 | | | 6.42E-13 | | |  |
| Necroptosis | | LUAD | | | MTMR2 | | | 0.35590603 | | | 6.96E-17 | | |  |
| Necroptosis | | LUAD | | | CDCA8 | | | 0.59402865 | | | 1.26E-50 | | |  |
| Necroptosis | | LUAD | | | S100A9 | | | 0.24709223 | | | 1.25E-08 | | |  |
| Necroptosis | | LUAD | | | ANXA10 | | | 0.15481282 | | | 0.00041106 | | |  |
| Necroptosis | | LUAD | | | G6PD | | | 0.20235883 | | | 3.52E-06 | | |  |
| Necroptosis | | LUAD | | | SPP1 | | | 0.38150196 | | | 2.34E-19 | | |  |
| Necroptosis | | LUAD | | | PLOD2 | | | 0.5895038 | | | 1.06E-49 | | |  |
| Necroptosis | | LUAD | | | MMP1 | | | 0.3128619 | | | 3.34E-13 | | |  |
| Necroptosis | | LUSC | | | MTMR2 | | | 0.20417202 | | | 3.99E-06 | | |  |
| Necroptosis | | LUSC | | | CDCA8 | | | 0.13304756 | | | 0.00281881 | | |  |
| Necroptosis | | LUSC | | | S100A9 | | | 0.25558481 | | | 6.29E-09 | | |  |
| Necroptosis | | LUSC | | | ANXA10 | | | 0.27313347 | | | 4.87E-10 | | |  |
| Necroptosis | | LUSC | | | G6PD | | | 0.23296812 | | | 1.30E-07 | | |  |
| Necroptosis | | LUSC | | | SLC1A5 | | | 0.26334268 | | | 2.08E-09 | | |  |
| Necroptosis | | LUSC | | | SPP1 | | | 0.16160921 | | | 0.00027711 | | |  |
| Necroptosis | | LUSC | | | PLOD2 | | | 0.24136547 | | | 4.36E-08 | | |  |
| Necroptosis | | LUSC | | | MMP1 | | | 0.18654668 | | | 2.60E-05 | | |  |
| Necroptosis | | MESO | | | CDCA8 | | | 0.46466064 | | | 7.45E-06 | | |  |
| Necroptosis | | MESO | | | SLC1A5 | | | 0.33797478 | | | 0.00144702 | | |  |
| Necroptosis | | MESO | | | SPP1 | | | 0.43473427 | | | 3.11E-05 | | |  |
| Necroptosis | | MESO | | | PLOD2 | | | 0.32102501 | | | 0.0025424 | | |  |
| Necroptosis | | MESO | | | MMP1 | | | 0.40347016 | | | 0.00010664 | | |  |
| Necroptosis | | OV | | | MTMR2 | | | 0.12035484 | | | 0.03479881 | | |  |
| Necroptosis | | OV | | | CDCA8 | | | 0.17689401 | | | 0.00183032 | | |  |
| Necroptosis | | OV | | | SPP1 | | | 0.13206249 | | | 0.02048578 | | |  |
| Necroptosis | | OV | | | PLOD2 | | | 0.22224175 | | | 8.70E-05 | | |  |
| Necroptosis | | OV | | | MMP1 | | | 0.19438019 | | | 0.000603 | | |  |
| Necroptosis | | PAAD | | | MTMR2 | | | 0.29975519 | | | 4.95E-05 | | |  |
| Necroptosis | | PAAD | | | CDCA8 | | | 0.55537213 | | | 0 | | |  |
| Necroptosis | | PAAD | | | ANXA10 | | | 0.27611784 | | | 0.0001934 | | |  |
| Necroptosis | | PAAD | | | G6PD | | | 0.27833574 | | | 0.00017102 | | |  |
| Necroptosis | | PAAD | | | SLC1A5 | | | 0.36218484 | | | 7.56E-07 | | |  |
| Necroptosis | | PAAD | | | PLOD2 | | | 0.35111 | | | 1.69E-06 | | |  |
| Necroptosis | | PCPG | | | CDCA8 | | | 0.21957774 | | | 0.00279981 | | |  |
| Necroptosis | | PCPG | | | S100A9 | | | 0.2389089 | | | 0.00112281 | | |  |
| Necroptosis | | PCPG | | | G6PD | | | -0.2468956 | | | 0.00075352 | | |  |
| Necroptosis | | PCPG | | | SPP1 | | | 0.28449847 | | | 9.69E-05 | | |  |
| Necroptosis | | PCPG | | | PLOD2 | | | 0.30156036 | | | 3.46E-05 | | |  |
| Necroptosis | | PCPG | | | MMP1 | | | 0.34535159 | | | 1.58E-06 | | |  |
| Necroptosis | | PRAD | | | CDCA8 | | | 0.10497493 | | | 0.01911903 | | |  |
| Necroptosis | | PRAD | | | ANXA10 | | | -0.1225569 | | | 0.00617341 | | |  |
| Necroptosis | | PRAD | | | G6PD | | | 0.1452859 | | | 0.00114876 | | |  |
| Necroptosis | | PRAD | | | SLC1A5 | | | 0.28679721 | | | 6.96E-11 | | |  |
| Necroptosis | | READ | | | MTMR2 | | | 0.17039913 | | | 0.03079751 | | |  |
| Necroptosis | | READ | | | G6PD | | | 0.26080343 | | | 0.00086483 | | |  |
| Necroptosis | | READ | | | SLC1A5 | | | 0.29647458 | | | 0.0001434 | | |  |
| Necroptosis | | SARC | | | CDCA8 | | | 0.32192935 | | | 1.11E-07 | | |  |
| Necroptosis | | SARC | | | G6PD | | | 0.17390754 | | | 0.00472536 | | |  |
| Necroptosis | | SARC | | | SLC1A5 | | | 0.18712779 | | | 0.00234395 | | |  |
| Necroptosis | | SARC | | | SPP1 | | | 0.26002618 | | | 2.08E-05 | | |  |
| Necroptosis | | SARC | | | PLOD2 | | | 0.43128056 | | | 1.95E-13 | | |  |
| Necroptosis | | SARC | | | MMP1 | | | 0.22731598 | | | 0.00020107 | | |  |
| Necroptosis | | SKCM | | | CDCA8 | | | 0.22993312 | | | 4.63E-07 | | |  |
| Necroptosis | | SKCM | | | S100A9 | | | 0.26966642 | | | 2.52E-09 | | |  |
| Necroptosis | | SKCM | | | G6PD | | | 0.19628123 | | | 1.71E-05 | | |  |
| Necroptosis | | SKCM | | | SLC1A5 | | | 0.33216293 | | | 1.63E-13 | | |  |
| Necroptosis | | SKCM | | | MMP1 | | | 0.24136148 | | | 1.07E-07 | | |  |
| Necroptosis | | STAD | | | CDCA8 | | | 0.24751692 | | | 3.28E-07 | | |  |
| Necroptosis | | STAD | | | S100A9 | | | 0.27472047 | | | 1.27E-08 | | |  |
| Necroptosis | | STAD | | | G6PD | | | 0.23368399 | | | 1.49E-06 | | |  |
| Necroptosis | | STAD | | | SLC1A5 | | | 0.40765381 | | | 4.78E-18 | | |  |
| Necroptosis | | STAD | | | SPP1 | | | 0.19772192 | | | 4.99E-05 | | |  |
| Necroptosis | | STAD | | | MMP1 | | | 0.28462633 | | | 1.86E-08 | | |  |
| Necroptosis | | TGCT | | | MTMR2 | | | 0.43854847 | | | 1.47E-08 | | |  |
| Necroptosis | | TGCT | | | CDCA8 | | | 0.54788134 | | | 0 | | |  |
| Necroptosis | | TGCT | | | S100A9 | | | 0.33564824 | | | 2.06E-05 | | |  |
| Necroptosis | | TGCT | | | SLC1A5 | | | 0.53160216 | | | 0 | | |  |
| Necroptosis | | TGCT | | | SPP1 | | | 0.62114713 | | | 0 | | |  |
| Necroptosis | | TGCT | | | PLOD2 | | | 0.4249593 | | | 4.41E-08 | | |  |
| Necroptosis | | TGCT | | | MMP1 | | | 0.47664491 | | | 4.73E-10 | | |  |
| Necroptosis | | THCA | | | CDCA8 | | | 0.14880593 | | | 0.00073107 | | |  |
| Necroptosis | | THCA | | | S100A9 | | | 0.33701648 | | | 5.73E-15 | | |  |
| Necroptosis | | THCA | | | SLC1A5 | | | 0.33225613 | | | 1.47E-14 | | |  |
| Necroptosis | | THCA | | | SPP1 | | | 0.31872349 | | | 1.89E-13 | | |  |
| Necroptosis | | THCA | | | MMP1 | | | 0.29491953 | | | 9.38E-12 | | |  |
| Necroptosis | | THYM | | | S100A9 | | | 0.3719425 | | | 3.28E-05 | | |  |
| Necroptosis | | THYM | | | SPP1 | | | 0.23004375 | | | 0.01163156 | | |  |
| Necroptosis | | THYM | | | MMP1 | | | 0.43891132 | | | 5.32E-07 | | |  |
| Necroptosis | | UCEC | | | MTMR2 | | | 0.09708381 | | | 0.02503445 | | |  |
| Necroptosis | | UCEC | | | CDCA8 | | | 0.30039438 | | | 1.81E-12 | | |  |
| Necroptosis | | UCEC | | | S100A9 | | | 0.22002083 | | | 2.89E-07 | | |  |
| Necroptosis | | UCEC | | | ANXA10 | | | -0.1541024 | | | 0.04056761 | | |  |
| Necroptosis | | UCEC | | | G6PD | | | 0.13368709 | | | 0.00199647 | | |  |
| Necroptosis | | UCEC | | | SPP1 | | | 0.26223938 | | | 9.10E-10 | | |  |
| Necroptosis | | UCEC | | | PLOD2 | | | 0.13213861 | | | 0.00225275 | | |  |
| Necroptosis | | UCEC | | | MMP1 | | | 0.37136287 | | | 7.14E-19 | | |  |
| Necroptosis | | UCS | | | S100A9 | | | 0.34093855 | | | 0.00976676 | | |  |
| Necroptosis | | UCS | | | SPP1 | | | 0.36874514 | | | 0.0050075 | | |  |
| Necroptosis | | UCS | | | PLOD2 | | | 0.26963962 | | | 0.04283601 | | |  |
| Necroptosis | | UCS | | | MMP1 | | | 0.26750065 | | | 0.04456117 | | |  |
| Necroptosis | | UVM | | | MTMR2 | | | -0.2334519 | | | 0.0371524 | | |  |
| Necroptosis | | UVM | | | G6PD | | | 0.4543835 | | | 2.82E-05 | | |  |
| Autophagy | ACC | | | NLRC4 | | | -0.3474197 | | | 0.00180758 | | |  |  |
| Autophagy | ACC | | | MAPK1 | | | 0.35287244 | | | 0.00151541 | | |  |  |
| Autophagy | ACC | | | RAB24 | | | -0.2772395 | | | 0.01361654 | | |  |  |
| Autophagy | BLCA | | | NLRC4 | | | -0.2171007 | | | 1.02E-05 | | |  |  |
| Autophagy | BLCA | | | MAPK1 | | | 0.1894703 | | | 0.00012124 | | |  |  |
| Autophagy | BLCA | | | ATG9B | | | 0.19098079 | | | 0.00010676 | | |  |  |
| Autophagy | BLCA | | | TSC1 | | | -0.2096325 | | | 2.05E-05 | | |  |  |
| Autophagy | BLCA | | | RAB24 | | | -0.1052036 | | | 0.03367827 | | |  |  |
| Autophagy | BRCA | | | ULK2 | | | -0.1101447 | | | 0.00024567 | | |  |  |
| Autophagy | BRCA | | | NLRC4 | | | -0.1119561 | | | 0.00019345 | | |  |  |
| Autophagy | BRCA | | | ATG4D | | | 0.10325722 | | | 0.00058998 | | |  |  |
| Autophagy | BRCA | | | ATG9B | | | 0.16205778 | | | 6.15E-08 | | |  |  |
| Autophagy | BRCA | | | FOXO1 | | | -0.1478235 | | | 8.09E-07 | | |  |  |
| Autophagy | BRCA | | | TSC1 | | | -0.1684851 | | | 1.78E-08 | | |  |  |
| Autophagy | BRCA | | | RAB24 | | | -0.1469807 | | | 9.35E-07 | | |  |  |
| Autophagy | CESC | | | MAPK1 | | | 0.20323491 | | | 0.00033849 | | |  |  |
| Autophagy | CESC | | | ATG4D | | | 0.1635259 | | | 0.00406748 | | |  |  |
| Autophagy | CESC | | | FOXO1 | | | 0.11933131 | | | 0.03663603 | | |  |  |
| Autophagy | CESC | | | NRG2 | | | -0.1687601 | | | 0.00301546 | | |  |  |
| Autophagy | CESC | | | RAB24 | | | 0.1731519 | | | 0.00233053 | | |  |  |
| Autophagy | COAD | | | ULK2 | | | -0.1769506 | | | 0.00015072 | | |  |  |
| Autophagy | COAD | | | ATG2A | | | 0.18146229 | | | 0.00010102 | | |  |  |
| Autophagy | COAD | | | ATG9B | | | 0.19402297 | | | 3.26E-05 | | |  |  |
| Autophagy | COAD | | | TSC1 | | | -0.1848151 | | | 7.67E-05 | | |  |  |
| Autophagy | ESCA | | | ULK2 | | | 0.2944695 | | | 5.10E-05 | | |  |  |
| Autophagy | ESCA | | | NLRC4 | | | -0.2132216 | | | 0.00362858 | | |  |  |
| Autophagy | ESCA | | | MAPK1 | | | 0.38330996 | | | 9.22E-08 | | |  |  |
| Autophagy | ESCA | | | ATG4D | | | 0.37932603 | | | 1.28E-07 | | |  |  |
| Autophagy | ESCA | | | ATG2A | | | -0.2264167 | | | 0.00198589 | | |  |  |
| Autophagy | ESCA | | | ATG9B | | | 0.17483037 | | | 0.01740393 | | |  |  |
| Autophagy | ESCA | | | NRG2 | | | -0.1868332 | | | 0.01088263 | | |  |  |
| Autophagy | ESCA | | | TSC1 | | | -0.2149198 | | | 0.00336402 | | |  |  |
| Autophagy | ESCA | | | RAB24 | | | 0.14690307 | | | 0.04607467 | | |  |  |
| Autophagy | HNSC | | | NLRC4 | | | -0.3257988 | | | 2.26E-14 | | |  |  |
| Autophagy | HNSC | | | MAPK1 | | | 0.24585454 | | | 1.41E-08 | | |  |  |
| Autophagy | HNSC | | | ATG4D | | | -0.1477336 | | | 0.00071825 | | |  |  |
| Autophagy | HNSC | | | ATG2A | | | 0.09367228 | | | 0.03240801 | | |  |  |
| Autophagy | HNSC | | | NRG2 | | | -0.2054733 | | | 2.20E-06 | | |  |  |
| Autophagy | HNSC | | | RAB24 | | | -0.1362098 | | | 0.00182892 | | |  |  |
| Autophagy | KICH | | | ATG2A | | | -0.2923912 | | | 0.01750863 | | |  |  |
| Autophagy | KICH | | | FOXO1 | | | -0.3854504 | | | 0.00150227 | | |  |  |
| Autophagy | KIRC | | | ATG4D | | | -0.2884661 | | | 1.08E-11 | | |  |  |
| Autophagy | KIRC | | | ATG9B | | | 0.21545519 | | | 5.00E-07 | | |  |  |
| Autophagy | KIRC | | | NRG2 | | | -0.1626202 | | | 0.00016049 | | |  |  |
| Autophagy | KIRC | | | TSC1 | | | -0.2837695 | | | 2.39E-11 | | |  |  |
| Autophagy | KIRP | | | ULK2 | | | -0.2475347 | | | 2.07E-05 | | |  |  |
| Autophagy | KIRP | | | MAPK1 | | | 0.1319948 | | | 0.02439532 | | |  |  |
| Autophagy | KIRP | | | ATG4D | | | -0.3695859 | | | 1.03E-10 | | |  |  |
| Autophagy | KIRP | | | ATG2A | | | 0.21760807 | | | 0.00018954 | | |  |  |
| Autophagy | KIRP | | | ATG9B | | | 0.33394124 | | | 6.46E-09 | | |  |  |
| Autophagy | KIRP | | | FOXO1 | | | 0.17393608 | | | 0.00294479 | | |  |  |
| Autophagy | KIRP | | | TSC1 | | | -0.1247106 | | | 0.03351441 | | |  |  |
| Autophagy | KIRP | | | RAB24 | | | -0.1526582 | | | 0.0091561 | | |  |  |
| Autophagy | LGG | | | FOXO1 | | | 0.27954461 | | | 5.70E-11 | | |  |  |
| Autophagy | LGG | | | TSC1 | | | -0.0942863 | | | 0.02998067 | | |  |  |
| Autophagy | LIHC | | | NLRC4 | | | 0.27530372 | | | 7.39E-08 | | |  |  |
| Autophagy | LIHC | | | ATG9B | | | 0.36719781 | | | 2.38E-13 | | |  |  |
| Autophagy | LIHC | | | FOXO1 | | | -0.1258918 | | | 0.01502647 | | |  |  |
| Autophagy | LIHC | | | NRG2 | | | 0.31149551 | | | 7.78E-10 | | |  |  |
| Autophagy | LIHC | | | RAB24 | | | 0.10369074 | | | 0.04536257 | | |  |  |
| Autophagy | LUAD | | | ULK2 | | | -0.3178838 | | | 1.33E-13 | | |  |  |
| Autophagy | LUAD | | | ATG2A | | | -0.156733 | | | 0.00034724 | | |  |  |
| Autophagy | LUAD | | | ATG9B | | | 0.16635359 | | | 0.00014479 | | |  |  |
| Autophagy | LUAD | | | FOXO1 | | | -0.1297064 | | | 0.00313107 | | |  |  |
| Autophagy | LUAD | | | NRG2 | | | -0.1599055 | | | 0.00026164 | | |  |  |
| Autophagy | LUAD | | | TSC1 | | | -0.271527 | | | 3.45E-10 | | |  |  |
| Autophagy | LUAD | | | RAB24 | | | -0.1276182 | | | 0.00365374 | | |  |  |
| Autophagy | LUSC | | | NLRC4 | | | -0.3263318 | | | 6.40E-14 | | |  |  |
| Autophagy | LUSC | | | MAPK1 | | | 0.21674558 | | | 9.46E-07 | | |  |  |
| Autophagy | LUSC | | | ATG4D | | | 0.1780852 | | | 6.01E-05 | | |  |  |
| Autophagy | LUSC | | | ATG2A | | | -0.1047665 | | | 0.01887733 | | |  |  |
| Autophagy | LUSC | | | FOXO1 | | | 0.11087124 | | | 0.01293389 | | |  |  |
| Autophagy | LUSC | | | NRG2 | | | -0.3092426 | | | 1.38E-12 | | |  |  |
| Autophagy | LUSC | | | RAB24 | | | -0.1061372 | | | 0.0173674 | | |  |  |
| Autophagy | MESO | | | FOXO1 | | | -0.2148976 | | | 0.0458006 | | |  |  |
| Autophagy | MESO | | | NRG2 | | | -0.2569075 | | | 0.01652496 | | |  |  |
| Autophagy | OV | | | NRG2 | | | -0.1223575 | | | 0.03187197 | | |  |  |
| Autophagy | PAAD | | | ULK2 | | | -0.5744481 | | | 0 | | |  |  |
| Autophagy | PAAD | | | NLRC4 | | | -0.2418241 | | | 0.0011427 | | |  |  |
| Autophagy | PAAD | | | ATG9B | | | 0.3656707 | | | 5.83E-07 | | |  |  |
| Autophagy | PAAD | | | FOXO1 | | | -0.2216224 | | | 0.00292507 | | |  |  |
| Autophagy | PAAD | | | NRG2 | | | -0.4917926 | | | 2.73E-12 | | |  |  |
| Autophagy | PAAD | | | TSC1 | | | -0.2471073 | | | 0.00088229 | | |  |  |
| Autophagy | PCPG | | | ATG4D | | | -0.2151008 | | | 0.00342432 | | |  |  |
| Autophagy | PCPG | | | FOXO1 | | | 0.45014159 | | | 2.12E-10 | | |  |  |
| Autophagy | PRAD | | | MAPK1 | | | 0.19871219 | | | 7.90E-06 | | |  |  |
| Autophagy | PRAD | | | ATG2A | | | 0.32285051 | | | 1.52E-13 | | |  |  |
| Autophagy | PRAD | | | RAB24 | | | -0.1790643 | | | 5.86E-05 | | |  |  |
| Autophagy | READ | | | ATG9B | | | 0.22735507 | | | 0.00379845 | | |  |  |
| Autophagy | READ | | | RAB24 | | | -0.1573154 | | | 0.04634986 | | |  |  |
| Autophagy | SARC | | | NLRC4 | | | -0.1425275 | | | 0.020836 | | |  |  |
| Autophagy | SARC | | | MAPK1 | | | 0.15572598 | | | 0.01150845 | | |  |  |
| Autophagy | SARC | | | FOXO1 | | | -0.1988374 | | | 0.00121188 | | |  |  |
| Autophagy | SARC | | | NRG2 | | | -0.1695542 | | | 0.00584064 | | |  |  |
| Autophagy | SARC | | | TSC1 | | | -0.1243499 | | | 0.04397528 | | |  |  |
| Autophagy | SARC | | | RAB24 | | | -0.1261389 | | | 0.04100472 | | |  |  |
| Autophagy | SKCM | | | NLRC4 | | | -0.1676859 | | | 0.00024904 | | |  |  |
| Autophagy | SKCM | | | ATG4D | | | 0.1411281 | | | 0.00211148 | | |  |  |
| Autophagy | SKCM | | | ATG2A | | | 0.09514944 | | | 0.03861743 | | |  |  |
| Autophagy | SKCM | | | ATG9B | | | 0.42316274 | | | 0 | | |  |  |
| Autophagy | STAD | | | ULK2 | | | -0.1089244 | | | 0.02649544 | | |  |  |
| Autophagy | STAD | | | NLRC4 | | | -0.2390731 | | | 8.34E-07 | | |  |  |
| Autophagy | STAD | | | ATG4D | | | 0.17633352 | | | 0.00030658 | | |  |  |
| Autophagy | STAD | | | ATG2A | | | 0.18451189 | | | 0.00015685 | | |  |  |
| Autophagy | STAD | | | ATG9B | | | 0.32758074 | | | 7.76E-12 | | |  |  |
| Autophagy | STAD | | | NRG2 | | | -0.2324773 | | | 1.69E-06 | | |  |  |
| Autophagy | TGCT | | | ULK2 | | | -0.3426594 | | | 1.35E-05 | | |  |  |
| Autophagy | TGCT | | | MAPK1 | | | -0.3375006 | | | 1.84E-05 | | |  |  |
| Autophagy | TGCT | | | ATG2A | | | -0.6066855 | | | 0 | | |  |  |
| Autophagy | TGCT | | | ATG9B | | | 0.39507831 | | | 4.19E-07 | | |  |  |
| Autophagy | TGCT | | | FOXO1 | | | -0.4316037 | | | 2.59E-08 | | |  |  |
| Autophagy | TGCT | | | NRG2 | | | -0.500207 | | | 2.60E-11 | | |  |  |
| Autophagy | TGCT | | | TSC1 | | | -0.3898753 | | | 6.07E-07 | | |  |  |
| Autophagy | THCA | | | NLRC4 | | | 0.22357817 | | | 3.35E-07 | | |  |  |
| Autophagy | THCA | | | MAPK1 | | | 0.30708652 | | | 1.50E-12 | | |  |  |
| Autophagy | THCA | | | ATG4D | | | -0.361311 | | | 1.81E-17 | | |  |  |
| Autophagy | THCA | | | ATG2A | | | 0.32152792 | | | 1.13E-13 | | |  |  |
| Autophagy | THCA | | | ATG9B | | | 0.11860025 | | | 0.00716275 | | |  |  |
| Autophagy | THCA | | | FOXO1 | | | -0.2628878 | | | 1.71E-09 | | |  |  |
| Autophagy | THCA | | | NRG2 | | | -0.2013854 | | | 4.28E-06 | | |  |  |
| Autophagy | THCA | | | TSC1 | | | -0.1501772 | | | 0.00064367 | | |  |  |
| Autophagy | THCA | | | RAB24 | | | -0.3228491 | | | 8.85E-14 | | |  |  |
| Autophagy | THYM | | | NRG2 | | | -0.2085878 | | | 0.02223924 | | |  |  |
| Autophagy | UCEC | | | ULK2 | | | -0.1903518 | | | 1.00E-05 | | |  |  |
| Autophagy | UCEC | | | NLRC4 | | | -0.1486439 | | | 0.00057573 | | |  |  |
| Autophagy | UCEC | | | MAPK1 | | | 0.29653101 | | | 3.56E-12 | | |  |  |
| Autophagy | UCEC | | | ATG2A | | | 0.19878871 | | | 3.92E-06 | | |  |  |
| Autophagy | UCEC | | | FOXO1 | | | 0.14630184 | | | 0.00071253 | | |  |  |
| Autophagy | UCEC | | | TSC1 | | | -0.1788957 | | | 3.36E-05 | | |  |  |
| Autophagy | UCEC | | | RAB24 | | | -0.2328767 | | | 5.87E-08 | | |  |  |
| Autophagy | UCS | | | ATG4D | | | 0.32123412 | | | 0.01518329 | | |  |  |
| Autophagy | UCS | | | ATG9B | | | 0.38326419 | | | 0.0034556 | | |  |  |
| Autophagy | UVM | | | ATG4D | | | 0.30787623 | | | 0.0056433 | | |  |  |
| Autophagy | UVM | | | ATG2A | | | 0.37259728 | | | 0.00072171 | | |  |  |
| Autophagy | UVM | | | FOXO1 | | | -0.2347867 | | | 0.03627735 | | |  |  |
| Autophagy | UVM | | | TSC1 | | | -0.2658462 | | | 0.01739762 | | |  |  |
|  | | |  | | |  | | |  | | |  | | |
